# Porokeratoses—A Comprehensive Review on the Genetics and Metabolomics, Imaging Methods and Management of Common Clinical Variants

**DOI:** 10.3390/metabo13121176

**Published:** 2023-11-26

**Authors:** Paweł Pietkiewicz, Katarzyna Korecka, Natalia Salwowska, Ihor Kohut, Adarsha Adhikari, Monika Bowszyc-Dmochowska, Anna Pogorzelska-Antkowiak, Cristian Navarrete-Dechent

**Affiliations:** 1 Dermatology Private Practice, 61-683 Poznan, Poland; 2 Polish Dermatoscopy Group, 61-683 Poznan, Poland; kasia.korecka@gmail.com (K.K.); nataliasalwowska@gmail.com (N.S.); 3 Department of Dermatology and Venereology, Poznan University of Medical Sciences, 60-356 Poznan, Poland; 4 Department of Dermatology, School of Medicine, Medical University of Silesia, 40-027 Katowice, Poland; 5 Skin Health Center, 46027 Ternopil, Ukraine; ihor.kohut@outlook.com; 6 Medical Private Practice, Pokhara 33700, Nepal; adarshaa108@gmail.com; 7 Cutaneous Histopathology and Immunopathology Section, Department of Dermatology, Poznan University of Medical Sciences, 60-356 Poznan, Poland; mbowdmo@ump.edu.pl; 8 EsteDerm Private Dermatology Clinic, 43-100 Tychy, Poland; a.pogorzelska.antkowiak@gmail.com; 9 Melanoma and Skin Cancer Unit, Department of Dermatology, Escuela de Medicina, Pontificia Universidad Católica de Chile, Santiago 8331150, Chile; ctnavarr@gmail.com

**Keywords:** porokeratosis, mevalonate–isoprenoid pathway, genetics, dermatoscopy, ultraviolet radiation, reflectance confocal microscopy, malignancy, treatment

## Abstract

Porokeratosis is a heterogeneous group of keratinising disorders characterised by the presence of particular microscopic structural changes, namely the presence of the cornoid lamella. This structure develops as a consequence of a defective isoprenoid pathway, critical for cholesterol synthesis. Commonly recognised variants include disseminated superficial actinic porokeratosis, disseminated superficial porokeratosis, porokeratosis of Mibelli, palmoplantar porokeratosis (including porokeratosis palmaris et plantaris disseminata and punctate porokeratosis), linear porokeratosis, verrucous porokeratosis (also known as genitogluteal porokeratosis), follicular porokeratosis and porokeratoma. Apart from the clinical presentation and epidemiology of each variant listed, this review aims at providing up-to-date information on the precise genetic background, introduces imaging methods facilitating the diagnosis (conventional and ultraviolet-induced fluorescence dermatoscopy, reflectance confocal microscopy and pathology), discusses their oncogenic potential and reviews the literature data on the efficacy of the treatment used, including the drugs directly targeting the isoprenoid–mevalonate pathway.

## 1. Introduction

Porokeratosis is a heterogeneous, isoprenoid pathway defect group of keratinising disorders, microscopically sharing a typical feature, namely, the presence of the cornoid lamella. The cornoid lamella is characterised by a parakeratotic column overlying an area of epidermal invagination, surrounded by an area of dyskeratosis and hypogranulosis/agranulosis [1]. Since the first reports of porokeratosis by Neumann [2] in 1875 and Respighi [3] and Mibelli [4] in 1889, a number of subtypes have been introduced on the basis of peculiar morphology, the distribution of lesions and histopathology. These include disseminated superficial actinic porokeratosis (DSAP)—the most common variant—porokeratosis of Mibelli (PM), disseminated superficial porokeratosis (DSP), palmoplantar porokeratosis (including porokeratosis palmaris, plantaris et disseminata [PPPD], punctate porokeratosis [PuP], linear palmoplantar porokeratosis, porokeratotic palmoplantar keratoderma discreta, spiny keratoderma, porokeratosis palmaris discreta variants), linear porokeratosis (LP) and verrucous porokeratosis (VP). Less frequent clinical variants, including follicular porokeratosis (FP), porokeratoma, porokeratotic eccrine ostial and dermal duct nevus, porokeratotic eccrine duct and hair follicle nevus, have also been described [1,5,6,7,8,9,10,11]. It is still unclear whether a number of these entities are subvariants of the main six forms or distinct entities.

For a number of clinically variable manifestations, diagnosing porokeratosis might be challenging. Dermatoscopy has proved to be an auxiliary diagnostic method facilitating this process and useful in ruling out clinical differentials [12,13,14]. The aberrated mevalonate–isoprenoid pathway is involved in the pathogenesis of various subtypes [15]. In this paper, we aim to provide a comprehensive review on the most frequent porokeratosis clinical subtypes reported in the literature, as well as their genetic traits and their potential impact on its management.

## 2. Materials and Methods

For the purpose of this review, the PubMed database was searched in August 2023 using the following terms: “porokeratosis” or “porokeratoma”. After initial screening of the titles, we selected 504 articles out of the initial 1346 papers. No limits were applied on the publication type or language. A secondary manual search of the reference lists of studies, case reports and review papers identified an extra 28 relevant papers. Only papers where there was no doubt about the diagnosis and treatment result were included in the treatment section. An additional 44 papers were added in order to provide a background on genetics and auxiliary imaging techniques. A total of 576 references were included.

## 3. Disseminated Superficial Actinic Porokeratosis (DSAP)

### 3.1. Clinical Presentation

**Figure 1:**
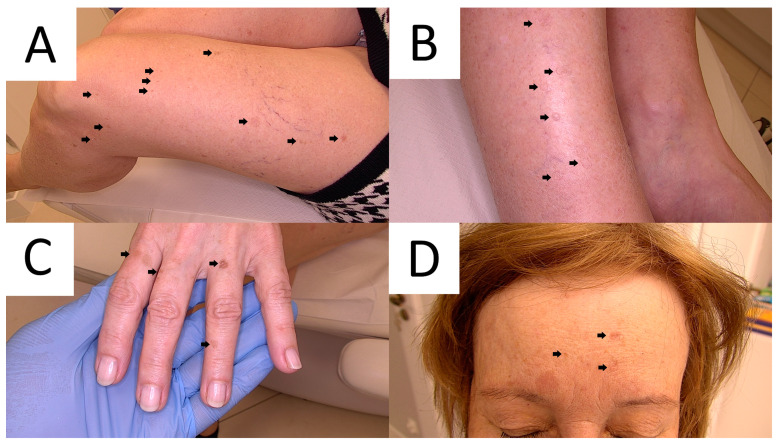
Small atrophic, annular, hyperkeratotic lesions (black arrows) distributed over sun-exposed areas (incl. upper and lower legs, dorsa of the hands, forehead) in elderly women with disseminated superficial actinic porokeratosis (**A**–**D**).

DSAP, initially described by Chernosky and Freeman in 1967 [16], usually presents as multiple pink or brown annular atrophic/keratotic macules and papules, usually with a diameter of <1 cm, located particularly on sun-exposed areas, including the extensor surface of the arms and lower extremities (Figure 1) [1,13,17,18]. In about 15% of cases, the lesions appear on the face [19]. Small papules of DSAP slowly progress into keratotic plaques characterised by a keratotic rim, which might be enhanced using exogenous pigmenting agents, such as povidone–iodine [20], fake tan lotion [21], gentian violet [22,23] but also permanent marker or ink. Skin lesions in sporadic form usually develop in the third and fifth decade of life [1,18,24], but familial DSAP usually manifests at a younger age (3rd–4th decade) [19,24]. Female predominance has been reported [25]. Ultraviolet light is one of the exacerbating factors [1,26,27]. Rarely, DSAP develops bullous [28] or prurigo nodularis-like forms [29]. There are reports of non-actinic DSAP in immunocompromised patients (organ transplant recipients, oncological patients, HIV-positive patients and others) [28,30,31,32,33,34,35,36,37], pyoderma gangrenosum [34], pseudoxanthoma elasticum [38], dermatomyositis [39], systemic scleroderma [40], Sjögren’s syndrome [41], in combination with glioblastoma and Lynch syndrome [42] and during hydroxyurea treatment [43,44].

DSAP occasionally coexists with LP as a form of type 2 segmental involvement [1,45,46]. Common coexistence with LP (multiple cases) [5,7,9,45,46,47,48,49,50,51,52,53,54,55,56,57] and occasional coexistence with VP (5 cases) [9,58,59,60,61], PM [62] and PPPD [46] was also reported. Such cases are likely caused by second-hit mutations in the monoallelic gene carriers [63]. Very frequently, DSAP is misdiagnosed as actinic keratosis, psoriasis, nummular dermatitis, lichen planus, granuloma annulare, tinea corporis or even xerosis [14,64]. Having a high index of suspicion and including DSAP in the differential diagnosis of lesions on sun-exposed areas is critical.

### 3.2. Dermatoscopy

**Figure 2:**
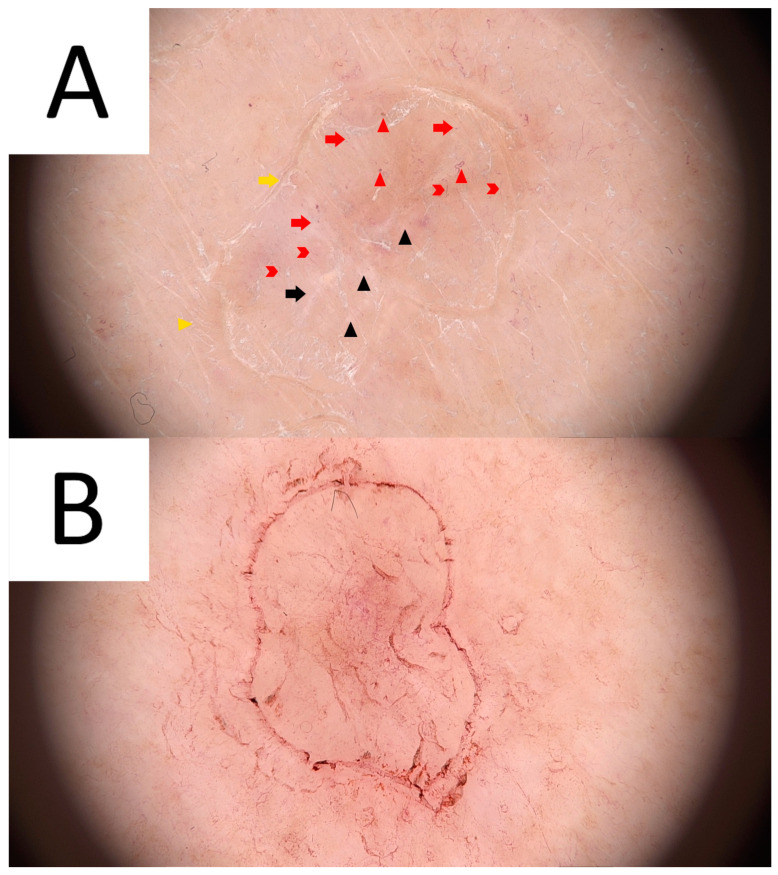
Dermatoscopy of disseminated superficial actinic porokeratosis (magnification 20×): central atrophic pink–tan area with polarising-specific white lines (black arrow), small white areas (black arrowheads) and vascular polymorphism (dots, linear serpentine and linear glomerular vessels; red arrows, red arrowheads and red V-shaped arrows, respectively), surrounded by a continuous yellowish double-edged keratin rim (yellow arrow). Radially arranged peripheral scaling (yellow arrowhead) can be observed on both sides of cornoid lamella (**A**). “Ink test”: Colouring the lesion and wiping out excessive pigment enhances the visibility of the keratotic rim, especially in non-obvious cases (**B**). In this case, brown pen ink was used, yet whiteboard marker or gentian violet can also be used.

The majority of publications concerning the dermoscopy of porokeratosis are case reports [14,65,66,67,68,69]. In 2021, members of the International Dermoscopy Society performed a retrospective cohort study of 77 patients from 11 different countries. The most common dermatoscopic features of DSAP reported in the study included a keratin rim and the presence of associated structures (vascular and non-vascular) within the rim [14,70]. This keratotic rim corresponds to cornoid lamella and proved to be a highly specific clue, present in almost all DSAP cases (Figure 2) [14,66,67,68,69,71]. Among the vascular structures, dotted or glomerular vessels were the most common findings, especially on the lower extremities [14,65]. Additionally, non-peripheral scales were reported to be present in around half of the cases, and these were the predominant non-vascular clues observed within the keratotic rim. Moreover, dermatoscopy of DSAP might reveal grey–brown dots or pigmentation along the keratotic rim [14,65,68,69], polarising-dependent shiny white structures [14], linear serpentine and linear serpentine branching vessels, polymorphous vessels and peripheral vascularisation [14,67,69]. Some of these latter findings (e.g., polymorphous vessels and shiny white structures) might be concerning for cutaneous malignancy. Thus, some of these cases might require a biopsy (see below). Even though both DSAP and PM were reported to share all the abovementioned clues, DSAP demonstrated fewer blood spots and erosions, whereas the brown pigmentation in DSAP was lighter than in PM [14]. A pigmented variant of DSAP has also been described [13,72,73]. Its frequency was estimated to be 25.8% in a retrospective study from New Zealand [72]. So far, only a single dermatoscopic report on that variant exists, with a well-demarcated, roundish, hyperpigmented periphery composed of black dots and a central clearing [13,73].

### 3.3. Confocal Microscopy

**Figure 3:**
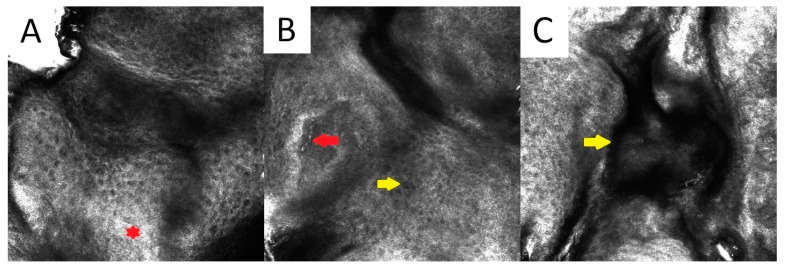
Reflectance confocal microscopy of disseminated superficial actinic porokeratosis: epidermal layer with parakeratosis (red asterisk) (**A**), elongated vessels in the lower parts of the epidermal layer (red arrow) and spongiosis (yellow arrow) (**B**), hyperkeratosis in the epidermal furrow (yellow arrow) (**C**).

Confocal reflectance microscopy (RCM) is useful in DSAP, as confocal findings match the horizontal histological sections (Figure 3) [1,74,75]. The absence of a physiological honeycomb pattern in the central aspect of the lesion and peripheral parakeratotic columns (cornoid lamellae) with focal keratinocytic atypia, surrounded by the regular honeycomb pattern of the normal skin, were noted using RCM [74,76]. RCM has been used to differentiate DSAP from psoriasis [76] and actinic keratosis [77].

### 3.4. Pathology

**Figure 4:**
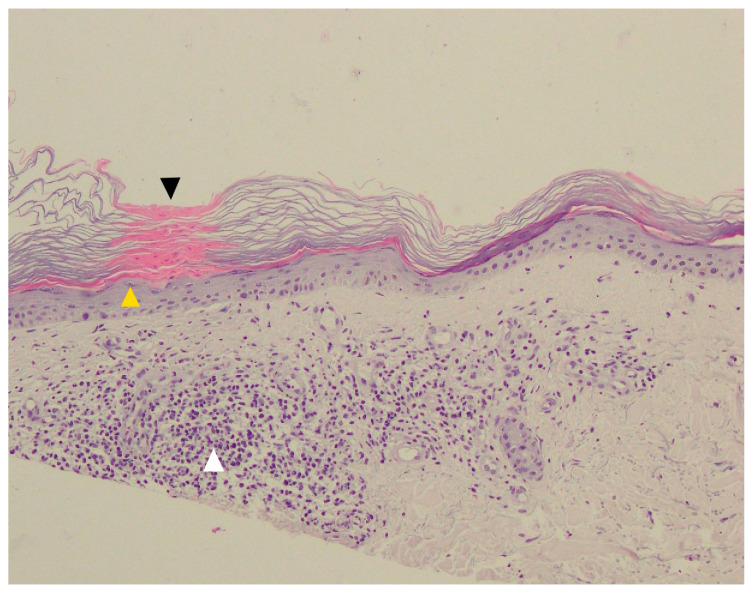
Pathology of disseminated superficial actinic porokeratosis shows cornoid lamella (black arrowhead) along with typical diffuse epidermal atrophy, agranulosis, basal keratinocyte vacuolisation (yellow arrowhead) and superficial inflammatory infiltrate in the upper dermis (white arrowhead) (original objective magnification 20×).

Due to its typical clinical presentation and current advances in dermatoscopic imaging, skin biopsy in DSAP is rarely performed, mainly in lesions suspected of malignant transformation. Optimally, skin biopsy should be obtained from the border of skin lesion. As in all porokeratosis subtypes, histopathology in DSAP reveals a column of parakeratotic cells called the cornoid lamella that correlates with the characteristic keratotic rim (Figure 4). In some DSAP cases, the cornoid lamella can be doubled [1]. Parakeratosis can develop according to two mechanisms—via the disruption of keratinocyte maturation or with accelerated upward migration [78]. It was demonstrated that the keratinocytes located below this parakeratotic column undergo accelerated programmed death, which has been linked to disrupted expression of loricrin and filaggrin at the cornoid lamella level and the absence of or a reduction in the granular layer [79]. On the other hand, keratinocyte hyperproliferation was shown not to be indispensable for the formation of the cornoid lamella, as the proliferation rate beneath it is normal [80]. It should be noted that the cornoid lamella might be incidentally present in other non-porokeratotic lesions like lichenoid keratosis and HIV-associated epidermodysplasia verruciformis [81,82]. Loss or thinning of the granular layer, the presence of basal and/or spinous keratinocyte vacuolisation and sometimes atypia beneath the cornoid lamella, focal dyskeratosis in the spinous layer, diffuse epidermal atrophy and spongiosis are among the characteristic pathology features in DSAP [1,71]. Solar elastosis and dilated papillary vessels accompanied by superficial band-like lymphocytic inflammatory infiltrate (lichenoid/interface dermatitis pattern) in the central aspect of the lesion may be present in some instances [1,19,74]. The latter should be differentiated from lichen planus-like keratosis (benign lichenoid keratosis), which never features cornoid lamellae [1,82]. Pigment incontinence and upper dermal melanophages located below the cornoid lamella were noted in a pigmented form of DSAP [13]. Focal epidermal necrotic changes or blistering are very rare [1]. In one case of prurigo nodularis-like DSAP, apart from the typical cornoid lamella, agranulosis and interface dermatitis, the authors noted hyperkeratosis, irregular acanthosis, dermal perivascular mononuclear cell infiltrate and increased dermal collagen, particularly in the dermal papillae [29]. Some cases may feature dermal amyloid deposits [1,83,84,85].

### 3.5. Genetics and Epigenetics

DSAP is inherited as an autosomal dominant pattern with a decreased penetrance of 22% [86], yet many cases result from new post-zygotic mutations [9,87]. The delayed expression of the disease (contrary to PM) is probably due to mutational events caused by a frequent sun exposure, which makes it a heterogeneous condition. So far, four DSAP genes have been identified using various methods, including genome linkage mapping, microsatellite polymorphism analysis and polymerase chain reaction studies [9]: *DSAP1* (*MVK*; reportedly linked to a cluster of loci at 12q24.1–24.2, which includes also the *SART3* candidate gene) [88,89], *DSAP2* (linked to the 15q25.1–26.1 region, which includes both *SSH1* and *ARPC3* candidate genes) [90,91,92], *DSAP3* (1p31.3-p31.1 region) [93], *DSAP4* (16q24.1–24.3 region) [94] and one more candidate gene suspected to be involved in pathogenesis, namely *DSAP8* (*SLRC17A9*) [95].

**Figure 5:**
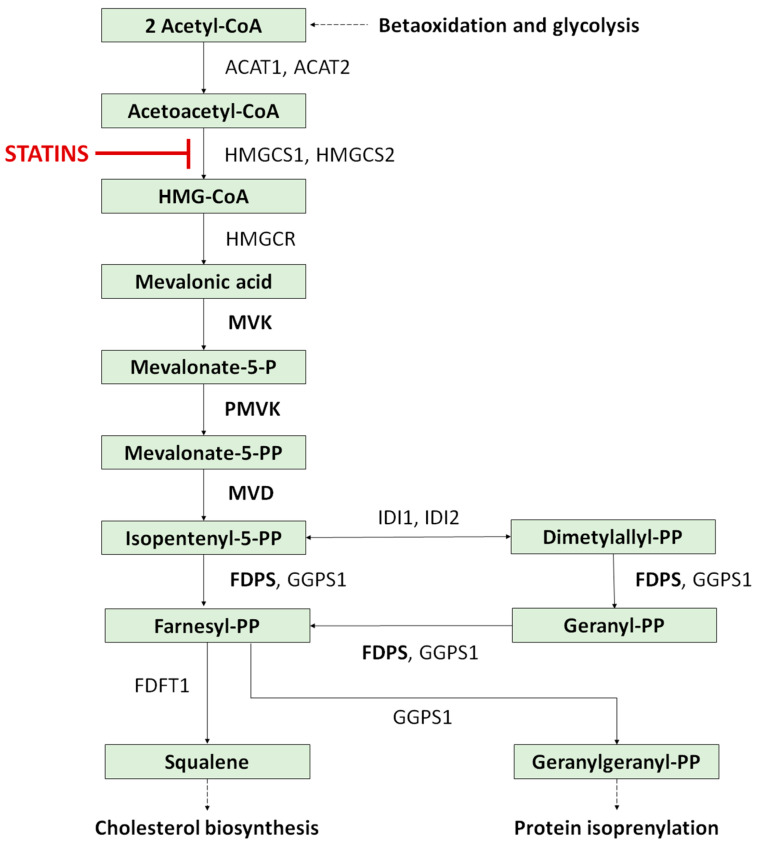
The mevalonate–isoprenoid pathway involved in pathogenesis of porokeratoses. Legend: ACAT—Acetoacetyl-CoA thiolase, FDPS—Farnesyl diphosphate synthase, FDFT—Farnesyl diphosphate farnesyltransferase, GGPS—Geranylgeranyl pyrophosphate synthase, HMGCoA—3-hydroxy-3-methylglutaryl-CoA, HMGCR—3-hydroxy-3-methylglutaryl-CoA reductase, HMGCS—3-hydroxy-3-methylglutaryl-CoA synthase, IDI—Isopentenyl pyrophosphate isomerase, MVD—Mevalonate-5-pyrophosphate decarboxylase, MVK—Mevalonate-5-kinase, PMVK—Phosphomevalonate kinase.

At least four genes (*MVK*, *PMVK*, *MVD* and *FDPS*) have been described to play a role in the mevalonate–isoprenoid (HMG-CoA reductase) pathway (Figure 5) [15,96], which is important in the synthesis of sterols, isoprenoids, cholesterol, lipoproteins and steroid hormones [97], and modulates cell differentiation, growth and apoptosis, likely playing a role in nuclear retention (parakeratosis) in keratinocytes [15]. *MVK* (mevalonate kinase) encodes the peroxisomal enzyme mevalonate kinase, a key enzyme in isoprenoid and sterol synthesis, expressed in skin keratinocytes, where it participates in the regulation of calcium-induced keratinocyte differentiation and is involved in protection from UV-A-induced apoptosis [96,98,99]. *PMVK* (phosphomevalonate kinase gene) encodes a peroxisomal enzyme converging from the galactokinase, homoserine kinase, mevalonate kinase and phosphomevalonate kinase family of ATP-dependent enzymes. *PMVK* catalyses the conversion of mevalonate 5-phosphate into mevalonate 5-diphosphate, which is the fifth step in the mevalonate pathway of isoprenoid biosynthesis [98]. *MVD* (mevalonate diphosphate decarboxylase gene) is responsible for catalytic reactions in the ATP-dependent decarboxylation of mevalonate [98]. *FDPS* (farnesyl diphosphate synthase gene) encodes an enzyme that catalyses the production of geranyl pyrophosphate and farnesyl pyrophosphate from isopentenyl pyrophosphate and dimethylallyl pyrophosphate. The final product of this reaction—farnesyl diphosphate—is a key compound in cholesterol synthesis and is a ligand or an agonist for certain hormones and protein reactions. Alterations in the aforementioned genes, causing the downregulation of the corresponding protein expression, have been reported to produce mevalonate deficiency, resulting in abnormal apoptosis in the keratinocytes.

MVK deficiency is an autosomal recessively inherited disease that has three different clinical presentations: DSAP, hyper IgD syndrome and mevalonic aciduria [78,96,100,101,102,103,104]. It was reported that the DSAP severity closely correlates with the MVK level [96,105]. Such mutations lead to the accumulation of upstream metabolites and the insufficiency of the end product of the pathway (which is cholesterol—a well-known component of the functional skin barrier), and impair keratinocyte maturation. Analysis of genomic variants of the mevalonate pathway in porokeratosis showed that the most frequent mutations found in DSAP were in the *MVK* (26/130 patients) and *MVD* (56/130 patients) [15]. At least one mutation of the mevalonate pathway gene was found in 98% (60/61) of familial cases of DSAP and 73% (53/73) of sporadic cases [15]. It was suggested that the *MVD* and *FSPS* mutations tend to produce more superficial DSAP than those seen in patients with mutated *MVK* and *PMVK* [15]. Of note, germline *FDPS* mutations were recently confirmed in a Chinese family with concurrent Lynch syndrome [43]. *MVK* mutations were identified in 33% (19/57) of familial and 16% (9/57) sporadic DSAP cases [99]. Interestingly, *MVK*’s underexpression is not only linked to compromised protein prenylation and geranylgeranylation (resulting in dysregulated inflammasome function) but also to an increased apoptotic rate [106,107].

*SART3* (squamous cell carcinoma antigen recognised by T-cells 3) encodes nuclear-restricted protein Tip110, a pre-mRNA splicing factor that interacts with oncogenic ubiquitin-specific peptidase 15 (USP15), regulating cell proliferation and the transformation of epithelial cells, and is indirectly involved in the regulation of proinflammatory responses via the modulation of NF-κB activity [108,109,110]. *SART3* was shown to play a role in regulating interactions between polymerase η and its partner RAD18, involved in UV-induced DNA damage protection [109]. This link could explain the photodistribution in DSAP. *SSH1* (slingshot homolog) encodes slingshot protein phosphatase 1 implicated in the regulation of actin cytoplasmic cytoskeleton dynamics and organisation via its stabilisation and bundling [91,111]. *ARPC3* (Actin Related Protein 2/3 Complex Subunit 3) encodes a protein complex subunit participating in actin polymerisation and bundling, thus actively involved in cell motility [112]. In regard to UV association in DSAP, this Arp2/3 complex has been shown to modulate gene transcription and the repair of damaged DNA [113]. *DSAP8* codes a gene-encoding vesicular nucleoside transmembrane protein transporter implicated in the storage and exocytosis of adenosine triphosphate [101]. Missense mutations in that gene have been identified in a small number of DSAP pedigrees.

### 3.6. Treatment

Although multiple treatment options for DSAP have been proposed, due to the relative rarity of this entity, randomised clinical trials are lacking and the majority of data are based on case reports or small case series. No therapeutic consensus has been reached, as no method has been found to achieve an acceptable effectiveness and been approved by the authorities. Ideally, enzyme replacement therapies would correct the culprit enzyme deficiency [114,115,116]. Therefore, management should depend on the accessible tools and the number of lesions and their distribution, and should be tailored to the demand of the situation.

The efficacy of diclofenac 3% gel was assessed on a series of 25 DSAP patients. Eight DSAP patients were treated twice daily for six or more months with overall good tolerability, yet only two patients achieved a clinical response, while only one achieved am overall response after six months [100]. A second larger prospective multicenter study involved 17 patients (twice daily for 3–6 months) [117]. A decrease in the target area lesions was noted in 53.8% of patients after 3 months and 30% in 4 months. The treated area presented 9% fewer lesions than the control in 6 months. In this study, the subjective effectiveness of diclofenac 3% gel was rated 4/10, whereas comfort and elegance 7/10 by the patients. Dermatitis and erythema were the only side effects reported (4 and 1 patients, respectively). A single case report of DSAP treated using topical diclofenac 3% gel (12 weeks) reported no effect [118].

Photodynamic therapy (PDT) was investigated in 30 patients with DSAP. The results of PDT utilising 5-aminolevulinic acid (ALA) in a series of three DSAP patients were not promising (effectiveness observed in one of three patients proved to be transient) [119,120,121]. Slightly better results were achieved with the use of methyl aminolevulinate (MAL) in one DSAP patient, possibly due to higher lipophilicity and better penetration to the cells [122,123]. In a larger series of 13 DSAP patients treated with MAL, 19% of them achieved a >75% reduction in the lesions [124]. In another smaller case series treated using MAL-PDT (mean 5.5 sessions, every 15 days), only a slight reduction in roughness was noted [125]. In a series of three DSAP patients, MAL-PDT (1–2 courses at a 4-week interval) was compared to cryotherapy, reporting that the effectiveness of the former was very poor (two non-responders and one patient with a 5% drop in lesion count at week 4) [126]. MAL-PDT was described to be ultimately unsuccessful in DSAP (33.3% effectiveness) and characterised by an unfavourable safety profile (pigmentation disorders, inflammation, erythema and discomfort) [121]. Salas et al. and Guillen et al. reported no recurrence after 10 months and 1 year, respectively [123,124]. PDT utilising hypericin (St. John’s wort-derived herbal photosensitiser) does seem promising. It was administered in two DSAP patients, with only one achieving a partial response [127].

Radiotherapy has been investigated in a series of 27 patients. Complete clearance in seven out of eight DSAP patients treated using superficial Grenz rays was noted, with typical transient post-radiation side effects that resolved in 4 weeks [128]. At least 50% effectiveness was reported in a group of 17 DSAP patients, with transient erythema, itching and a burning sensation [129]. These adverse reactions can be supplemented by a sunburn-like reaction, yet the tolerability of this modality seems to be good [130]. A single report on the use of high-dose-rate iridium-192 brachytherapy in one DSAP patient shows that this method can also be useful in selected limited areas and well tolerated [131]. However, clinicians should be aware of the long-term side effects of the different forms of radiotherapy, including pigmentation disorders, alopecia, skin atrophy, telangiectasia, fibrosis and secondary skin malignancies [17,128,132]. On the other hand, radiotherapy might be employed to treat DSAP with concomitant skin malignancy or even skin field cancerisation. There is a single report on the successful use of volumetric modulated arch therapy that is suited for wide, curved surfaces with a homogenous dose [17].

Reports on the use of topical vitamin D3 analogues are limited to single cases of DSAP. The results seem to be promising, yet have not been confirmed in randomised trials. Monotherapy or combination treatment including topical vitamin D3 analogs have been reported to be effective: calcipotriol 0.005% twice daily [133,134], tacalcitol 0.002% or 0.004% [135,136], calcipotriol 0.005%/adapalene 0.1% gel [137], calcipotriol/betamethasone gel [138] and CO_2_ laser with tacalcitol 0.002% ointment [139]. A combination of calcipotriol and 5-fluorouracil (5-FU) provided a moderate effect (some flattening of the lesions) [71].

Topical imiquimod 5% cream was used in a single case of DSAP (5 days per week for 4 weeks) leading to complete remission after 2 months [140]. An almost complete response with imiquimod (3 days per week for 24 weeks and once-weekly maintenance therapy) was achieved in one case [141]. The authors reported overall improvement in the context of the colour, thickness and texture of the DSAP lesions, with typical side effects of the therapy (crusting, erythema, itching) and no recurrence after 2 years. In a case series comparing the effectiveness of imiquimod (3 days per week for 8–20 weeks) and/or MAL-PDT to cryotherapy, imiquimod had no impact on the lesion count in all four patients, even though the lesions became less rough and less irritable. The treatment was inferior in effectiveness to cryotherapy [126].

There is a single case report on the successful use of ingenol mebutate 0.05% gel in DSAP [142]. Nonetheless, the drug is no longer available.

5-FU 5% gel was used under occlusion using zinc-oxide-impregnated bandage as a chemo-wrap in one patient [86]. The treatment lasted for 3 months (wraps were changed weekly); it was safe and well tolerated and achieved a satisfactory cosmetic outcome. 5-FU 5% solution (unknown regimen; 3 months) was applied in one prurigo nodularis-like DSAP case, yet the treatment was unsuccessful [29]. Systemic 5-FU was administered in one DSAP case due to breast cancer, providing a satisfactory outcome for the skin lesions [143].

Chemical peels with 50% glycolic and 25% salicylic acid used in a two-layer technique (mean of three cycles every 6–8 weeks, with 1–2 areas treated per cycle, and each area treated at least three times) showed satisfactory results and a good safety and tolerability profile in a series of five non-immunosuppressed DSAP patients [105]. A combination treatment with 70% glycolic acid and a 5-FU 5% solution applied every 2 weeks for a duration of 4 months (eight sessions) followed by 18 months of topical retinoid acid 0.05% cream provided only temporal clinical remission in one DSAP patient (15 months) [144].

There are scarce data on the use of vitamin A derivates in DSAP. Topical tretinoin 0.1% once daily was modestly effective in one case of DSAP/LP [45], but ineffective in another [145], whereas topical tazarotene 0.1% gel used once daily provided a complete remission in 4 weeks in four patients [146], and was ineffective in one [147]. The use of oral retinoids in the treatment of DSAP appears to be promising but there are not enough data on their efficacy and safety. There is only a single report on the treatment of DSAP using acitretin in an adolescent boy with graft-versus-host disease [148]. The initial dose was 0.3 mg/kg daily and was tapered to 0.3 mg/kg every other day after achieving complete remission (4 weeks), then continued for 3 months, and for the next 14 months, further reduced to twice a week with good control of the disease. Alitretinoin was reported to be effective in two patients (10 mg daily; 4- and 7-month treatment) and well tolerated [149]. The effectiveness of oral etretinate (0.75–1 mg/kg or 50–100 mg daily; 8-week treatment) was good to excellent in three cases [150,151,152], moderate in two cases [151] and none in one (75 mg/day; 3 weeks) [153]. In one case, it led to hair loss and the development of digitate keratoses that subsided after treatment discontinuation [152]. In two other reported cases (1 mg/kg/day; 3–6 weeks), the treatment was poorly tolerated, and facilitated UV-induced disease aggravation, an increase in lesion count and intense generalised pruritus [151]. The use of oral retinoids in the treatment of DSAP requires studies on larger numbers of patients, especially regarding the prevention of non-melanoma skin cancer in high-risk patients.

Topical glucocorticosteroids combined with oral antihistamines were reported to be ineffective in one case of DSAP [118].

The use of cryotherapy was reported in 10 DSAP cases [126,147,154], even though it is seemingly widely used in porokeratoses in general [155]. In one case, it was reported to be minimally effective [147], whereas in seven, cryotherapy was shown to provide an excellent outcome (80–100% lower lesion count at week 4, and 100% at week 8 and superior to MAL-PDT and imiquimod) [126]. The authors suggested that biannual follow-up for new lesions should be recommended in treated DSAP patients.

A systematic review [121] evaluating the effectiveness of a range of laser therapies in DSAP showed complete remission achieved with this modality using a Q-switched ruby laser (two cases: 694 nm; 5.0 J/cm^2^ [156]; or 4.3 J/cm^2^; spot size of 6.5 mm [157]), CO_2_ laser (two cases; 10,600 nm; one session; 100–150 mJ; 150 Hz; 15 W; 2–3 pulses each 10 ms; defocused mode; spot size of 1 mm [157]; and five sessions at 1–2-month intervals; ring abrasion method [139]), Q-switched Erb:YAG laser (1 case; 2940 nm; 13 sessions at 2–4-week intervals; 0.3 J each, 4–10 passes) [158] and Q-switched Alexandrite laser/fractional CO_2_ laser (2 cases; 100–150 mJ; 2 Hz; spot size of 1.3 mm; scan matrix 2–7; density 3) [159]. A partial response was achieved using the Nd:YAP laser (1340 nm; seven sessions every 4–5 weeks; four passes per session; 100 mJ/MTZ; 3 ms pulse duration, 100 MTZ/cm^2^ density; 8 mm tip) [145] and the fractional 1927 nm thulium fibre laser (two cases) [147], whereas PDT/CO_2_ laser combination therapy (two cases) led to a partial response in one case and no response in the other [160]. Fractional photothermolysis was reported in two DSAP cases with partial clinical improvement in the size of DSAP lesions (3–6 sessions every 4 weeks; Erbium-doped 1550-nm laser) [161]. Although the safety profile reported in these cases was usually acceptable (mild erythema, minimal hyperpigmentation and moderate edema), larger studies defining the selection of the optimal device and settings are lacking. Even though responses to laser treatment, cryotherapy, excision or curettage have shown promising results [24], this is limited by the area affected, and the major concern is the risk of subsequent hyperpigmentation and/or scarring, so patients should be advised to apply photoprotection.

In one DSAP coexisting with ovarian cancer, chemotherapy with carboplatin and paclitaxel (every 3 weeks for 6 months) led to full remission a month after starting the treatment; however, DSAP recurred after 5 months after treatment cessation in eruptive inflammatory mode [37]. The precise mechanism of action with these drugs is unknown, yet a speculatively anti-mitotic effect on the hyperproliferative mutant DSAP keratinocytes can be expected, likely via the interruption of p53-dependent mechanisms.

Potential new DSAP treatment could target the mevalonate pathway. Lovastatin, a cholesterol-lowering agent, can cause mitochondrial damage in the neurons, microglia and monocytes, and finally can induce apoptosis [115,162,163,164,165]. As one of the end products of the mevalonate pathway, cholesterol is a key component of the extracellular lipid matrix in the stratum corneum, playing an essential role in providing and maintaining the skin function [115,166]. A combination of lovastatin and cholesterol has been suggested to act by replenishing the cholesterol and preventing the accumulation of toxic metabolites in the mevalonate pathway. Topical lovastatin 2%/cholesterol 2% ointment (twice per day; 4 weeks) provided a good clinical response (significant resolution of scaling) in one DSAP patient [116]. Interestingly, the application of lovastatin 2% ointment alone following the same regimen on previously untreated lesions led to complete remission by week 6. Interestingly, no amelioration was observed with cholesterol 2% ointment monotherapy (4 weeks) [116], supporting the active role of topical statins in the treatment of DSAP. A larger series of seven DSAP patients were treated with this formulation vs. vehicle (split-body study) twice daily for 12 weeks and evaluated with a PGA scale every 4 weeks [165]. Good or excellent effects (two patients with complete and five patients with partial remission) were reportedly achieved with no adverse effects. The promising results of a statin/cholesterol formulation was also shown in an open-label, vehicle control, split-body clinical trial with a simvastatin 2%/cholesterol 2% cream applied twice daily in eight patients with DSAP [164]. A significant reduction in lesion number (OR 0.12 [95% CI 0.01–0.72], erythema score (0.25 [95% CI 0.05–0.79]) and scale score (0.18 [95% CI 0.03–0.64]) as well as patient-reported disease activity (0.33 [95% CI 0.09–0.89]) was observed. A recent single-blinded clinical trial on 31 patients with DSAP assessed the efficacy of a combination of lovastatin 2% and cholesterol 2% cream (17 patients) compared to a 2% lovastatin alone ointment (14 patients) [167]. The disease severity (scored with DSAP-GASI [Global Assessment of Severity of Illness]) significantly decreased from week 1 to week 12 by 50.0%, from 3.08 [95% CI, 2.57–3.60] to 1.54 (95% CI, 1.04–2.05], and by 51.4%, from 2.92 [95% CI, 2.40–3.43] to 1.50 [95% CI, 0.99–2.01], respectively. Both treatment groups exhibited no significant differences at week 12, which confirmed no added value of topical cholesterol. The treatment was well tolerated with only minor adverse reports such as application discomfort, myalgia, elevated creatine kinase level and skin rash (occurring in four, two, one and one patients, respectively). The abovementioned studies provide evidence for the effectiveness of topical statins in the management of DSAP.

## 4. Disseminated Superficial Porokeratosis (DSP)

### 4.1. Clinical Presentation

Although the first report on DSP was published in 1893 by Respighi and described as an “eccentric hyperkeratosis” [3], the current name was introduced by Andrews in 1937 [168]. DSP presentation resembles DSAP, but UV radiation is not considered a trigger. Multiple erythematous or pigmented small keratotic papules bilaterally affect the extremities [169]. The disease affects both sun-exposed and unexposed areas—predominantly the trunk, acral areas and genitalia [78,170]. Oral involvement is rare [171,172,173]. The majority of cases are generally asymptomatic [174], yet about one-third present with pruritus [175]. DSP has been reported to be more prevalent in females [172], even though a study from China noted male predominance [25]. The mean age of onset for DSP is between the fourth and fifth decade of life [25,172]. Some reviews previously reported DSP onset between 5 and 10 years of age, which was likely misattributed and referred to without confirmation [68,78,176].

Facial superficial porokeratosis, with lesions limited to the facial area, is believed to be a DSP subvariant [177]. An inflammatory or pruriginous DSP subvariant, called eruptive pruritic papular porokeratosis or eruptive disseminated porokeratosis (EDP), has been distinguished [174,175,178,179,180,181,182,183,184]. EDP lesions are extremely pruritic and in most cases tend to subside spontaneously within a month, usually with residual annular/macular hyperpigmented patches [174,175,182,184,185]. Although this subvariant has been proposed to develop in four forms—paraneoplastic, immunosuppressive, inflammatory and other [186]—such classification could be applied to almost all variants of porokeratosis. Four DSP cases coexisting with LP (including one rare case presenting DSP, LP and PM), possibly due to a loss of heterozygosity [53,55,187,188], two cases with coexistence with PM [189,190] and one case clinically fitting the clinical presentation of DSP coexisting with VP [191] have also been reported in the literature. Other entities occurring along DSP include multiple porokeratomas [12], diffuse epidermolytic acanthoma [192] and giant facial porokeratosis in a pregnant woman [193].

The aethiology of DSP remains ambiguous but it could be an immunological response to abnormal keratinocytes, in some cases secondary to immunosuppression [30,194] (including AIDS [195], electron beam irradiation [196], chemo(capecitabine)-radiotherapy [197], hematologic disorders [198,199,200,201,202,203], organ transplantation [204,205,206,207], systemic glucocorticosteroid treatment [208,209], nephrotic syndrome [210] or drugs, esp. biologics [211,212,213,214,215,216,217], furosemide [218], antibiotics and benzylhydrochlorothiazide [180]). Other speculative triggers for DSP include acute pancreatitis [219], viral [197,220] and non-viral hepatitis [221,222] and diabetes mellitus (likely due to keratinocyte protein glycation) [137,175,191,223,224]. In a patient with diabetes mellitus treated using metformin and empagliflozin, bullous pemphigoid (BP) developed within the atrophic centres of DSP [225]. It has been speculated that diabetes mellitus or/and empagliflozin could be linked to the emergence of BP [225]; nevertheless, this phenomenon could relate to central scar-like atrophy, as BP has been reported to affect surgical scars [142,226], Wolf’s isotopic response [227] or anti-neoplastic immunity in DSP, inducing pathological autoimmune responses to hemidesmosomal components due to the bystander effect [228].

Malignant transformation in DSP (squamous cell carcinoma) has been described [188,229], possibly due to a shared genetic background with LP with known propensity to develop skin neoplasms. Moreover, there are a few papers reporting the development of DSP (esp. its ESP variant) associated with various neoplasms [182]: esophageal cancer [230], gastric cancer [169], colon cancer [197,231,232], cholangiocarcinoma [233], pancreatic cancer [210,234], ovarian cancer [235], hepatocellular cancer [220], invasive SCC [215], myelodysplastic syndrome [198,199], idiopathic thrombocytopenia [203], graft-versus-host disease [204], leukaemia [202] and diffuse large B-cell lymphoma [200]. It was suggested that the phenomenon may be related to immunosuppression via increased expression of the p53 tumour suppressor gene [220,231,233,235], or an increased concentration of TGF-⍺ stimulating epithelial cell proliferation [230]. Nevertheless, as porokeratoses as a whole are likely to be underreported, the paraneoplastic character of DSP is doubtful.

### 4.2. Genetics and Epigenetics

An autosomal dominant pattern of inheritance with varying penetrance has been suggested in DSP [78,182,231]. *PMVK* [170] and *MVK* [236] loss-of-function mutations are believed to be involved in the pathogenesis of the disease. Two DSP loci have been identified: *DSP1* (chromosome 18p11.3) [237] and *DSP2* (chromosome 12q21.2–24.21) [238]. Interestingly, the *DSP1* locus overlaps with a locus for psoriasis susceptibility (18p11) [237] and the *DSAP4* locus [94].

Upregulated epidermal CCL26/eotaxin-3 and thymic stromal lymphopoietin protein levels at the periphery of the porokeratotic lesion have been noted in one ESP case with mixed eosinophilic and basophilic infiltrate [239]. The authors suggested that this phenomenon might have contributed to non-histamine-dependent pruritogenic signalling.

### 4.3. Dermatoscopy

Five out of six reported cases of DSP (two classical ones and four EDP including one reported as pruritic follicular porokeratosis) belong to patients from Asia [12,67,174,214,240,241]. The dermatoscopic morphology of both variants resembled DSAP. The clues included a single peripheral hypo- or hyperpigmented rim of scale, perilamellar brown dots/clods and a central whitish (scar-like/atrophic) area. The vascular pattern consisted of linear serpentine or linear serpentine branching vessels [240,241]. An ink test can further facilitate the visualisation of the cornoid lamella [67]. Follicular plugging was demonstrated in one EDP case [241]. These findings require further confirmation with larger series. For the pruriginous nature of EDP, such coexistence with prurigo nodularis should always be considered. Nevertheless, the presence of a hyperkeratotic rim should be of aid in distinguishing DSP from prurigo nodularis (background hyperpigmentation and white radial lines) [240].

**Figure 6:**
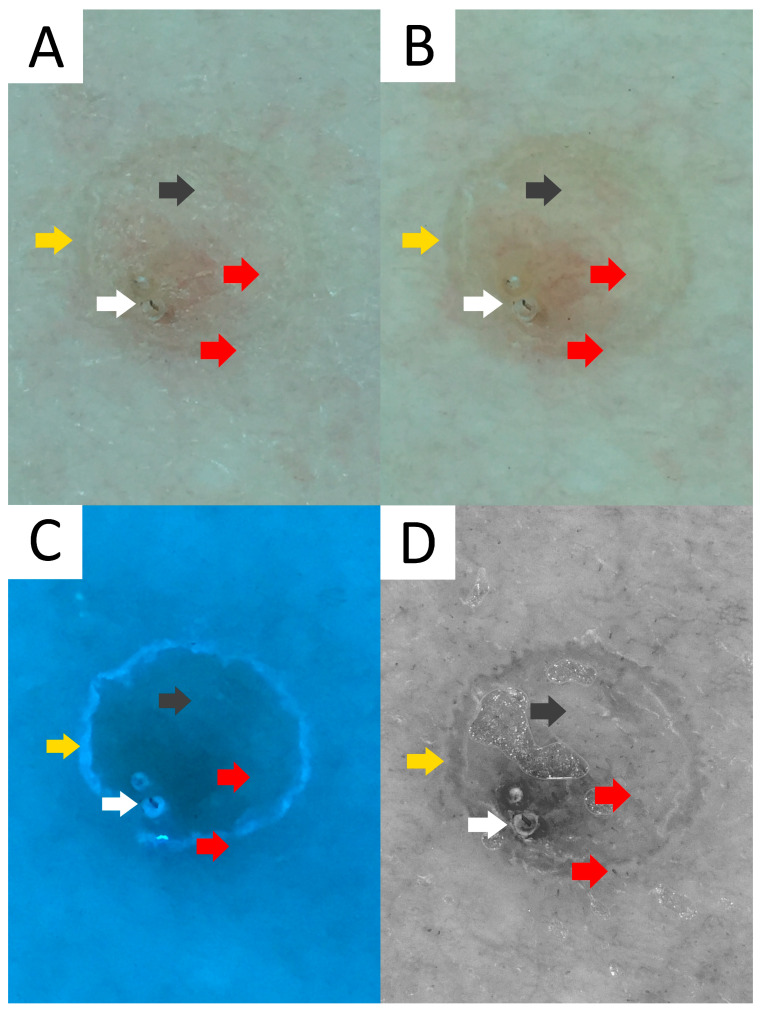
Comparison of conventional non-contact non-polarised dermatoscopy (**A**) and non-contact polarised dermatoscopy (**B**), with novel imaging techniques, namely ultraviolet-induced fluorescence dermatoscopy (UVFD; 365 nm) (**C**) and sub-UV reflectance dermatoscopy (sUVRD; 405 nm) (**D**). Note the differences in visualisation of annular keratin rim (yellow arrow), inner pink structureless area (black arrow), follicular plugging (white arrow) and vascular pattern of dots (red arrow).

Ultraviolet-induced fluorescence dermatoscopy (UVFD) is an emerging non-invasive diagnostic method in inflammoscopy based on the Stokes shift phenomenon [242,243,244,245]. Although a single case report on UVFD in porokeratosis does not mention DSP, a reported clinical presentation could support this diagnosis [67]. The authors describe the shiny “diamond necklace” appearance of the lesion’s periphery in UVFD. In this paper, the matching figure displays greenish interconnected clods. In our personal experience, the cornoid lamella along with keratin plugs may either display a bright blue colour (keratin’s excited fluorescence) in non-pigmented lesions, or become dark (melanin absorption spectrum) if the keratin rim is pigmented (Figure 6).

Sub-ultraviolet reflectance dermatoscopy (sUVRD) is another novel imaging method [246] that could be of use in porokeratoses. The method is based on physical differences in the absorption spectra among skin chromophores. Specifically, sub-ultraviolet radiation (405 nm) is absorbed by some chromophores (e.g., hyporeflective hemoglobin) and reflected by others (e.g., hyperreflective keratin). The device’s sensor collects the reflected sub-ultraviolet signals filtered out by the special filter, measures their intensity and converts it into a greyscale image (Figure 6D).

### 4.4. Confocal Microscopy

RCM in both DSP and EDP demonstrates the presence of a distinct hyper-reflective parakeratotic border at the level of the stratum corneum (without an underlying granular layer), a well-defined structured and regular honeycomb pattern of the spinous layer and numerous multiple horizontally distributed blood vessels spanning across the dermal papillae. Moreover, an atypical honeycomb pattern and an architectural disarray may also be observed [74,214].

### 4.5. Pathology

As the histological features of DSP and ESP overlap with DSAP, differentiating these two entities based on pathology seems impossible without the clinical context. The salient features include the presence of a cornoid lamella, sublamellar agranulosis, dyskeratotic keratinocytes and/or vacuolisation in the basal layer and melanophages in the upper dermis [195,214,246]. Equally, a perivascular inflammatory infiltrate (predominantly consisting of CD4+ T-helper cells mixed with Leu-6+ Langerhans cells) and mild orthokeratotic hyperkeratosis may be observed [174,178,184,240,247,248,249]. Dermal/epidermal eosinophilic infiltrate might be demonstrated in ca. 25–50% ESP cases [174,178,182,250], whereas basophilic infiltrate was reported in only one case [174]. Mast cells are absent and seemingly do not play a role in pruritus in EDP [174]. CD8+ Treg cells are suspected to drive the spontaneous regression in some EDP cases [174,251].

Keratin expression in DSP was reported to be normal whereas involucrin expression was irregularly increased (both expressing cytoplasmic expression in suprabasal keratinocytes) [195,252] and filaggrin was shown to be focally underexpressed in the granular layer under the areas of parakeratosis [195]. In the vicinity of the parakeratotic column, some keratinocytes exhibited nuclear staining for PCNA/cyclin and p53 [195,253], whereas the number of intraepidermal Langerhans cell was markedly reduced in some cases [174,195,254], and more numerous in others [249].

Eosinophilic amyloid deposition in the papillary dermis was noted in several reported cases, preferably at their inflammatory stages (both inside the annular edge and beneath the cornoid lamellae) in both sun-protected and sun-exposed areas, as a consequence of keratinocyte degeneration [84,254,255,256,257,258,259,260,261,262,263,264,265,266]. These can be stained using Congo red, pagoda red, thioflavin T or methyl violet and exhibit bright yellow fluorescence under a polarised microscope [255,258,260,261,262,263]. This phenomenon is likely underreported [263,266].

### 4.6. Treatment

There is no standard or recommended treatment for DSP, whereas the majority of modalities are reported in regard to single case reports or small series of patients. The therapeutic armamentarium does not significantly differ from the one proposed for other variants.

Physical destruction with cryotherapy [260,261],or laser treatment [29] might be useful in less widespread cases. A Q-switched Nd:YAG laser (532 nm pulse width; 5 mm spot size, 1.5-J/cm^2^ fluence; 1.4 pulse/s delivery rate; four sessions at 4-week intervals) provided complete resolution in one DSP case [267].

Systemic retinoids have been administered in few DSP patients. Oral etretinate proved to be ineffective in three DSP (1 mg/kg/day for 6 weeks; 20 mg/day for unknown duration) [151,250,255] and three EDP patients (0.5–0.64 mg/kg/day; unknown duration in one case and 1 year in two others) [179,265,268]. In one of the EDP cases, the drug was discontinued due to drug-induced renal complications. Another DSP case responded well to combined treatment with oral etretinate (20 mg/day) and topical glucocorticosteroids (unknown substance and regimen) [224]. Although a gradual reduction in itching was reported in that patient, the drug mainly reduced the severity of larger, inflammatory lesions and did not affect the smaller, non-inflammatory ones. A similar scenario was observed in another EDP patient treated with a combination of oral etretinate (20 mg twice per day for 14 days, then 20 mg/daily for 3 months), oral hydroxychloroquine (200 mg twice a day for 14 days), oral antihistamines, intravenous sodium thiosulphate administration, vitamin C injections, topical glucocorticosteroid ointment twice per day (unknown formulation) and allantoin twice per day, who achieved a moderate reduction in symptoms [269]. Oral acitretin provided a satisfactory outcome in three cases. In one patient (30 mg/day), complete remission was observed at week 4 [67], whereas one treated with 25 mg daily for 7 months [176], with an additional 0.3 mg/kg daily and tapered after week 4, achieved good DSP control at week 28 [148]. In one case, treatment failure was reported in week 2 when acitretin (10 mg daily) was combined with topical calcipotriol+betamethasone gel [203]. Adding cryotherapy led to complete remission in 4 weeks. The value of cryotherapy in managing DSP was confirmed in one more case report [148]. Topical retinoids (two cases treated with adapalene, one with isotretinoin and one with tretinoin) were ineffective [224,269,270].

Topical 0.0025% maxacalcitol [265] and vitamin D analogue cream [224,271] had poor to no role in controlling the DSP symptoms. In one case of EDP/DSP, maxacalcitol 0.0025% ointment combined with topical betamethasone led to complete remission [185], yet in our opinion, likely due to the natural burn-out of the disease rather than the treatment itself, as the lesions fully resolved in 6 months from the onset, leaving just residual hyperpigmentation.

Topical corticosteroids do not seem to affect the lesion size and count [174,178,184,241,255,258,269,272], even though in some cases might contribute to itch control [261,268,272]. On the other hand, systemic glucocorticosteroid may contribute to immune suppression, one of the risk factors for DSP [208,273], so such management should be carefully considered. Low doses of oral prednisone (15 mg/day; unknown duration) [250] and betamethasone (0.5 mg/day) [178] were ineffective in single DSP and EDP cases. Conversely, monotherapy with oral prednisone (0.77 mg/kg/day; unknown duration) [179], local glucocorticosteroid injections (6 months) [178] and a tapered dose of 40 mg/daily combined with dimetindene (unknown treatment duration) [269] led to complete resolution in three reported EDP cases. In our opinion, the therapeutic success might not have owed to the treatment but rather the natural course of the disease subvariant, which tends to subside over time [178]. In one EDP case, reported as pruritic follicular porokeratosis yet clinically fitting EDP with follicular involvement, intralesional glucocorticosteroid injections provided no clinical improvement [241].

As non-histamine-dependent mechanisms are likely to be involved in pruritus in EDP [174,239], oral antihistamines may not control the itch [178,184,250]. Nevertheless, antihistamine drugs combined with topical or oral corticosteroids have been reported to alleviate itching to some extent [261,268,269,270]. Nevertheless, in our opinion, this effect could have been achieved by any of the drugs used or simply might have resulted from natural spontaneous remission occurring in EDP, as in a scenario previously reported in one DSP and one EDP case previously resistant to such combined treatment [174,178].

Various miscellaneous drugs have been used in DSP/EDP. Topical diclofenac has been used in two cases (3% gel/unreported regimen and 1% gel applied twice daily, respectively) with poor control of EDP (at week 24) [214] and good control of DSP (reduction in scaling and pigmentation at week 4; combined with systemic etretinate) [224]. In the latter case, the lesions recurred with treatment discontinuation. Topical tacrolimus 0.1% ointment and topical polidocanol had no impact on the ESP course in one patient [269]. An excellent and rapid effect following the use of 5-FU 5% cream was demonstrated in one DSP case [274]. In two other EDP cases from India, 5-FU was introduced, yet the article did not provide any follow-up [183]. One EDSP case did not respond to imiquimod 5% cream [224]. Keratolytic treatments, such as topical 20% urea, have been used to reduce scaling, but with variable success [224,273].

Dimethyl sulphoxide (DSMO) is a protein solvent with anti-inflammatory properties traditionally used to reduce itch and subepidermal amyloid deposition in macular and nodular amyloidosis [275,276]. As DSP/EDP can be associated with subepidermal amyloid deposition and EDP with self-limiting inflammation, DSMO was utilised in two DSP cases, with clinical response in one (5-month course; no data on the regimen and concentration) and treatment failure in another [258].

Tofacitinib is a second-generation selective Janus kinase (JAK) inhibitor (particularly targeting JAK-1 and JAK-3) FDA-approved for ulcerative colitis and rheumatoid, psoriatic and polyarticular course juvenile idiopathic arthritis and investigated in many other autoimmune/inflammatory/neoplastic disorders [277]. Oral tofacitinib (5 mg twice per day; 4-week course) was reported to reduce pruritus and led to complete remission in one EDP case [270].

*Huang-Lian-Jie-Du-Tang* (also known as *Oren-gedoku-to*) is a traditional Chinese herbal drug. This medication, popular in Asia, demonstrated efficacy in managing atopic dermatitis-like lesions on a mouse model by modulating the expression of Th2-dependent cytokines (IL-4 and TNFα) [278], inhibiting the maturation of Th17 cells, recruiting Treg [279] and downregulating the skin levels of substance P (itch mediator) [280]. This medication, administered at 7.5 mg/day, has been reported to be effective in relieving itch and flattened the ESP papules in 6 weeks [250].

## 5. Porokeratosis of Mibelli (PM)

### 5.1. Clinical Presentation

**Figure 7:**
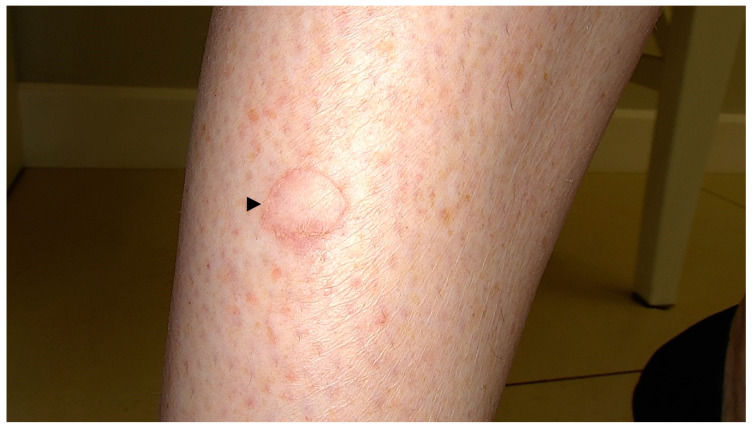
Solitary, asymptomatic annular porokeratosis of Mibelli located on an extensor aspect of a lower leg in a middle-aged woman (black arrowhead).

PM is a rare, chronic dermatosis that usually presents as a single, centrifugally spreading solitary plaque or multiple papules/macules with a central atrophy and raised keratotic borders that vary in diameter (up to 20 cm) (Figure 7) [78,248,281]. The lesions may occasionally regress [282]. Rare linear/naeviform [283,284,285], giant [10,190,286] and hyperkeratotic/verrucous [287,288,289,290,291,292] have been observed. PM subtypes have been distinguished, yet in our opinion it remains unclear whether a portion of the linear subvariants should not be rather reclassified as belonging to an LP variant. The inheritance is autosomal dominant with variable penetrance, or more commonly, random [282]. PM was reported to be two to three times more common in males than females, and more frequently occurs in Caucasians [25,78,281]. Although it may develop at any age, it usually occurs during childhood [248]. Nevertheless, in non-hereditary cases, the lesions usually appear later.

The lesions are most frequently located on the limbs, but may also affect other sites such as the palms, soles, face, scalp, mucous membranes or genitals [248,282,293,294,295,296,297,298,299,300,301]. Isolated digital lesions have also been reported [302,303]. Rarely, porokeratosis may affect the nail plate [300,304,305,306,307,308] or the phalanx [309]. In all such cases, the PM led to progressive destruction of the nail plate, which provides a rationale for bone abnormalities/anonychia screening in PM.

One of the common associations in PM is immunosuppression. The disease has been reported in patients after bone marrow transplants [310], in organ recipients [206,311,312,313] or even after the prolonged use of topical glucocorticosteroids [314]. The mechanism of this phenomenon is unclear; however, abnormal keratinocyte proliferation in porokeratosis is suspected to derive from the loss of immunosurveillance. One case of primary cardiac amyloidosis associated with PM resulting in congestive heart failure has also been described, providing a possible common pathophysiological link to disturbed immune balance [315]. PM was reported in the context of Parkinson’s disease [316], diabetes mellitus [307], viral hepatitis B [307] and C [317], vitiligo [317] and end-stage renal disease requiring hemodialysis [307]. Dermal amyloid deposits have been reported in PM lesions [292]. While their significance remains unclear, amyloid clumps may form secondary to epidermal degeneration [291]. Cases with coexisting other subtypes include DSP (3 cases) [188,189,190], DSAP [62], LP [188], VP [318] and porokeratoma [316] (one case each).

As some of the lesions may be verrucous and may mimic or coexist with psoriasis [319], confirmatory biopsy might be of aid in less typical cases [6,288,320,321]. Moreover, scaly lesions might simulate SCC, and this neoplasm has been reported to arise within some porokeratotic lesions [322,323,324,325,326]. On the other hand, cutaneous T-cell lymphoma [327] and cutaneous sarcoidosis [328] might be PM simulants.

### 5.2. Genetics and Epigenetics

PM was reported to feature genetic alterations in the mevalonate pathway (particularly affecting *MVK* and *PMVK*; see the Genetics and epigenetics section on DSAP) [15,329,330]. A cluster of loci at 18p11.32-p11.3, containing elastin microfibril interfacer 2 gene (*EMILIN2*), was hypothetically linked with PM in one pedigree [331]. As this candidate gene is involved in the regulation of cell apoptosis, its duplication and overexpression were suggested to alter the keratinocyte maturation.

Even though PM was expected to share a similar gene expression profile with SCC, due to the high incidence of this neoplasm in PM lesions (7.6%), more similarities between PM and psoriasis have been observed (highly upregulated keratin 16, S-100 molecules [A7, A8 and A9] and connexin 26). Moreover, keratin 16 has been suggested to be a potential marker of treatment response not only in psoriasis but also in PM [332].

### 5.3. Dermatoscopy

**Figure 8:**
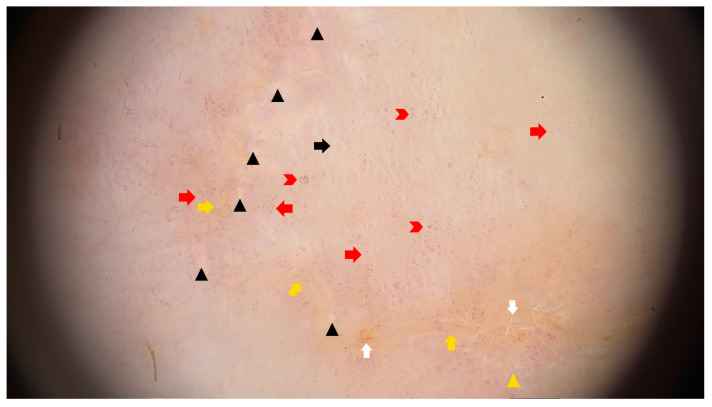
Dermatoscopy of porokeratosis of Mibelli reveals a central hypopigmented area with polarising-specific white lines (black arrow), small white areas (black arrowheads) and dotted and coiled vessels (red arrows and red V-shaped arrows, respectively) surrounded by an interrupted yellowish keratin rim with a double-edged outline (“white track”) (yellow arrows). Within this rim, follicular plugs can be seen (white arrows). Of note, dotted vessels and radially arranged peripheral scaling (yellow arrowhead) can be seen at the lesion’s outer margin (magnification 20×).

The common clues to PM are a white peripheral keratotic rim (Figure 8) (corresponding to the cornoid lamella and often double-marginated) [333] and brown clods linearly arranged within the rim [14,333,334]. Blood spots and erosions occurring throughout the keratotic rim seem to be less numerous in PM than in DSAP [14]. A helpful trick in diagnosing PM is the “furrow ink test”. Staining the surface of the skin with a whiteboard marker reveals rims alongside the peripheral bands and multiple open pores in dermatoscopy. These correspond to hair follicle openings and sweat duct pores [335].

### 5.4. Confocal Microscopy

Although confocal microscopy is rarely performed in PM, there is a single report of an linear, highly reflective structure (corresponding to the cornoid lamella in the upper dermis), whereas the epidermis seemed relatively darker and displayed a loss of the physiological architecture [336].

### 5.5. Pathology

**Figure 9:**
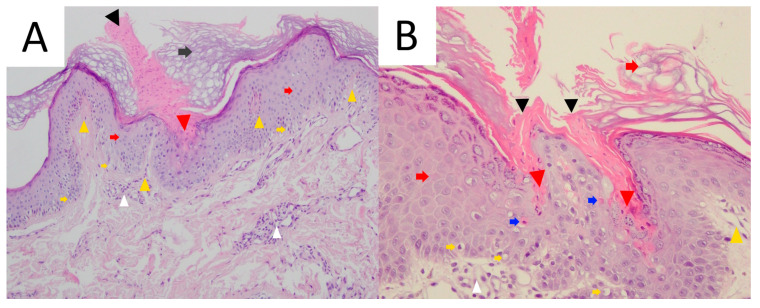
Single- (**A**) or double-edged (**B**) cornoid lamella (black arrowheads) in PM, with typical epidermal invagination (red arrowheads) and papillomatosis (yellow arrowheads) noted in surrounding skin. Hyperkeratosis (black arrows), irregular acanthosis (red arrows), basal vacuolisation (yellow arrows), single coloids (basal apoptotic keratinocytes; blue arrows), as well as mid-dermal lymphocytic inflammatory infiltrate (white arrowhead) can be observed (original objective magnification 20×).

Cornoid lamella is a histopathological hallmark of all subtypes of porokeratosis. This structure can be multiplied in PM (Figure 9) [292]. The biopsy should always involve the peripheral rim of the lesion. A typical feature for this subtype is epidermal invagination with papillomatosis noted in the surrounding skin [67,82]. These can be accompanied by hyperkeratosis, irregular acanthosis, upper and mid-dermal lympho-histiocytic inflammatory infiltrate and the presence of perivascular plasmocytes [292]. Colloids or Civatte bodies (basal apoptotic keratinocytes) have been observed in some PM cases [337,338,339]. Dermal amyloid deposits have been reported in the hyperkeratotic variant of PM in four Japanese patients, including three members of one family [291,292]. Amorphous eosinophilic amyloid deposits (suggestively of epidermal origin) extending from the upper dermis to the mid-dermis could be observed using Congo red (typical green birefringence of amyloid clots) or Dylon staining [291,292]. The authors hypothesised that such a scenario may not be rare, yet small amyloid clots may simply be missed in routine H+E staining if no supplementary immunohistochemical stains are performed [292].

### 5.6. Treatment

Various treatment options have been proposed for PM. A vast range of modalities reported in the literature assess the outcome either only in series of patients or single case reports.

PDT has been used in PM either as a single method or combined with topical treatments. MAL-PDT (1–4 sessions) was reported to achieve moderate to excellent results [122,340,341]. A combined therapy with 5% 5-FU once per day and one session of ALA-PDT led to complete clearance at week 3 and no recurrence at 6-month follow-up [342].

Cryotherapy is a promising method in PM with a good to excellent outcome; however, it comes with a risk of hyperpigmentation, scarring or atrophy [154,300,343,344,345]. Nevertheless, moderate success [293,296] and even treatment failures have also been reported [340,346]. Electrocautery was ineffective in one reported case [308].

Some PM lesions have been successfully treated surgically [302,347,348] or with skin grafts [347,349,350], but such procedures may lead to the development of hypertrophic scars, contractions and post-surgical neuralgia. The aforementioned side effects can be spared using CO_2_ laser vaporisation [297], yet unsuccessful cases have also been reported [347,348,349]. A Q-switched laser (694 nm; pulse frequency 1 Hz; two sessions at a 3-month interval) provided satisfying cosmetic results [351]. The whiting phenomenon observed after the treatment seemed to be a good indicator of effectiveness. There is one case report on the successful application of dermabrasion to PM in a dark-skinned patient. (6 × 18 mm diamond fraise attached to an acrotorque hand engine rotating at 20,000 rpm); however, mild hypertrophy and slight hyperpigmentation were noted [352].

Topical 5-FU 5% cream has been used in combination with other treatment modalities. Daily application under occlusion for 8 weeks in one PM case provided 30% resolution, preceded by a strong inflammatory reaction [346]. Topical 5% imiquimod (3 days per week for 6 weeks) treatment of this lesion finally led to complete healing. A combined topical therapy (morning 5% 5-FU and evening 5% imiquimod) was reported to be inefficient in week 4 in one PM case, yet with modification (morning 5% 5-FU and evening 5% 5-FU + 5% imiquimod), provided complete remission at week 12 [353]. No response was achieved in a case treated with topical 5% 5-FU every other day for 6 weeks [343], and another treated with 5% 5-FU for 8 weeks (unknown dosing) [342], whereas a complete response was achieved at week 5 with twice-per-day application [354]. In one PM case, 8-week therapy with 5-FU 5% cream (unknown regimen) improved the lesions [317].

Retinoids modulate the epidermal turnover and accelerate keratinocyte proliferation [355]. Complete remission of a giant subtype of PM was achieved using a combination of topical urea 12% and tretinoin 0.3% cream [286]. Topical tretinoin 0.05% cream combined with topical calcipotriol (2 months; unknown regimen) provided a partial improvement in one PM patient [300]. A significant reduction in scalp PM severity was achieved with combined topical treatment with urea (120 mg/g) and tretinoin (0.3 mg/g) [301]. Topical retinoic acid was ineffective in one case [317], but in another, provided a good effect [356]. On the other hand, topical tretinoin did not provide satisfactory outcomes in one case [347], whereas in another, it led to a reduction in hyperkeratosis without any impact on lesion size (unknown regimen; 1-year treatment) [357]. Systemic (0.5–0.7 mg/kg per day) and topical etretinate once daily for 4 weeks provided an unsatisfactory effect in one case of the hyperkeratotic PM subtype [291], whereas in another six cases, systemic etretinate was effective [320,358,359,360,361]. A severe verrucous PM case associated with HIV responded well to oral acitretin (25 mg daily), antiretroviral therapy (efavirenz/tenofovir/emtricitabine) and oral trimethoprim/sulfamethoxazole by week 16, and achieved complete clearance after 1 year of treatment [289].

Dexamethasone pulses (100 mg iv once daily for 3 consecutive days; 18 courses at 4-week intervals) provided a good response in a disseminated PM case (80% clearance and flattening of the remaining lesions) with no side effects noted [362]. Topical steroids demonstrated no effect [293].

Topical 5% imiquimod cream provided excellent outcomes in nine PM cases [140,346,353,363,364,365,366,367,368] and no response in three [340,342,369].

Although currently unavailable, topical 0.015% ingenol mebutate gel (once daily for 3 consecutive days; two courses at a 4-week interval) demonstrated success, yet the treatment led to visible atrophy and depigmentation [370].

Topical 0.7% cantharidin in the form of a thin film was applied in two PM patients (8 h exposure time), leading to blister formation and complete clearance within 1 week. Post-treatment, post-inflammatory erythema was noticed and the resolution of the PM plaques remained at 6-month follow-up [342].

## 6. Linear Porokeratosis (LP)

### 6.1. Clinical Presentation

**Figure 10:**
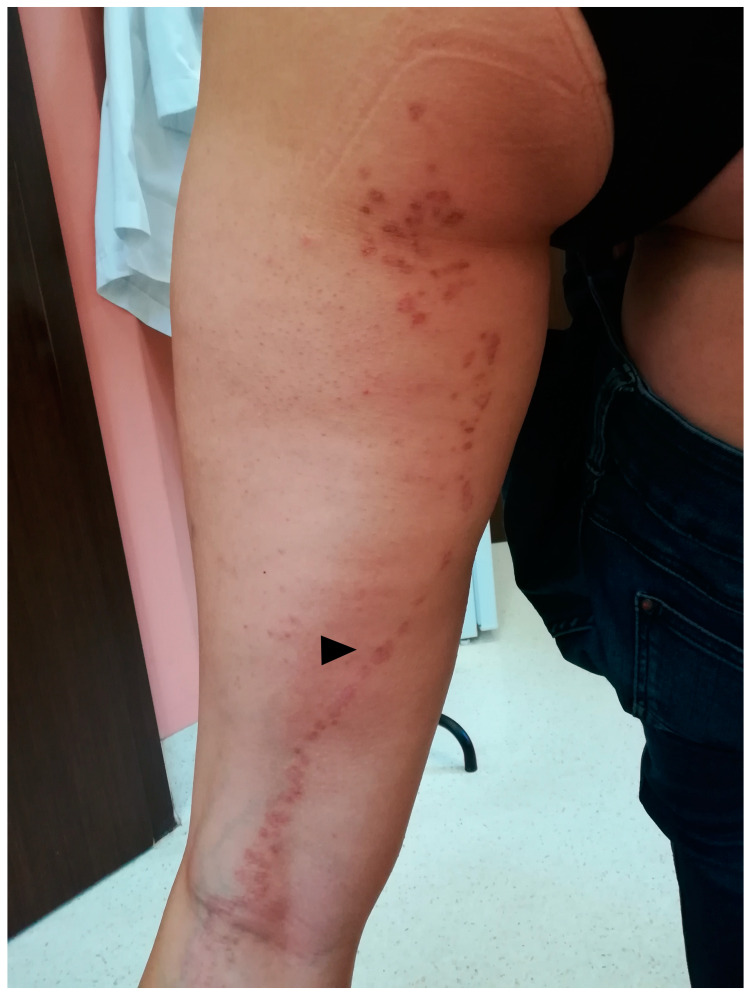
Clinical presentation of linear porokeratosis. Linear distribution of brown keratotic papules (black arrowhead) along the posterior part of the left leg in middle-aged women.

LP was first described by Goldner in 1971 as zosteriform PM [371] and acknowledged as LP by Rahbari et al. in 1974 [372]. It is the rarest subtype of porokeratosis (estimated incidence 1:200,000) and considered to be a mosaic form of DSAP [373,374]. It predominantly affects children and newborns, but may also develop in adults, including the elderly [375,376,377,378]. A slight female predominance has been reported [78]. Raised, irregularly shaped pigmented or nonpigmented macules with a blaschkoid distribution and surrounded with cornoid lamellae usually develop on the extremities (Figure 10) [114,379], whereas facial, genital and plantar distribution is rare [52,378,380]. The lesions are usually unilateral, yet bilateral or generalised LPs have also been also reported [52,114,377,381,382,383]. The plaques are anhidrotic and alopecic [384] and rarely may start as erosions [376]. A case with a giant cornoid lamella has also been reported [385].

LP may contribute to the development of a pterygium if the distal phalanx is affected [386,387,388,389,390]. There are two reports on phalangeal nail and bone abnormalities [387,391] and pseudoainhum [392] associated with LP. This subtype is regarded to be a form of post-zygotic cutaneous mosaicism and should be differentiated at the clinical level from other Blaschko-linear papulokeratotic dermatoses, both congenital and acquired, including inflammatory linear verrucous epidermal nevus (ILVEN), porokeratotic eccrine ostial and dermal duct nevus (PEODDN), lichen striatus, linear lichen planus, linear Darier’s disease, linear psoriasis, linear incontinentia pigmenti, elastosis perforans serpiginosa, ichthyosis linearis circumflexa and linearly arranged viral warts [377,378,384,393]. Liver disease [394] and Bardet–Biedl syndrome [395] were suggested to be linked to LP. This variant was reported to coexist with DSP (four cases) [53,55,187,188], LP (two cases) [46,394], PM [188], PPPD [46] and VP [396] (one case each).

It is worth mentioning that LP is a variant most commonly reported in the context of malignant transformation, usually solitary or multiple SCC [78,397]. The cumulative risk has been reported to be 11–19% with a mean latency period of 30–40 years after the disease onset [373,398]. Higher risk of malignancy is associated with longer history, a wider diameter of the LP lesions and the acral site [397,398,399].

### 6.2. Genetics and Epigenetics

Germline and post-zygotic mutations in *PMVK*, *MVK* and *MVD* (previously mentioned mevalonate pathway genes) have been associated with LP [63,114,115,400,401,402,403]. An inflammatory verrucous LP variant has been linked to *PMKV* and *MVK* mutations, whereas superficial LP to *MVD* mutations [400]. A severely inflammatory and hyperkeratotic form of LP has been reported to be associated with a *PMVK* pathogenic variant c.329C>A, p.R110Q [403]. Of note, *PMVK* and *MVK* mutations were demonstrated to be linked to Th17-dependent responses in LP [403]. *PMVK* and *MVD* have also been targeted in DSAP, which confirms the common genetic background of LP and DSAP, and there are multiple cases in the literature where both disorders coexist [5,7,9,45,46,47,48,49,50,51,52,53,54,55,56,57]. The timing of a second-hit mutation may play a vital role in the presentation of the lesions, whether they appear in childhood (LP) or later on (DSAP) [401]. In LP, the mutations happen in utero, resulting in the early presence of skin lesions, while in DSAP, the mutations are induced by prolonged exposure to UV. LP may share the same isogenetic traits and coexist with the variants PP (*MVK*) [59,396] and DSP (*MVD*) [9,11]. Genetic testing could be essential to distinguish LP from its mimics, e.g., inflammatory verrucous epidermal nevus.

The mechanism of malignant transformation in LP is likely associated with the loss of heterozygosity [399] (see Section 11 for details).

### 6.3. Dermatoscopy

**Figure 11:**
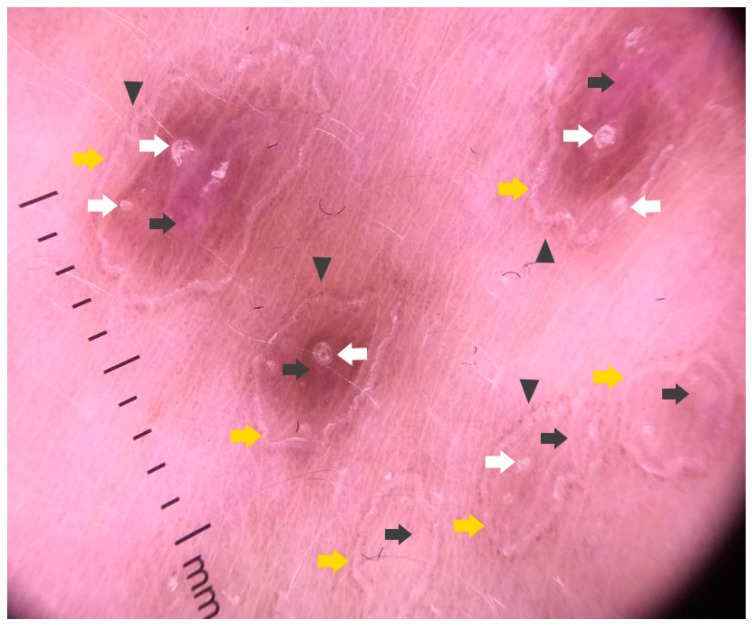
Dermoscopy of linear porokeratosis reveals white continuous rim of scales (cornoid lamella) (yellow arrows) surrounding central depigmented or pinkish–brownish areas (black arrows) with follicular plugs (white arrows). Pigmented dots can be appreciated at the outer margin of cornoid lamellae (black arrowheads).

The cornoid lamella might be very subtle in LP. Thus, in clinically non-obvious cases, thorough dermatoscopic examination might be prudent, especially considering the oncological risk in LP. In dermoscopy, linear porokeratosis may feature a white to yellow keratotic rim surrounded by brown dots or clods or white areas (Figure 11) [404,405]. Some pigmented lesions may display a blackish aspect of the inner annular ring, grey clods and hair casts in the proximity of the cornoid lamella [377]. The central aspect of the lesion was reported to feature hyperpigmented structureless area or grey-to-brown lines reticular with grey clods [377]. Smaller early LP lesions have been characterised by the presence of brown/black dots and clods at the inner side of the thin keratin rim, whereas larger ones developed outer brown/black dots and clods and inner grey/brown lines reticular lines [405]. The widest early plaques featured grey/brown dots and clods arranged in lines along both the inner and outer side of the rim [405]. In mature LP lesions, the inner area with reticular lines becomes red and then finally becomes a pinkish–white structureless area resembling a scar [405]. One report describes the dermatoscopic features of a plantar variant of LP [378]. The lesions were surrounded with pigmented dots and clods or a thick white keratotic rim, whereas a few flaky/pale white dots, clods or irregular structureless areas were noted in the central aspect of the lesion.

### 6.4. Confocal Microscopy

A single case report describes the normal stratification of the skin, a 40% reduction in the spinous layer, post-inflammatory changes and dermal neovascularisation deprived of scarring [406]. The structural features of the peripheral cornoid lamellae have not been reported.

### 6.5. Pathology

**Figure 12:**
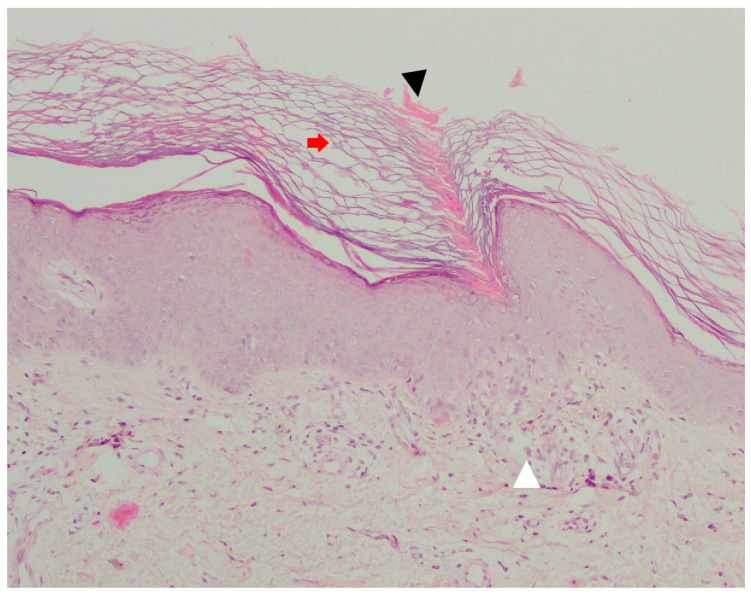
Histopathology of linear porokeratosis. A classical basket weave hyperkeratosis (red arrow) is interrupted by a single parakeratotic column delved into epidermis (cornoid lamella; black arrowhead). A sparse inflammatory infiltrate underneath the structure (white arrowhead) can be noted (original objective magnification 20×).

The cornoid lamella, a hyper/parakeratotic column of tightly packed keratinocytes, is a hallmark of the disease (Figure 12). In LP, it is usually multiplied and distributed throughout the lesion [108]. A single column of parakeratosis and folliculocentrism is rather uncommon [108,407,408]. The underlying epidermis is agranulotic, whereas the bordering epidermis is usually atrophic and features basal layer vacuolisation. Dyskeratotic keratinocytes can be present in the spinous layer, whereas necrotic ones appear beneath the cornoid lamella [377,409]. Dilated vessels, pigment incontinence (at both sides of the cornoid lamella, but not below it) and strongly IL-17A-positive mild CD4+ and CD8+ inflammatory infiltrate may be present in the upper dermis [377,378,403,405,410]. Subepidermal amyloid deposits have also been observed in this subtype [85].

### 6.6. Treatment

Since LP is not a frequent entity, the knowledge on the treatment methods of this disease is based on case reports or case series.

CO_2_ [411,412] and 585 nm pulsed dye laser irradiation [413] were reported to give satisfactory results in LP. A therapeutic success was achieved using diamond fraise dermabrasion in one patient, with partial repigmentation and no recurrence [414]. As in the other forms of porokeratosis, LP can also be managed using cryotherapy [415,416], yet some cases tend to be unresponsive to this treatment [417].

There are three reports on the use of MAL-PDT in LP [406,418,419]. Two adolescent patients underwent this treatment (2–3 sessions at 4-week intervals) after superficial skin stripping. The photosensitiser was applied under occlusion for 3 h and subsequently irradiated (energy density 37 J/cm). Regardless of a transient burning sensation during the session and remaining subtle peripheral hyperpigmentation, the outcome was satisfactory in regard to colour and scale, and the patients were reported to remain in remission at an 11–12-month follow-up [406,419]. Another adolescent responded to MAL-PDT (2 h application under occlusion; two sessions at a 3-week interval; energy density 37 J/cm), also achieving an excellent cosmetic outcome [418]. A combined therapy with ALA 20% solution (occlusion for 3 h) and pulse dye laser (595 nm; spot size 10 mm; fluence 8 J/cm^2^, pulse duration 1.5 ms) resulted in complete clearance [417].

Vitamin A derivates modulate keratinocyte maturation, providing either flattening of the LP lesions or their clearance. Thus, oral retinoids are considered by some researchers to be the treatment of choice in LP [420]. Oral isotretinoin (1.5 mg/kg for 24 weeks and 1.7 mg/kg for next 8 weeks) was reported to provide a good cosmetic effect in regard to thickness and scaling, and a moderate effect on hyperpigmentation, in a 2-month follow-up in a young female with adult-onset progressive LP [421]. Satisfactory effects have also been achieved in other cases treated with oral isotretinoin [422], acitretin [383,423] and etretinate [424,425], whereas treatment failure was reported in two using acitretin [412,426]. Treatment failure with systemic retinoids (no data regarding the drug) was reported in two LP cases [403,412].

Tretinoin 0.05% cream applied once daily was reported to be ineffective at 1-month follow-up [377] and so were topical adapalene [418] and topical tretinoin [427]. Split-body treatment with tretinoin 0.5% gel (once daily) vs. 5-FU 5% cream (twice daily) in one case report led to comparable significant clearance at week 12 and no recurrence at week 24, yet healed LP lesions displayed post-inflammatory hyperpigmentation [428]. Both treatments were characterised by mild to moderate transient side effects. Nevertheless, treatment failure with 5-FU 5% cream has also been reported [417].

Topical vitamin D analogues are supposed to modulate keratinocyte differentiation and proliferation alongside a reduction in inflammation. Topical vitamin D3 ointment (once daily for 12 weeks) has been used with moderate success in a single case of LP [390], but without success in others (3–12 months) [412,418]. On the other hand, an excellent response to a typical vitamin D3 analogue (maxacalcitol) in regard to pruritus and erythema was noted after 2 months in a single case with pruritic LP [379]. Non-histaminic itch mediators, such as thymic stromal lymphopoietin, periostin and interleukin 31, were locally upregulated, suggestive of Th2 response involvement, whereas no increase in eosinophils or mastocytes were observed in this particular case [379]. Six-month therapy with topical calcipotriene 0.005% cream and diclofenac 1% gel was reported to be ineffective in LP [429].

Topical glucocorticosteroids [379,403,412,417,426], topical urea (concentration unknown) [427] and 10% salicylic acid [379] were all reported ineffective in LP.

Topical tacrolimus 0.1% ointment provided no benefit in two LP cases [412,418], it while effectively cleared the lesions in one case [426]. Interestingly, a combined treatment with topical tacrolimus 0.1% (twice per day) and betamethasone dipropionate 0.05% ointment lead to LP clearance in 8 weeks with no recurrence in 2.5 years, and only mild residual post-inflammatory hyperpigmentation [426].

In a case of LP, topical imiquimod 5% cream (once daily, 5 days/week under occlusion) provoked a strong inflammatory response at week 3; thus, the treatment was tapered to twice per week without occlusion (extra 16 weeks). The outcome was excellent in regard to colour and scaling at both 3 months and further at 1-year follow-up with no recurrence [430].

As a cholesterol biosynthesis pathway is involved in the pathogenesis of porokeratoses, statin use has been proposed to manage LP. Topical lovastatin 2%/cholesterol 2% cream, aimed at supplementing the deficient mevalonate pathway end products, was well tolerated and moderately effective in five cases of LP [115,391,429,431]. Contrarily, a poor outcome has been reported in five cases treated with simvastatin 2%/cholesterol 5% ointment and 10% urea cream (twice daily for at least 4 weeks) [412]. Increasing the concentration of the simvastatin compound to 5% gave a superior effect at 10-month follow-up in regard to pruritus, pain and thickness in one patient [412]. One of these did not improve and one worsened. Atorvastatin 2%/cholesterol 2% ointment was reported to have no additional benefit to oral isotretinoin treatment in LP [422]. One LP patient was reported to be unresponsive to an unspecified statin/cholesterol formulation [403].

Bleomycin electrochemotherapy was reported to be successful in a single case of LP and multiple LP-associated SCCs [373]. A good clinical response, viz. complete remission of the treated SCCs and almost complete resolution of LP lesions and itching, was observed 8 weeks after the treatment, with acceptable tolerability (pain due to bleomycin). No recurrence of either SCC or LP at the treated sites was observed at 1-year follow-up.

Recently, a targeted anti-IL-17A therapy has been proposed to manage LP, as Th17-dependent inflammation has been shown to be involved in *PMVK*- and *MVK*-related disease [403]. Off-label treatment with secukinumab (75 mg s.c. every 3 weeks; for over 1.5-year duration) was demonstrated to reduce the symptoms (erythema and hyperkeratosis) within several weeks of first administration [403]. The achieved amelioration was stable and matched with a reduced DLQI (16 to 12), whereas the treatment tolerability was excellent.

## 7. Porokeratosis Palmaris, Plantaris et Disseminata (PPPD) and Punctate Porokeratosis (PuP)

### 7.1. Clinical Presentation

**Figure 13:**
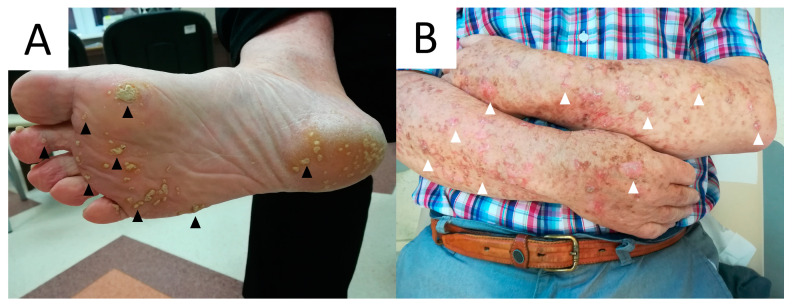
Clinical presentation of porokeratosis plantaris, palmaris et disseminata (PPPD) in elderly men who developed cutaneous squamous cell carcinoma within the lesion. The punctate keratotic lesions affected palms and soles (black arrowheads) (**A**), whereas the annular lesions were distributed in a reticulate fashion on the forearms (white arrowheads) (**B**) and lower legs.

PPPD and PuP are rare variants of porokeratosis. PPPD was first described by Guss et al. in 1971 [432] and has been referred to as “porokeratosis punctata palmaris et plantaris” or “punctate keratoderma (spiny keratoderma)”, which is a clinically distinct, autosomal dominant subvariant of porokeratosis [433,434]. Unlike other subtypes, PPPD usually presents in adolescents or young adults in their early 20s with lesions restricted to the palms and soles (Figure 13) [435]; nevertheless, congenital cases were also reported [436]. It is more frequently observed in males [433,437,438]. Subsequently, the plaques spread within months or years until the entire extremities and trunk are affected [439]. Subungual, hyperkeratotic lesions might be found on the fingernails and toenails [440], while opalescent lesions may appear on the buccal mucosa [441]. Papules on the acral locations are usually more hyperkeratotic than the ones on the trunk, which are more superficial and morphologically resemble DSAP [442]. Both DSAP and PPPD present with exacerbated, widely spread lesions; however, in PPPD, they are not only limited to sun-exposed areas. It has been reported that 25% of patients with PPPD experience pain due to plantar lesions, and another 25% of them endure exacerbations of the lesions during summer [432]. A case of PPPD presenting as multiple erythematous, hyperkeratotic papules with pruritic inflammatory changes has also been described [443]. The oncogenic potential of PPPD is proposed by a few reports on multiple SCCs developing on the palms and soles in these patients [444,445,446].

PuP is likely a PPPD subvariant, although other authors claim it is a more severe form [432,434,447]. The disease was initially reported as “punctate keratoderma” by Brown in 1971 [448], later on dubbed “punctate porokeratotic keratoderma” by Herman in 1973 [449] and finally given the current term by Rahbari et al. in 1977 [450]. This keratinising disorder clinically presents as multiple tender, linear or diffuse seed-like pits 1–3 mm in diameter filled with keratotic plugs symmetrically affecting the palms and soles [435,437,449,450,451,452], yet rare unilateral involvement was also described [453]. Differential diagnoses for PuP may include viral warts, arsenic and arsenical keratosis, punctate palmoplantar (Buschke–Brauer–Fisher) keratoderma, nevoid basal cell carcinoma (Gorlin–Goltz) syndrome, punctate porokeratotic keratoderma, spiny keratoderma (also known as music box spine dermatosis) and pitted keratolysis [451,453]. Keratin plugging of Darier’s and Cowden diseases is accompanied by other criteria to these diagnoses [453]. Chronic idiopathic hepatitis [394], renal transplantation due to chronic renal insufficiency [454] and diabetes mellitus [434] were reported to coexist with this variant.

PuP may coexist with DSAP/LP [46] and LP [394]. So far, there are no data concerning the risk of malignancy [453].

### 7.2. Genetics and Epigenetics

PPPD is an autosomal dominant entity, although there are sporadic cases with no family history of this disease [432,442,443,455,456,457], which could result from a spontaneous gene mutation or other triggers, e.g., immunosuppression or radiation. Five chromosome loci regions at 12q24.1–24.2 have been identified in a five-generation Chinese family with PPPD (18/80 members) [457]. Interestingly, an overlap between the loci for PPPD and DSAP has been reported, which may explain the clinical resemblance of these two diseases. The mutation in patients with PPPD may develop spontaneously. In a different study, *MVD* mutations were detected in three patients with PPPD [458]. Reports of PuP do not provide any data on genetic traits besides positive family history [434,435,452]. Currently, no more than 100 PPPD cases have been reported in the English literature.

### 7.3. Dermatoscopy

There is only a single dermatoscopic description of PPPD in a dark-skinned individual [437]. Dermatoscopically, both PPPD and PuP are characterised by multiple, yellowish annular structures present with a discrete white rim surrounding a central homogenous brownish clod. An “ink test” can be used to better visualise the lesions [437]. Dermatoscopic examination can be useful to differentiate PPPD from circumscribed palmar hypokeratosis [437,459]. Pitted keratolysis can be differentiated from PuP as it displays multiple coalescing plantar pits with a free edge without a central clod, and presents distinct porphyrin-related UVFD clues, namely multiple pits surrounded with coral–red scaling, a coral–red parallel ridge pattern (not seen using conventional dermatoscopy) and coral–red eccrine dots and clods [244].

### 7.4. Confocal Microscopy

No data.

### 7.5. Pathology

**Figure 14:**
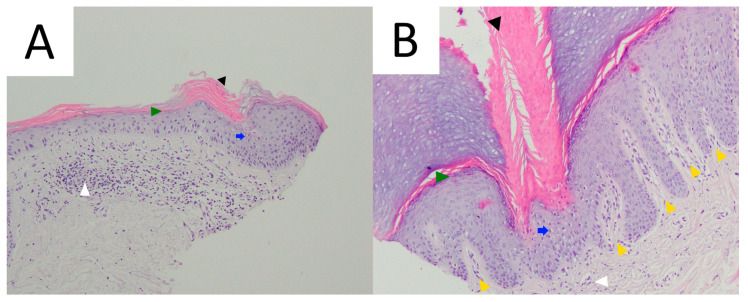
Pathology of the forearm (**A**) and plantar (**B**) lesions in a middle-aged man with porokeratosis plantaris, palmaris et disseminata (PPPD) showing cornoid lamella (black arrowheads), papillomatosis with hypogranulosis (yellow arrowheads and green arrowheads, respectively), dyskeratotic keratinocytes (blue arrows) and mild superficial dermal inflammatory infiltrate (white arrowheads) (original objective magnification 20×).

Similarly to other porokeratosis subtypes, the most prominent finding in PPPD is the cornoid lamella. Moreover, agranulosis, acanthosis, papilomatosis, dyskeratotic keratinocytes and subepidermal lympho-histiocytic inflammatory infiltrate were reported (Figure 14) [440,443,460].

The typical features of PuP encompass the cornoid lamella, hypo/agranulosis, keratin plugs, dyskeratosis and vacuolated cells [453]. Common histopathological simulants of PuP include punctate palmoplantar keratoderma, nevoid basal cell carcinoma syndrome, punctate porokeratotic keratoderma and spiny keratoderma [453]. Even though all the abovementioned disorders (along with PuP) present with keratin plugging, the first two display neither a cornoid lamella nor a thinned granular layer, and, contrary to PuP, all lack dyskeratotic and vacuolated keratinocytes [453]. Contrary to PuP, palmar and plantar pits present in nevoid basal cell carcinoma syndrome, and corynebacterial pitted keratolysis exhibits a focally reduced or absent horny layer [244,453]. A cornoid lamella may also be present in actinic keratosis and porokeratotic eccrine ostial and dermal ductal nevus, yet the former shows epidermal cytologic atypia, and the latter involves acrosyringia within the parakeratotic column [435,442]. Even though viral warts may occasionally display cornoid lamellation, no koilocytosis is ever observed in PuP [435]. Of note, Darier’s and Cowden diseases may also display keratin plugs, so the pathology should always be matched with the clinical context [453].

### 7.6. Treatment

Few case reports provide data on treatment. There are a few attempts to treat PPPD using systemic retinoids, due to their ability to regulate epidermal proliferation, desquamation and inflammation. These include isotretinoin (60–80 mg/daily or 1 mg/kg/day; 5 months course) [442,461], etretinate (tapered doses of 10–75 mg/daily) [442,445,446,447], and acitretin (25 mg/day or 0.5 mg/kg/day; 4–5 months course) [442,460], providing a good to excellent effect. However, in one case, the treatment was interrupted due to cheilitis, syncope and systemic complications. Moreover, one PPPD case was aggravated by etretinate (1 mg/kg/day) [151]. It has been speculated that systemic retinoids could serve as skin cancer prevention in PPPD [446].

A significant reduction in lesions were reported in a PPPD patient treated with imiquimod 5% cream (alternate days for 3 weeks under occlusion, then tapered to three times per week without occlusion for another 6 months) [440].

Two patients with PPPD were reported by Atzmony et al. in their case series. A cholesterol 2%/lovastatin 2% ointment was applied twice daily for 6 weeks in one patient and 8 weeks in the other. In both of them, a reduction in scaling was observed, however, without an impact on lesion count and size [115].

The literature’s data regarding the treatment modalities in PuP is scarce. Topical 2% cholesterol/2% lovastatin ointment (twice daily; 19 months) did not resolve the lesions. Nevertheless, it softened them and slowed down their recurrence [452]. Treatment with salicylic acid provided no effect [451,452].

## 8. Verrucous Porokeratosis (VP)

### 8.1. Clinical Presentation

VP (also known as genitogluteal porokeratosis) is an extremely rare variant of porokeratosis first characterised clinically in 1985 by Helfman and Poulos as “reticulated porokeratosis” [462], and later on named “porokeratosis ptychotropica” by Lucker et al. in 1995 [463] and “perianal inflammatory verrucous porokeratosis” by Stone et al. in 1999 [464]. This nomenclatural chaos is a reason for undying confusion among researchers, clinicians and patients, the motive for introducing a unifying VP term [108,465]. Nevertheless, this trend is maintained, as the terms “porokeratosis ptychotropica” or “penoscrotal porokeratosis” still remain preferable for a number of authors in clinically non-verrucous VP cases.

**Figure 15:**
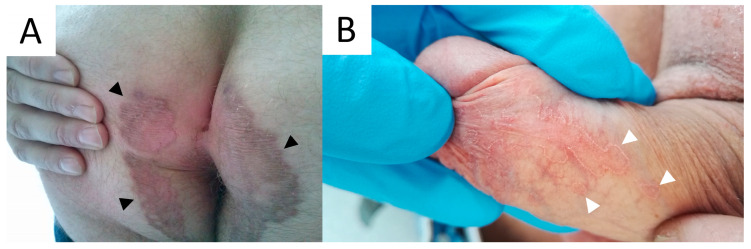
Verrucous porokeratosis. Clinical image of genitogluteal verrucous porokereratosis featuring prominent keratotic rim (black arrowheads) in a middle-aged man with a “butterfly-shaped” distribution over the gluteal region (**A**). Isolated penoscrotal verrucous porokeratosis affecting the penis shaft and preputium (white arrowheads). The patient had a 15-year history of treatment for lichen planus (**B**).

VP is clinically characterised by the presence of multiple, slowly progressing, well-defined, concentrically arranged, symmetrical reddish to brown-coloured hyperkeratotic, verrucous or psoriasiform/lichenified erythematous or keratotic papules, annular or depressed plaques associated with severe itching or burning (Figure 15A) [191,466,467,468,469,470,471,472,473,474,475]. Multiple (or, less frequently, solitary) cornoid lamellae produce thread-like, ridged, uplifted boundaries [468,469,476,477]. The lesions spread centrifugally over a 5–10 year period [465] and have a tendency towards forming small peripheral satellite lesions [478].

VP affects the anogenital area and skin folds, usually the genitogluteal region, but may also involve the scrotum, penis (including the glans and urethral meatus), vulva and anus [465,467,473,479,480,481,482,483,484,485], as well as groin and upper thighs [486]. Thus, the peculiar pattern of distribution is sometimes described as having a butterfly-shaped appearance [61,465,485,486,487,488]. The majority of gluteal lesions are adjacent to the natal cleft rather than the lateral quadrant of the buttocks [469]. A VP subvariant exclusively involving the penile shaft and anterior scrotum (penoscrotal porokeratosis) is regarded as a distinct entity by some authors (Figure 15B) [473].

The peak incidence of VP is the 3rd–5th decade of life, yet the first lesions may occasionally develop in childhood [465,473,479,489,490]. The majority of reported cases have been in males, whereas occurrence in women seems to be extremely rare [201,465,467,472,481]. The disease is reportedly aggravated by warm climates [466,467,477], friction with clothing [467,468] or scratching [467,473].

VP may mimic many dermatological conditions (esp. inverse psoriasis, but also viral warts, perianal Paget’s disease, Darier’s disease, Hailey–Hailey disease, epidermal nevus, seborrheic keratosis, lichen planus verrucosus, lichen simplex chronicus, contact dermatitis, neurodermatitis, Zoon’s balanitis, dermatophytosis, chromomycosis, paracoccidioidomycosis, squamous cell carcinoma, sebaceous carcinoma, condyloma acuminata, granuloma annulare, lichen sclerosus, morphea, verrucous cutaneous tuberculosis, leishmaniasis, acrodermatitis enteropathica and necrolytic migratory erythema), the patients usually report multiple treatment failures and are subjected to delayed diagnosis [464,465,467,469,474,477,485,487,491,492,493,494,495]. Thus, the disease is suspected to be largely underdiagnosed and some cases may be treated as sexually transmitted infections [295,465,467]. Nevertheless, there are examples of VP and STI coexistence in the literature [295].

Little is known on the pathogenesis; however, repeated traumas and defective immune surveillance have been speculated to play a role [201,484,487,490,496,497]. VP was reported to develop at sites of prior radiotherapy and injection in a HIV-positive patient [470] and non-HIV-immunocompromised patient [490]; associated with multiple myeloma treated with thalidomide and dexamethasone [201]; an allogenic bone marrow transplant due to myeloid leukaemia [496]; at sites previously treated using topical glucocorticosteroids [492] and in a patient undergoing imatinib therapy for systemic mastocytosis [487]. Five diabetic VP cases may indicate the potential role of protein glycosylation [191,467,496]. Rarely, VP may coexist with other subtypes: DSAP [58,59,60,61], DSP [191], PM [318] or LP [396]. Contrasting to PM, malignant transformation (SCC) has been reported only in a single patient [498]. Even though the oncogenic potential in VP remains uncertain, an increased vigilance towards malignancy is recommended.

### 8.2. Genetics and Epigenetics

VP almost never has any family history. However, single case reports might suggest an autosomal dominant trait [191,465,499,500]. *MVK* mutation was reported in 50% of VP patients and exclusively associated with clinical presentations featuring a giant, plaque-type lesion with a diameter of 5+ cm [15,59]. Interestingly, patients diagnosed with *MVK*-related porokeratosis demonstrate the widest range of phenotypes in terms of size and number of lesions [15,59]. Six VP cases coexisting with DSAP reported in the literature support the isogenetic background of these two variants [15,58,60].

### 8.3. Dermatoscopy

**Figure 16:**
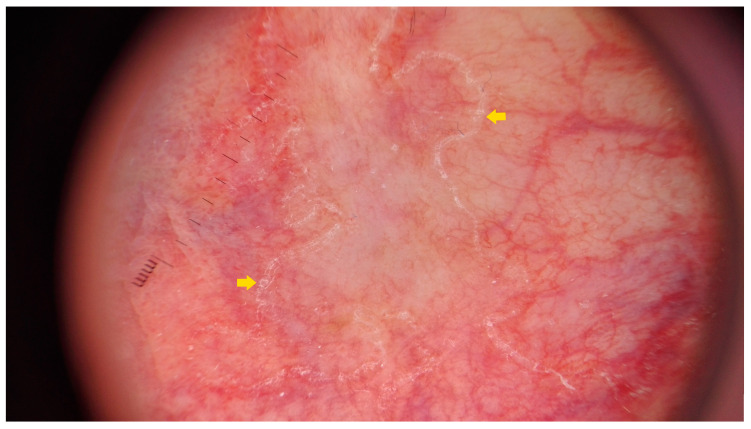
Dermatoscopy of penoscrotal verrucous porokeratosis exhibiting white keratotic rim (cornoid lamella; yellow arrows).

Dermoscopy of VP usually shows a thick peripheral hyperkeratotic rim, variable in colour (hypopigmented, brown, grey or white) typical for all porokeratoses (Figure 16). A central scar-like or hyperpigmented area, polarising-specific white four-dotted clods (rosettes), clues of scale (thick curved parallel lines/cerebriform or papillomatous-like pattern, white or brown diffuse scales, follicular plugs) and a vascular pattern consisting of non-uniformly arranged dots or clods can be noted. Pigmented dots and clods on a reddish–brown background may occasionally occur [59,466,473,486,493,501,502,503,504,505].

### 8.4. Confocal Microscopy

RCM reveals a well-defined, peripheral rim appearing as a hyper- and/or hyporeflective amorphous structure abruptly interrupting the epidermis alongside the loss of the physiological honeycomb pattern. These findings resemble the RCM pattern demonstrated in DSAP and PM [486].

### 8.5. Pathology

**Figure 17:**
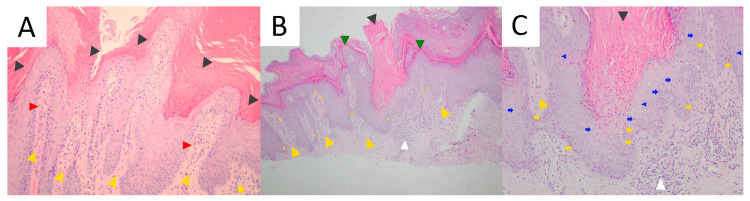
Pathology of verrucous porokeratosis. Multiple cornoid lamellae (black arrowheads) in a hypertrophic, irregularly papillomatous epidermis (yellow arrowheads), dilated papillary vessels (red arrowheads) and upper dermal lymphocytic infiltrate (white arrowheads) (original objective magnification 10×) (**A**). Multiple cornoid lamellae in hypertrophic, digitate epidermis with vacuolised basal keratinocytes (yellow arrows) and thinning/focal absence of stratum granulosum (green arrowheads) (original objective magnification 20×) (**B**). Parakeratotic column (retention of keratinocyte nuclei), delved into the epidermis. Hypogranulosis with multiple dyskeratotic cells (blue arrow) extending from basal to spinous layer and single necrotic keratinocytes can be appreciated (blue arrowheads) (original objective magnification 20×) (**C**).

Confirmation with pathology may be recommended in atypical cases. Multiple, often concentrically arranged and large, cornoid lamellae, irregular or uniform papillomatosis (digitate epidermis) and/or psoriasiform epidermal hyperplasia and papillary dermis telangiectasia are the unique features of the disease, enabling reliable differentiation from PM (Figure 17) [465,469,476,477,480,483,485,487,503]. Some lesions may display folliculocentric or perieccrine arrangement (esp. in penoscrotal cases) [465,473]. It is speculated that multiple parakeratotic columns may be responsible for the thick and verrucous character of VP [465]. The disease features an absent or diminished granular layer [468,476,506] with multiple dyskeratotic cells present also in the spinous layer [495]. A vacuolated basal layer, individual necrotic keratinocytes, focal hyperchromatic cells, dermal inflammatory infiltrate and pigment incontinence can be observed [469,476,491,499,503]. Subepidermal amyloid deposits reported in a number of VP cases [47,471,494,507,508] might be associated with the concomitant chronic itching and subsequent rubbing in the intertriginous areas. As dyskeratotic epidermal cells and focal basal degeneration are more prominent under cornoid lamella, the occurrence of amyloid deposits might also be associated with epidermal defect [507]. Of note, some cases may lack amyloid [506], which might be attributed to the limited amount of friction in the particular non-intertriginous areas (e.g., the scrotal region) [471].

### 8.6. Treatment

Treatment of VP is very difficult and rarely leads to satisfying clearance of the lesions. Multiple case reports describe temporary improvements, yet ultimately a relapse.

Nine patients with limited lesions underwent surgical excision with a good to excellent effect [191,465,467,475,489,506,509,510]. One- to six-year follow-ups reported no recurrence in four cases [465,467,510] a complete relapse after 13 years in one case [506] and solitary recurrent lesions after 3 years in other [489]. Huang et al. reported a series of six patients with limited VP lesions successfully treated using CO_2_ lasers or surgery (no precise data) with no relapse at up to 6-year follow-up [469]. Dermabrasion was fully effective in one reported case (no relapse at 6-month follow-up) [6] and ineffective in another [475]. Curettage was used in one case with a good effect [495]. Unfortunately, the majority of reported cases lack data on long-term follow-up.

Cryotherapy completely cleared the lesions in four VP cases [345,416,511,512], demonstrated some/temporal effectiveness in four cases [191,290,465,471] and a poor outcome in three cases [465,507,513]. Considering the treated site, the main limitation for the procedure was pain [471].

Complete clearance with no relapse within 1–12 years was noted in three VP cases treated using a CO_2_ laser [467,514], whereas in nine, the lesions recurred after up to 13 years [6,191,290,465,467,474,475,507]. An excellent therapeutic outcome in six patients with localised VP lesions was achieved using CO_2_ lasers or surgery (no precise data) [469]. An excimer laser failed to provide a long-lasting effect in one VP case [475].

One case was subjected to destruction with a microwave knife (10 procedures) [60]. Complete clearance was achieved, with no recurrence during 6-year follow-up. Ultrasonic surgical aspiration was fully successful in one patient with vulvar VP and provided excellent cosmetic results [490].

PDT applied to three VP patients (unknown regimen and photosensitiser) were temporally effective (relapse was noted) in two and ineffective in one [496,497]. In two patients MAL-PDT (2–8 sessions) reduced the itch and provided partial resolution of the plaques [496]. 5-ALA-PDT was introduced in one patient with prior VP control with imiquimod 5% cream (3 h occlusion; light dose 37 J/cm; two sessions at a 4-week interval) achieving complete clearance of the remaining lesions and no recurrence at 52-week follow-up [466]. A lack of clinical response was observed during topical PUVA treatment in one VP patient [464]. Moreover, the intense burning sensation evoked by PUVA was a reason for treatment cessation.

Various topical glucocorticosteroids were applied in 24 reported cases and failed to reduce the lesions, apart from a temporal anti-pruritic effect noted in some patients [191,294,345,396,463,464,465,466,467,468,475,477,481,485,494,495,496,497,499,504,506,508,509,515]. Neither intralesional [495] or systemic glucocorticosteroids [481] provided any significant disease control. Topical calcineurin inhibitors, pimecrolimus [465,484] and tacrolimus [475], were used with poor effect.

Imiquimod 5% cream (three times per week; 8 weeks) led to full resolution in one VP patient [516], a good effect and itch control in one patient (daily application for 24 weeks) [466] and was ineffective in eight (1–12 weeks) [6,191,465,474,492,496,497,517]. Subsiding lesions produced residual hyperpigmentation [516]. A urticarial reaction, joint pain [465] or severe irritation [6] led to treatment discontinuation in two cases. In one case, topical administration of imiquimod 5% cream alternating with 5-FU achieved an anti-pruritic effect at week 8 [517].

Treatment with 5-FU 5% cream failed to improve the disease course in six VP cases [6,465,484,494,517,518], yet was able to achieve a temporal anti-pruritic effect in four of them. Partial VP remission was observed in one VP case at week 2, but the treatment was discontinued due to local irritation [519].

Topical vitamin D derivatives, i.e., tacalcitol [497], calcitriol [475,497], calcipotriol [464,497,518] and an unknown vitamin D analogue [191], provided no response in four reported VP cases.

Vitamin A derivates were used in VP in a number of cases. The responses to topical application were generally poor. Tretinoin 0.05% cream was reported to control recurrence in two cases [465,496], whereas tretinoin 0.025–0.075% cream [61,201,463,494,503,518,520], topical tazarotene 0.1% [191] topical retinoic acid [496,497] and unspecified retinoids [488,508,513,519] failed to achieve any durable effect, and in one case, were discontinued due to irritation [463]. Oral acitretin (25 mg daily [0.3 mg/kg] for 6 months, then tapered to 10 mg daily for additional 3 months) provided an excellent outcome in regard to lesion count, pruritus and irritation in one VP case [477], a moderate improvement in three cases (10–20 mg daily) [58,495,521] and a poor effect in two (30 mg daily for 2 months; and unknown regimen) [488,494]. Treatment failure with oral isotretinoin (unknown regimen) was reported in one case [475]. On the other hand, combined treatment with oral isotretinoin (25 mg/day; 4 weeks) and 5-FU 5% cream demonstrated moderate effectiveness in 4 weeks, yet the patient was lost to follow-up [506]. One case treated with isotretinoin (unknown dose; 6 months) and topical tazarotene 0.1% gel was reported to show a 25% reduction [468]; however, it remains unclear whether the authors addressed the VP severity or affected area. Oral isotretinoin (20 mg/day) combined with topical 5-FU in the morning and tretinoin 0.025% cream at night led to partial subsidence of lesions at week 4 [501]. One VP case treated with etretinate (regimen unknown) ceased treatment due to the intolerable side effects [294].

Out of four VP patients treated with diclofenac 3% gel, all failed to respond [191,496,497]. In one case, strong local irritation led to tapering the dose.

Other miscellaneous topical modalities used did not affect the disease course: topical antifungals (six patients) [345,466,499,501,509,515], glycolic acid [496,497], podophyllin [345,465], salicylic acid [467,497], urea (2 patients each) [474,503], topical antibiotics [509], coal tar formulations [464] and zinc oxide [497]. Systemic antifungals (three patients) [466,499,506], oral antihistamines [466], oral antibiotics (chlortetracycline and clindamycin) [475], antitubercular therapy [506] and intralesional administration of bleomycin [45] provided no clinical response.

The use of topical 3-hydroxy-3-methylglutaryl coenzyme A inhibitors (statins) aims at preventing the accumulation of toxic metabolites of the malfunctional mevalonate–isoprenoid pathway. A combined treatment with cholesterol 2% and simvastatin 1% ointment on petrolatum base was applied in two VP patients (twice per day, progressively tapered to 2–3 times per week) with a good safety profile and tolerability of the treatment [497]. The affected area was reduced by 50.0% (week 104, patient no. 1) and 66.7% (week 96, patient no. 2), with good control of itch, scaling and discomfort. In both cases, there was a notable decrease in the Dermatology Quality of Life Index (DLQI) (from 7 to 4 and from 11 to 5, respectively), whereas the satisfaction from the treatment was excellent. No relapse was observed in over a 2-year follow-up. In the authors’ experience, VP does not respond to monotherapy with cholesterol 2% ointment.

## 9. Follicular Porokeratosis (FP)

### 9.1. Clinical Presentation

The initial report on the involvement of adnexal structures (eccrine gland ostia and hair follicle infundibula) in porokeratosis dates back to 1980 [522]. Since that time, this phenomenon has been noted in many variants, e.g., PM, DSAP, DSP and porokeratotic eccrine and hair follicle nevus [408,523,524,525,526]. FP was formally acknowledged as a distinct variant by Pongpudpunth et al. in 2009 [526], yet the discussion on whether it is a separate condition or just a histological phenomenon potentially present in other variants is still ongoing [523].

FP presents with a wide range of clinical phenotypes [408,527,528]. Small (usually <1 cm in diameter), static, either itchy or asymptomatic, well-defined, erythematous, violet, pinkish, skin-coloured, brownish or dusky folliculocentric papules and/or nodules with a raised keratin rim may localise on sun-exposed and sun-unexposed sites (excluding the palms and soles) [241,525,526,527,529,530,531,532], and occasionally may be accompanied by follicular spicules [523,528]. Rare, limited FP cases with exclusive nasal skin [531,533,534,535,536] or scalp involvement [537] were also reported. The former seems to be more common in young adults and is speculated to be a distinct subvariant [531]. Examination with Wood’s lamp exhibits “diamond-necklace-like” lesions characterised by a bright white annular structure and multiple inner bright spots [529]. There was no sex predominance noted in the reported cases and the peak morbidity was noted in middle-aged individuals (range from 5 to 85 years) [525,529,530,537]. Speculatively, UV radiation might be involved in pathogenesis, as some cases displayed worsening with sun exposure or developed over sun-exposed sites [524,525,529,533,534]. In a patient with Sturge–Weber syndrome, FP caused secondary alopecia due to follicular hyperkeratosis and perifolliculitis [528]. Currently, no malignancy has been reported in FP.

Differential diagnoses for FP include a wide range of entities: nevus comedonicus, lichen planopilaris, lichen nitidus, lichen spinulosus, follicular cutaneous T-cell lymphoma, follicular porokeratoma (porokeratotic acanthoma), trichodysplasia spinulosa, hyperkertotic spicules associated with paraproteinemia, keratosis pilaris, phrynoderma and paraneoplastic spiny keratoderma [528,532]. Multiple minute digitate hyperkeratosis (filiform hyperkeratosis), presenting as hyperkeratotic spicules emerging from minute annular follicular and non-follicular plaques, is speculatively considered by some authors to belong to the same FP spectrum [538].

### 9.2. Genetics and Epigenetics

A genetic trait was proposed by Pongpudpunth et al. [526], and later on for two well-documented familial cases [524,529]. Until now, no culprit genes have been identified.

### 9.3. Dermatoscopy

Dermoscopy shows a distinct annular keratotic rim and atrophic centre. Several follicular plugs can be appreciated within the lesion [529]. In a case affecting the scalp, trichosopy showed poorly defined pinkish and violet areas with a prominent vascular network and yellow keratotic plugs in some follicles [539].

### 9.4. Confocal Microscopy

No data.

### 9.5. Pathology

Infundibulocentric parakeratotic columns with consumption of the underlying granular layer are recognised as histological hallmarks of the disease. Apoptotic basal keratinocytes (dyskeratosis) and focal interface lichenoid dermatitis can be observed [241,525,526,529,530,531,533,535,536,539]. In one case, a mild increase in dermal mucin deposition was noted [526]. Multiple minute digitate hyperkeratosis, speculated by some authors to be a FP subvariant, shares hypo-/agranulosis and a cornoid lamellation pattern with FP; however, no dysplasia, angularity of columns or keratinocyte vacuolisation are usually noted [538].

The suspected mechanisms by which folliculocentric cornoid lamella might develop include: (1) clonal proliferation of keratinocytes and their spread into the hair follicle [524] and (2) the proliferation of follicular stem cells [526,534]. Congenital or acquired mutation in the epidermal cells develops secondary to inflammatory responses [529].

Interestingly, some cases described as FP present with additional interfollicular cornoid lamellae [525]. A retrospective histopathological study on various types of porokeratosis, which included 86 cases from 73 patients, demonstrated a 14% incidence of infundibular/acrosyringial cornoid lamellae [408]. Thus, this histopathological feature might not be specific to FP, but present as a site-specific sign [408,523]. As the term “FP” is merely used by some authors to describe the distinctive histopathological phenomenon, it is unclear whether all the FP cases reported in the literature truly exhibit peculiar clinical characteristics and should be classified as FP (just as was the case with pruriginous follicular porokeratosis [241] that clinically matched the EDP variant of DSP).

### 9.6. Treatment

Various modalities were utilised in FP, yet all of them provided little to no effect. These included cryotherapy [529], topical steroids [526,533], topical pimecrolimus cream (twice per day; 2 months) [539], topical 5-FU (twice per day; 4 months) [530], imiquimod 5% cream (1 case treated once per day for 2 weeks) [532,533], cholesterol/lovastatin ointment (unknown concentration and regimen) [530], tretinoin cream [528] and ketoconazole shampoo [528]. No effect was achieved with systemic treatment with oral hydroxychloroquine (200 mg twice per day) [526] and acitretin (unknown regimen) [528]. Moreover, the latter led to the development of abscesses and spiculisation, aggravated xerosis and itch.

## 10. Porokeratoma

### 10.1. Clinical Presentation

Porokeratoma (porokeratotic acanthoma), introduced in a case series by Walsh et al. in 2007 [540], is an isolated well-demarcated porokeratotic plaque or nodule deprived of the typical annular rim of scale, sometimes verrucous, hyperkeratotic, or depressed [1,540,541,542], and predominantly involving the upper and lower extremities, followed by the buttocks, face and trunk [543]. Even though the majority of cases were solitary, multiple porokeratomas have also been reported [12,541,544,545]. The male to female ratio is 19:4, whereas the mean age of onset is 55 years (age range from 13 to 78) [543].

FP was reported in combination with PM [316] and DSP [12]. Nevertheless, individuals affected by porokeratoma usually have no familial/personal history of porokeratosis, drug triggers or immunosuppression [540,545]. The concomitance of porokeratoma and severe ankylosing spondylarthritis (two patients; one with paraplegia secondary to poliomyelitis) [541,545], Parkinson’s disease [316], chronic lymphocytic leukaemia [541] and non-HIV-related immunodeficiency [12] has been reported.

Porokeratoma is frequently misdiagnosed due to its substantial similarity to squamous cell carcinoma, basal cell carcinoma, actinic keratosis, psoriasis, seborrheic keratosis, papular amyloidosis (lichen amyloidosis), chromoblastomycosis, viral wart, prurigo nodularis, perforating dermatoses, digital mucous cyst and excoriation [541,543,545].

Although there have been no reports of malignant transformation in porokeratoma, the histopathological similarities between the subtypes could warrant a regular follow-up.

### 10.2. Genetics and Epigenetics

Even though some researchers claim that porokeratoma is sporadic [543], the presence of *PMVK* mutation in porokeratoma [540] was recently confirmed by Zhang et al. [15]. All porokeratomas studied in a genetic sequencing study on porokeratosis (5/134 index cases) demonstrated PMVK mutations (suggestively sporadic), confirming its clinical similarity to solitary VP and hyperkeratotic and classical PM [15]. Interestingly, according to the aforementioned study, *PMVK* mutation was a predisposing factor for genital localisation [15].

### 10.3. Dermatoscopy

There is only one case report which specifies the dermatoscopic features in porokeratoma: papillary protruding structures with little white–yellow scales, and dots and punctate vessels [12].

### 10.4. Confocal Microscopy

No data.

### 10.5. Pathology

Porokeratoma can be characterised as a localised form of porokeratosis, sharing the same histological aspect, namely the cornoid lamella. Of note, an abrupt transition to normal skin is prominent, and the lesion exhibits a distinct pattern of irregularly distributed cornoid lamellation throughout the area with multiple discrete or broad confluent columns of parakeratosis [540,545]. Follicular involvement (follicular porokeratoma), histologically resembling the FP variant, was reported in one case [543]. Contrary to the classic forms of porokeratosis, the central aspect of porokeratoma is non-atrophic but hyperplastic [540,543,545,546]. Compact orthokeratosis with infralamellar dyskeratosis, acanthosis and agranulosis can be seen, accompanied by a mildly dilated vascular plexus with mid-dermal predominantly lymphohistiocytic infiltrate and occasionally with dermal hemosiderin deposits [540,541,543,545,546,547]. Papillomatosis can be appreciated in some lesions [540]. In regard to dermatoscopy–pathology correlation, the papillary protruding structures with yellow–white scales and dots correspond to verrucous hyperplasia with multiple cornoid lamellae and keratin pearls, whereas the dotted vessels reflect dermal capillaries seen from above [12].

The lesion should be differentiated from granular parakeratotic acanthoma and hypergranulotic dyskeratotic acanthoma, with the former lacking cornoid lamellation, and the latter showing unique granular parakeratosis, either limited to the infundibula or extending to the interfollicular epidermis [543]. Contrarily, cornoid lamellae can be occasionally noted in prurigo nodularis, viral warts, seborrheic and solar keratosis, SCCs and BCCs, lichen planus and nevus sebaceous. Despite that, these lesions often present characteristic structural and cytological features, enabling their reliable differentiation [543].

Considering immunohistochemical staining, downregulated expression of keratinocyte maturation antigens at the cornoid lamella level underlines a disturbed epidermal differentiation in porokeratoma. In one reported case, several keratinocytes expressed Ki67 (proliferation marker), which was underexpressed at the level of the cornoid lamella. Beneath the cornoid lamella, there were relatively fewer CD1a+/CD207+ Langerhans cells. This area also displayed a decreased keratinocyte surface expression of Ulex Europaeus Agglutinin-I (UEA-I; pan-endothelial marker) lectin and ß-catenin, whereas the epidermal expression of E-cadherin (epithelial marker) and epidermal growth factor receptors (EGF-R; basal layer marker) were normal. Involucrin and filaggrin overexpression (confined to the most suprabasal layers) was noted, whereas the protein expression of p53 and p63 (tumour suppressors) were within normal limits. Occasional expression of epidermal membrane antigen (EMA) was observed underneath the cornoid lamella. Moreover, keratinocyte CD138/syndecan-1 (keratinocyte differentiation marker) was shown to be altered (not surface but cytoplasmic staining) [545].

HPV testing was negative in three cases [12,541,545] and positive in one (HPV 16) [544]. In one case, staining with gentian violet confirmed the presence of prominent amyloid deposits in the papillary dermis [548]. This phenomenon could have been evoked by the patient himself, who admitted to chronic scratching.

### 10.6. Treatment

Since porokeratoma is still a fairly new entity, a very few cases with a few treatment modalities have been published.

Surgical excision was adopted in 12 cases with excellent cosmetic effect and no relapse reported in 10 [540]. No follow-up data were available in two patients [545,548]. An excellent outcome was achieved with a shave biopsy followed by curettage in one case, with no relapse in 2-year follow-up [542].

Cryotherapy provided therapeutic success in one case [540] and failed in another [544].

Oral retinoids were administered only in single cases. Acitretin (30 mg daily; 6 months) alongside Helium-Neon 1064 nm laser therapy led to a good outcome (drop in lesion count with no recurrence), allowing the dose to be tapered to 25 mg/day [12]. The same retinoid (0.4–0.6 mg/kg/day) partially reduced the lesion in 3 months, but the patient was lost to follow-up [316].

Topical 5-FU 5% cream (twice daily; three courses: 2, 3, 2 weeks, each followed by a 2-week healing break) led to complete lesion resolution at week 13 in one case, with no relapse in 2.5-year follow-up [546]. The treatment was well tolerated, whereas post-inflammatory erythema and scaling gradually subsided with each treatment course.

Topical steroids [548] and oral antifungals provided no clinical effect [12].

## 11. Risk of Malignancy

**Figure 18:**
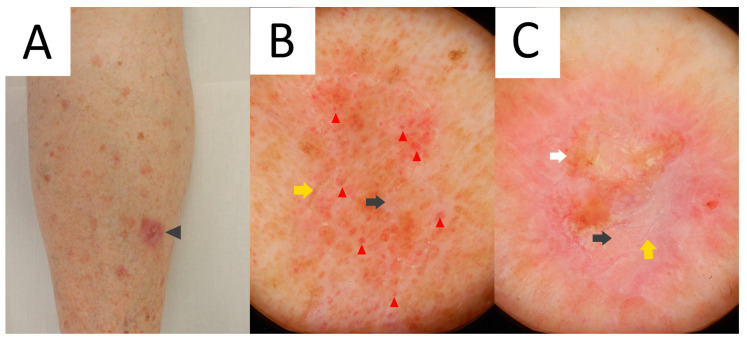
Cutaneous squamous cell carcinoma developing on the lower leg in elderly female with disseminated superficial actinic porokeratosis (black arrowhead) (**A**). Dermatoscopy reveals white continuous keratotic rim (cornoid lamella; yellow arrow) that limits homogeneous pinkish–tan area with linear glomerular vessels (black arrow and red arrowhead, respectively). This vascular pattern developing due to venous stasis is typical for lower legs (**B**). Dermatoscopy displays pink–whitish structureless area (black arrow) with a central keratin plug (white arrow) and vascular polymorphism suggestive of squamous cell carcinoma (magnification 20×) (**C**).

Porokeratosis is reportedly associated with an increased risk of malignancy, particularly keratinocyte cancer. This relation was noted first by Vigne in 1942 [549]. Although usually a solitary neoplasm develops within the lesion, multiple cancers in one patient were occasionally reported. SCC is the most commonly associated tumour in patients with porokeratoses (Figure 18) [18,282,550], yet a higher incidence of BCC and melanoma has also been demonstrated in this group [15,18,86,551,552,553,554], According to the literature, the estimated risk for developing keratinocyte cancer is 6.8–11.6% [555,556]. Malignant transformation occurs reportedly in 3.4% of DSAP, 7.6% of PM and 19% of LP [398]. Nevertheless, it is likely that the real-world risk is much lower. The most common porokeratosis subtypes and their potential for malignant transformation are summarised in Table 1.

High risk has been reportedly attributed to long-standing and large lesions, lesions located at acral and non-exposed sites, older age and personal history of irradiation [327,398,556,557]. Impaired immune surveillance can play a role in transplant patients, users of immunosuppressive drugs and individuals with AIDS [195,206,311,350,558].

The pathophysiology of carcinogenesis in porokeratosis is poorly understood. One of the theories presented for explaining this relation is a loss of heterozygosity as a first step towards neoplastic transformation [399]. The clonal proliferation of dysplastic keratinocytes in cornoid lamellae may be a consequence of intrinsic and extrinsic stimuli [282]. The former may include genetic factors (e.g., chromosome 3p.12–14 instability), with the latter including various factors impairing immune status (viz. immunosuppression, UV, infections, mechanical trauma and medications) [282,357]. Polyploidy is a situation where a cell features one or more extra pairs of chromosome copies. This phenomenon, common in SCC [559], has been demonstrated in porokeratosis [556,559,560], in particular in PM [561,562], and PPPD lesions [561,563], being a possible risk factor for cancerogenic potential.

Telomeres are vital protective elements of chromosomes. These regions of repetitive DNA sequences conserve the genome from nucleolytic degradation during chromosome replication and play an important role in ageing and cancer. Immortality, an uncontrollable self-replication, is a crucial feature of neoplasms. Human telomerase reverse transcriptase (hTERT) is responsible for aberrant cell proliferation, immortalisation and metastasis in cancers [564]. Interestingly, hTERT has been shown to be overexpressed in BCC, SCC, actinic keratosis and porokeratosis, and putatively involved in malignant transformation [565].

Oncogenesis in porokeratosis has been attributed to several signalling pathways controlling cell replication and apoptosis. p53 and p63 (p53 homolog) are tumour suppressor proteins, the most commonly overexpressed proteins in human neoplasms [565,566,567,568,569]. There are reports on p53 and p63 overexpression in various subtypes of porokeratosis [195,253,565,568,569,570,571]. p53 expression has been shown to be upregulated mainly in the inner aspect of the lesion, yet sub-lamellar and peripheral overexpression has been also noted in some cases [195,253,568,569]. Interestingly, p53 overexpression at the protein level was not matched with disrupted expression at the gene level, which questions the role of UV in porokeratosis [568,571]. Uncorrelated protein and gene expression does not seem to be uncommon in skin cancers [572] and may suggest epigenetic control of this process.

Cell cycle progression from the G0 or G1 to the S phase is regulated by cyclin-dependent kinases 4 and 6 [566,573]. p16 INK4a is a tumour suppressor inhibitor strongly linked to cell senescence, inhibiting cell cycle progression via downregulation of the activity of cyclin-dependent kinases 4 and 6 [566]. p16 INK4a was reported to be overexpressed at the mRNA level in one LP case [574] and at the protein level in the majority of DSP and PM cases evaluated [575]. Its expression under the cornoid lamella and within the central aspect of the lesion was speculatively associated with the focal senescence of keratinocytes and cornoid lamella formation [575]. Interestingly and counterintuitively, cell senescence and cancerogenesis seem to be closely related, as ageing cells may locally promote malignant transformation [573].

Survivin (baculoviral inhibitor of apoptosis repeat-containing 5, BARC5) is an apoptosis inhibitor overexpressed in a number of neoplasms [576], including SCC, yet not BCC [565]. Similar survivin expression patterns in the basal layer of porokeratosis and actinic keratosis support the premalignant character of both conditions [565].

## 12. Summary

Porokeratosis is a rare disease that is often misdiagnosed and mistreated, in some cases for years. A comprehensive knowledge on clinical presentation, dermatoscopy and treatment methods might be helpful to evaluate the lesions and eventually introduce an appropriate management. It should be underlined that patient education in regard to cancer awareness, self-examination and sun protection is pivotal for early detection and treatment in patients with porokeratoses. Regular long-term follow-up should be recommended in each patient. Despite no expert consensus existing, we suggest that yearly follow-up in patients with porokeratosis and no history of skin cancer could be reasonable.

## Figures and Tables

**Table 1:** Key clinical, epidemiological and pathological data on major variants of porokeratosis.

Clinical Variant	Onset	Gender	Key Clinical Features	Distribution	Key Pathological Features	Estimated Risk for Malignant Transformation
**DSAP**	3rd to 4th (familial cases) or 3rd to 5th decade (sporadic cases)	F > M	Multiple atrophic/keratotic macules and papules < 1 cm in size	Sun-exposed areas	Single >> doubled CL	3.4%
**DSP**	4th to 5th decade	F > M	Same as DSAP	Generalised	Same as DSAP	Low
**EDP**	6th to 7th decade	M > F	Self-limiting, extremely pruritic lesions; possible paraneoplastic character	Generalised	Same as DSAP, eosinophilic infiltrate	None (no data)
**PM**	Childhood (familial cases) or at any age (sporadic cases)	M > F	Solitary plaque with prominent border	Limbs	Single or multiple CLs; epidermal invagination and papillomatosis	7.6%
**LP**	Childhood	F ≥ M	Linear, verrucous plaques	Limbs (unilateral and blaschkoid)	Multiple CLs	11–19%
**PPPD**	Adolescence and 2nd decade	M > F	Multiple keratotic papules	Palms and soles (initially), limbs and trunk (later)	CL	Low
**PuP**	3rd to 5th decade	M > F	Multiple tender pits filled with keratotic plugs	Palms and soles (bilateral and linear or diffuse)	CL; keratin plugs	None(no data)
**VP**	3rd to 5th decade	M > F	Multiple, itching or burning, verrucous or psoriasiform patches	Anogenital region and skin folds (butterfly-shape appearance)	Multiple large CLs; papillomatosis and/or psoriasiform epidermal hyperplasia and papillary dermis telangiectasia	Low
**FP**	2nd to 6th decade	M > F	Folliculocentric papules and/or nodules < 1 cm in size	Any site (except palms and soles)	Infundibulocentric CL	None (no data)
**Porokeratoma**	5th decade	M > F	Isolated verrucous plaque of nodule	Limbs	Multiple discrete or broad confluent CLs; hypertrophic centre	None (no data)

**Legend:** CL—cornoid lamella, DSAP—disseminated superficial actinic porokeratosis, DSP—disseminated superficial porokeratosis, EDP—eruptive disseminated porokeratosis, F—female, FP—follicular porokeratosis, LP—linear porokeratosis, M—male, PPPD—porokeratosis palmaris, plantaris et disseminata, PuP—punctate porokeratosis, VP—verrucous porokeratosis.

## References

[1] Patterson J.W. (2015). Weedon’s Skin Pathology.

[2] Neumann I. (1875). Uber Eine Noch Wenig Bekannte Hautkrankheit (Dermatitis Circumscripta Herpetiformis). Vierteljahrsschr. Dermatol. Syph. Wien..

[3] Respighi E. (1893). Di Une Ipercheratosi Non Ancora Descritta. G. Ital. Dermatol. Venereol..

[4] Mibelli V. (1899). Ueber Einen Fall von Porokeratosis Mit Localisation im Munde und an der Glans. Arch. Dermat. Syph..

[5] Freyschmidt-Paul P., Hoffmann R., König A., Happle R. (1999). Linear Porokeratosis Superimposed on Disseminated Superficial Actinic Porokeratosis: Report of Two Cases Exemplifying the Concept of Type 2 Segmental Manifestation of Autosomal Dominant Skin Disorders. J. Am. Acad. Dermatol..

[6] Wallner J.S., Fitzpatrick J.E., Brice S.L. (2003). Verrucous Porokeratosis of Mibelli on the Buttocks Mimicking Psoriasis. Cutis.

[7] Mukhopadhyay A.K. (2004). Simultaneous Occurrence of Disseminated Superficial, Linear and Hypertrophic Verrucous Forms of Porokeratosis in a Child. Indian J. Dermatol. Venereol. Leprol..

[8] Niimi Y., Kawana S. (2009). Type 2 Segmental Manifestation of Disseminated Superficial Actinic Porokeratosis in a 7-Year-Old Girl. Eur. J. Dermatol..

[9] Murase J., Gilliam A.C. (2010). Disseminated Superficial Actinic Porokeratosis Co-Existing with Linear and Verrucous Porokeratosis in an Elderly Woman: Update on the Genetics and Clinical Expression of Porokeratosis. J. Am. Acad. Dermatol..

[10] Koley S., Sarkar J., Choudhary S., Dhara S., Choudhury M., Bhattacharya S. (2011). Different Morphological Variants of Hypertrophic Porokeratosis and Disseminated Lesions of Porokeratosis of Mibelli: A Rare Co-Existence. Indian J. Dermatol. Venereol. Leprol..

[11] Guo H., Gao X.-H., Chen H.-D., Li J.-H. (2015). Coexistence of Multiple Variants of Porokeratosis. Indian J. Dermatol. Venereol. Leprol..

[12] Xu X., Pradhan S., Wang D., Li W. (2020). Multiple Porokeratomas (Porokeratotic Acanthoma) Coexisting with Disseminated Superficial Porokeratosis: Clinical, Dermoscopic and Pathological Observations, and Review of Published Work. J. Dermatol..

[13] Sotoodian B., Mahmood M.N., Salopek T.G. (2018). Clinical and Dermoscopic Features of Pigmented Disseminated Superficial Actinic Porokeratosis: Case Report and Literature Review. J. Cutan. Med. Surg..

[14] Zaar O., Polesie S., Navarrete-Dechent C., Errichetti E., Akay B.N., Jaimes J., Cabo H., Cohen Sabban E., Paoli J. (2021). Dermoscopy of Porokeratosis: Results from a Multicentre Study of the International Dermoscopy Society. J. Eur. Acad. Dermatol. Venereol..

[15] Zhang Z., Li C., Wu F., Ma R., Luan J., Yang F., Liu W., Wang L., Zhang S., Liu Y. (2015). Genomic Variations of the Mevalonate Pathway in Porokeratosis. eLife.

[16] Chernosky M.E., Freeman R.G. (1967). Disseminated Superficial Actinic Porokeratosis (DSAP). Arch. Dermatol..

[17] Kong F., Moreira-Lucas T.S., Kaminski A., Spelman L. (2021). Management of Disseminated Superficial Actinic Porokeratosis and Intraepidermal Squamous Cell Carcinoma with Low-Dose Radiation Therapy. Australas. J. Dermatol..

[18] Inci R., Zagoras T., Kantere D., Holmström P., Gillstedt M., Polesie S., Peltonen S. (2023). Porokeratosis Is One of the Most Common Genodermatoses and Is Associated with an Increased Risk of Keratinocyte Cancer and Melanoma. J. Eur. Acad. Dermatol. Venereol..

[19] Waqar M.U., Cohen P.R., Fratila S. (2022). Disseminated Superficial Actinic Porokeratosis (DSAP): A Case Report Highlighting the Clinical, Dermatoscopic, and Pathology Features of the Condition. Cureus.

[20] Redondo P., Sola M.A., Lloret P. (2002). Porokeratosis and Povidone-Iodine: A New Clinical Diagnostic Sign. Br. J. Dermatol..

[21] Katugampola R.P., Finlay A.Y. (2006). Fake Sun Tan Diagnosis of Porokeratosis. J. Eur. Acad. Dermatol. Venereol..

[22] Thomas C.J., Elston D.M. (2005). Medical Pearl: Gentian Violet to Highlight the Cornoid Lamella in Disseminated Superficial Actinic Porokeratosis. J. Am. Acad. Dermatol..

[23] Navarrete-Dechent C., Uribe P., Marghoob A. (2019). Ink-Enhanced Dermoscopy Is a Useful Tool to Differentiate Acquired Solitary Plaque Porokeratosis from Other Scaly Lesions. J. Am. Acad. Dermatol..

[24] Le C., Bedocs P.M. (2023). Disseminated Superficial Actinic Porokeratosis. StatPearls.

[25] Gu C.-Y., Zhang C.-F., Chen L.-J., Xiang L.-H., Zheng Z.-Z. (2014). Clinical Analysis and Etiology of Porokeratosis. Exp. Ther. Med..

[26] Allen A.L., Glaser D.A. (2000). Disseminated Superficial Actinic Porokeratosis Associated with Topical PUVA. J. Am. Acad. Dermatol..

[27] Kawara S., Oiso N., Kawada A. (2011). Disseminated Superficial Actinic Porokeratosis in a Patient Undergoing Treatment with Long-Term Narrowband Ultraviolet B for Psoriasis. J. Dermatol..

[28] Ellis M., Fidai C., Kerr H. (2019). A Rare Case of Bullous Eruptive Disseminated Porokeratosis. Case Reports. https://scholarlycommons.henryford.com/merf2019caserpt/124.

[29] Kang B.D., Kye Y.C., Kim S.N. (2001). Disseminated Superficial Actinic Porokeratosis with Both Typical and Prurigo Nodularis-like Lesions. J. Dermatol..

[30] Bencini P.L., Tarantino A., Grimalt R., Ponticelli C., Caputo R. (1995). Porokeratosis and Immunosuppression. Br. J. Dermatol..

[31] Fields L.L., White C.R., Maziarz R.T. (1995). Rapid Development of Disseminated Superficial Porokeratosis after Transplant Induction Therapy. Bone Marrow Transpl..

[32] Matsushita S., Kanekura T., Kanzaki T. (1997). A Case of Disseminated Superficial Actinic Porokeratosis Subsequent to Renal Transplantation. J. Dermatol..

[33] Jang Y.-H., Chun S.-J., Kang W.H., Lee E.-S. (2004). Eruptive Disseminated Superficial Actinic Porokeratosis in an Immunocompetent Host: Is This Associated with Herpes Simplex Virus or Bacterial Infection?. J. Am. Acad. Dermatol..

[34] di Meo N., Fluehler C., Perkan V., Trevisan G. (2010). Disseminated Superficial Porokeratosis and Pyoderma Gangrenosum. Dermatol. Online J..

[35] Khaled A., Kourda M., Abdelmoula F., M’ssedi L., Tougourti M.N., Kamoun M.R. (2011). Late-Onset Disseminated Superficial Actinic Porokeratosis in an Elderly Woman. Dermatol. Ther..

[36] Sim C.Y., Shin J.Y., Lee S.Y., Park Y.L. (2018). Disseminated Superficial Actinic Porokeratosis in a Patient with Psoriasis, after Long-Term Narrowband Ultraviolet B Phototherapy. Ann. Dermatol..

[37] Breton A.L., Pralong P., Trillet-Lenoir V., Balme B., Nicolas J.-F., Berard F. (2014). Disseminated Porokeratosis Transiently Healed by Cancer Chemotherapy. Eur. J. Dermatol..

[38] Torchia D., Romanelli P., Schachner L.A. (2011). Disseminated Superficial Actinic Porokeratosis Associated with Pseudoxanthoma Elasticum. Eur. J. Dermatol..

[39] Monteagudo-Sánchez B., Ginarte M., Durana C., Labandeira J., de las Heras C., Cacharrón J.M. (2006). Porokeratosis in a patient with dermatomyositis. Actas Dermosifiliogr..

[40] Kluger N., Guilpain P., Leclerc-Mercier S., Emilie S., Guillevin L., Mouthon L. (2009). Superficial porokeratosis of the lower limbs during systemic scleroderma. Presse Med..

[41] Terranova M., Amato L., Massi D., Fabbri P. (2003). Disseminated Superficial Actinic Porokeratosis in a Patient with Sjögren Syndrome. Skinmed.

[42] Yao Z.-G., Hua F., Yin Z.-H., Xue Y.-J., Hou Y.-H., Nie Y.-C., Zheng Z.-M., Zhao M.-Q., Guo X.-H., Ma C. (2023). Characteristics of Glioblastomas and Immune Microenvironment in a Chinese Family with Lynch Syndrome and Concurrent Porokeratosis. Front. Oncol..

[43] Kanitakis J., Arbona-Vidal E., Faure M. (2012). Porokeratosis in Patients with Polycythemia Rubra Vera: A New Side Effect of Hydroxyurea?. J. Eur. Acad. Dermatol. Venereol..

[44] Romagnuolo M., Riva D., Alberti Violetti S., Di Benedetto A., Barberi F., Moltrasio C. (2023). Disseminated Superficial Actinic Porokeratosis Following Hydroxyurea Treatment: A Case Report. Australas. J. Dermatol..

[45] Suárez-Amor O., Pereiro-Ferreirós M., Ginarte M., Peteiro C., Toribio J. (2007). Coexistence of Linear Porokeratosis and Disseminated Superficial Actinic Porokeratosis: A Type 2 Segmental Manifestation. Acta Derm. Venereol..

[46] Gautam R.K., Bedi G.K., Schgal V.N., Singh N. (1995). Simultaneous Occurrence of Disseminated Superficial Actinic Porokeratosis (DSAP), Linear, and Punctate Porokeratosis. Int. J. Dermatol..

[47] Welton W.A. (1972). Linear Porokeratosis in a Family with DSAP. Arch. Dermatol..

[48] Dover J.S., Miller J.A., Levene G.M. (1986). Linear Porokeratosis of Mibelli and DSAP. Clin. Exp. Dermatol..

[49] Löhrer R., Neumann-Acikel A., Eming R., Hartmann K., Rasokat H., Krieg T., Happle R., Eming S. (2010). A Case of Linear Porokeratosis Superimposed on Disseminated Superficial Actinic Porokeratosis. Case Rep. Dermatol..

[50] Feldman S.R., Crosby D.L., Tomsick R.S. (1991). Scaly Atrophic Lesions Both Scattered and in Linear Arrays. Disseminated Superficial Actinic Porokeratosis in a Patient with Linear Porokeratosis. Arch. Dermatol..

[51] Pearson I.C., Cliff S. (2003). Case 6: Plaques Extending in a Linear Pattern from Left Ankle to Hip Forming over a 2-Year Period. Diagnosis: Linear Porokeratosis with Disseminated Superficial Porokeratosis Erupting in Pregnancy. Clin. Exp. Dermatol..

[52] Happle R. (1991). Somatic Recombination May Explain Linear Porokeratosis Associated with Disseminated Superficial Actinic Porokeratosis. Am. J. Med. Genet..

[53] Suh D.H., Lee H.S., Kim S.D., Cho K.H., Kim K.H., Park K.C. (2000). Coexistence of Disseminated Superficial Porokeratosis in Childhood with Congenital Linear Porokeratosis. Pediatr. Dermatol..

[54] Boente M.d.C., López-Baró A.M., Frontini M.d.V., Asial R.A. (2003). Linear Porokeratosis Associated with Disseminated Superficial Actinic Porokeratosis: A New Example of Type II Segmental Involvement. Pediatr. Dermatol..

[55] Cho E., Lee Y.B., Park H.J., Cho B.K. (2012). Coexistence of Congenital Linear Porokeratosis and Disseminated Superficial Porokeratosis. Australas. J. Dermatol..

[56] Commens C.A., Shumack S.P. (1987). Linear Porokeratosis in Two Families with Disseminated Superficial Actinic Porokeratosis. Pediatr. Dermatol..

[57] Shiiya C., Aoki S., Nakabayashi K., Hata K., Amagai M., Kubo A. (2021). Linear and Disseminated Porokeratosis in One Family Showing Identical and Independent Second Hits in MVD among Skin Lesions, Respectively: A Proof-of-Concept Study. Br. J. Dermatol..

[58] Peng J.-M., Xiao X.-M., Chen J.-W., Chen L.-F., Cheng B., Ji M.-K., Zhang Z.-H. (2021). Novel Mutation in MVK Gene for Co-Occurrence of Disseminated Superficial Actinic Porokeratosis with Porokeratosis Ptychotropica. J. Dermatol..

[59] Mei Q., Xing F., Yin Y., Yuan C. (2023). Case Report: A Novel MVK Missense Mutation in the Sporadic Porokeratosis Ptychotropica in China. Clin. Cosmet. Investig. Dermatol..

[60] Xu H.-J., Wen G.-D. (2022). Mixed Porokeratosis with a Novel Mevalonate Kinase Gene Mutation: A Case Report. World J. Clin. Cases.

[61] McGuigan K., Shurman D., Campanelli C., Lee J.B. (2009). Porokeratosis Ptychotropica: A Clinically Distinct Variant of Porokeratosis. J. Am. Acad. Dermatol..

[62] Mehta V., Balachandran C. (2009). Simultaneous Co-Occurrence of Porokeratosis of Mibelli with Disseminated Superficial Actinic Porokeratosis. Indian J. Dermatol..

[63] Kubo A., Sasaki T., Suzuki H., Shiohama A., Aoki S., Sato S., Fujita H., Ono N., Umegaki-Arao N., Kawai T. (2019). Clonal Expansion of Second-Hit Cells with Somatic Recombinations or C>T Transitions Form Porokeratosis in MVD or MVK Mutant Heterozygotes. J. Investig. Dermatol..

[64] Ng D., Brand R. (2019). A Precancerous Skin Lesion That Is Often Misdiagnosed. Aust. J. Gen. Pract..

[65] Zaballos P., Puig S., Malvehy J. (2004). Dermoscopy of Disseminated Superficial Actinic Porokeratosis. Arch. Dermatol..

[66] Delfino M., Argenziano G., Nino M. (2004). Dermoscopy for the Diagnosis of Porokeratosis. J. Eur. Acad. Dermatol. Venereol..

[67] Chi C., Liu J. (2017). Image Gallery: Porokeratosis under the Dermoscopic Furrow Ink Test and Ultraviolet Light. Br. J. Dermatol..

[68] Vargas-Mora P., Morgado-Carrasco D., Fustà-Novell X. (2020). Porokeratosis: A Review of Its Pathophysiology, Clinical Manifestations, Diagnosis, and Treatment. Actas Dermosifiliogr..

[69] Nicola A., Magliano J. (2017). Dermoscopy of Disseminated Superficial Actinic Porokeratosis. Actas Dermosifiliogr..

[70] Errichetti E., Zalaudek I., Kittler H., Apalla Z., Argenziano G., Bakos R., Blum A., Braun R.P., Ioannides D., Lacarrubba F. (2020). Standardization of Dermoscopic Terminology and Basic Dermoscopic Parameters to Evaluate in General Dermatology (Non-Neoplastic Dermatoses): An Expert Consensus on Behalf of the International Dermoscopy Society. Br. J. Dermatol..

[71] Hung R., Ahmeen M., Fleming A., Hoque S. (2013). Itchy Lesions in Pigmented Skin. BMJ Case Rep..

[72] Tan T.S.P., Tallon B. (2016). Pigmented Porokeratosis. A Further Variant?. Am. J. Dermatopathol..

[73] Reyna-Rodríguez I.L., García-Lozano J.A., Ocampo-Candiani J. (2021). Pigmented Disseminated Superficial Actinic Porokeratosis in Dark-Skinned Patients: Clinical, Dermoscopic, and Histopathologic Features. J. Cosmet. Dermatol..

[74] Mazzeo M., Longo C., Manfreda V., Piana S., Bianchi L., Pellacani G., Pampena R. (2020). Looking Horizontally at Disseminated Superficial Actinic Porokeratosis: Correlations between in-Vivo Reflectance Confocal Microscopy and Histopathology. Skin. Res. Technol..

[75] Broggi G., Verzì A.E., Caltabiano R., Micali G., Lacarrubba F. (2021). Correlation between In Vivo Reflectance Confocal Microscopy and Horizontal Histopathology in Skin Cancer: A Review. Front. Oncol..

[76] Moscarella E., Longo C., Zalaudek I., Argenziano G., Piana S., Lallas A. (2013). Dermoscopy and Confocal Microscopy Clues in the Diagnosis of Psoriasis and Porokeratosis. J. Am. Acad. Dermatol..

[77] Ulrich M., Forschner T., Röwert-Huber J., González S., Stockfleth E., Sterry W., Astner S. (2007). Differentiation between Actinic Keratoses and Disseminated Superficial Actinic Porokeratoses with Reflectance Confocal Microscopy. Br. J. Dermatol..

[78] Sertznig P., von Felbert V., Megahed M. (2012). Porokeratosis: Present Concepts. J. Eur. Acad. Dermatol. Venereol..

[79] Shen C.-S., Tabata K., Matsuki M., Goto T., Yokochi T., Yamanishi K. (2002). Premature Apoptosis of Keratinocytes and the Dysregulation of Keratinization in Porokeratosis. Br. J. Dermatol..

[80] Fernandez-Flores A. (2008). Small Lesions of Porokeratosis Show a Normal Proliferation Rate with MIB-1. Acta Dermatovenerol. Alp. Pannonica Adriat..

[81] Champagne C., Moore L., Reule R., Dyer J.A., Rady P., Tyring S.K., North J.P. (2015). Cornoid Lamella-Like Structures in HIV-Associated Epidermodysplasia Verruciformis: A Unique Histopathologic Finding. Am. J. Dermatopathol..

[82] Foster C., Tallon B. (2022). Porokeratosis: A Differential Diagnosis to Consider in Benign Lichenoid Keratosis. Int. J. Clin. Exp. Pathol..

[83] Maruyama T., Ito M. (1987). Disseminated superficial actinic porokeratosis with amyloid deposition. Nihon Hifuka Gakkai Zasshi.

[84] Yamamoto T., Furukawa H., Ohtsuka M. (2013). Amyloid Deposition in Disseminated Superficial Porokeratosis with Inflammatory Stages. J. Dermatol..

[85] Zhao Q., Yu B., Zhou H., Feng C., Zhang X., Zheng Y., Geng S. (2020). Generalized Type 2 Segmental Disseminated Superficial Actinic Porokeratosis Coexisted with Multiple Cutaneous Squamous Cell Carcinomas: Analysis of Two Cases. Indian. J. Pathol. Microbiol..

[86] Al-Haseni A., Chitgopeker P., Ho J.D., Goldberg L.J., Sahni D. (2018). Amelanotic Melanoma Arising within a Lesion of Disseminated Superficial Actinic Porokeratosis: An Unusual Presentation Leading to a Novel Therapeutic Approach. Dermatol. Ther..

[87] Happle R. (2010). Mibelli Revisited: A Case of Type 2 Segmental Porokeratosis from 1893. J. Am. Acad. Dermatol..

[88] Xia J.H., Yang Y.F., Deng H., Tang B.S., Tang D.S., He Y.G., Xia K., Chen S.X., Li Y.X., Pan Q. (2000). Identification of a Locus for Disseminated Superficial Actinic Porokeratosis at Chromosome 12q23.2-24.1. J. Investig. Dermatol..

[89] Zhang Z.H., Niu Z.M., Yuan W.T., Zhao J.J., Jiang F.X., Zhang J., Chai B., Cui F., Chen W., Lian C.H. (2005). A Mutation in SART3 Gene in a Chinese Pedigree with Disseminated Superficial Actinic Porokeratosis. Br. J. Dermatol..

[90] Xia K., Deng H., Xia J.H., Zheng D., Zhang H.L., Lu C.Y., Li C.Q., Pan Q., Dai H.P., Yang Y.F. (2002). A Novel Locus (DSAP2) for Disseminated Superficial Actinic Porokeratosis Maps to Chromosome 15q25.1-26.1. Br. J. Dermatol..

[91] Zhang Z., Niu Z., Yuan W., Liu W., Xiang L., Zhang J., Chu X., Zhao J., Jiang F., Chai B. (2004). Fine Mapping and Identification of a Candidate Gene SSH1 in Disseminated Superficial Actinic Porokeratosis. Hum. Mutat..

[92] Zhang Z.-H., Huang W., Niu Z.-M., Liu W.-D., Xiang L.-H., Yuan W.-T., Zhao J.-J., Gu C.-Y., Chai B., Jiang F.-X. (2005). Two Closely Linked Variations in Actin Cytoskeleton Pathway in a Chinese Pedigree with Disseminated Superficial Actinic Porokeratosis. J. Am. Acad. Dermatol..

[93] Liu P., Zhang S., Yao Q., Liu X., Wang X., Huang C., Huang X., Wang P., Yuan M., Liu J.Y. (2008). Identification of a Genetic Locus for Autosomal Dominant Disseminated Superficial Actinic Porokeratosis on Chromosome 1p31.3-P31.1. Hum. Genet..

[94] Luan J., Niu Z., Zhang J., Crosby M.E., Zhang Z., Chu X., Wang Z., Huang W., Xiang L., Zheng Z. (2011). A Novel Locus for Disseminated Superficial Actinic Porokeratosis Maps to Chromosome 16q24.1-24.3. Hum. Genet..

[95] Qian W., Wu J., Tang H., Zhen Q., Ge H., Gao J., Chen J., Chang Y., Wang W., Sun L. (2021). Mutation Analysis of the MVD Gene in a Chinese Family with Disseminated Superficial Actinic Porokeratosis and a Chinese Literature Review. Indian J. Dermatol..

[96] Zhu T., Tian D., Zhang L., Xu X., Xia K., Hu Z., Xiong Z., Tan J. (2019). Novel Mutations in Mevalonate Kinase Cause Disseminated Superficial Actinic Porokeratosis. Br. J. Dermatol..

[97] Brennenstuhl H., Nashawi M., Schröter J., Baronio F., Beedgen L., Gleich F., Jeltsch K., von Landenberg C., Martini S., Simon A. (2021). Phenotypic Diversity, Disease Progression, and Pathogenicity of MVK Missense Variants in Mevalonic Aciduria. J. Inherit. Metab. Dis..

[98] Miziorko H.M. (2011). Enzymes of the Mevalonate Pathway of Isoprenoid Biosynthesis. Arch. Biochem. Biophys..

[99] Zhang S.-Q., Jiang T., Li M., Zhang X., Ren Y.-Q., Wei S.-C., Sun L.-D., Cheng H., Li Y., Yin X.-Y. (2012). Exome Sequencing Identifies MVK Mutations in Disseminated Superficial Actinic Porokeratosis. Nat. Genet..

[100] Vlachou C., Kanelleas A.I., Martin-Clavijo A., Berth-Jones J. (2008). Treatment of Disseminated Superficial Actinic Porokeratosis with Topical Diclofenac Gel: A Case Series. J. Eur. Acad. Dermatol. Venereol..

[101] Buhaescu I., Izzedine H. (2007). Mevalonate Pathway: A Review of Clinical and Therapeutical Implications. Clin. Biochem..

[102] Cui H., Li L., Wang W., Shen J., Yue Z., Zheng X., Zuo X., Liang B., Gao M., Fan X. (2014). Exome Sequencing Identifies SLC17A9 Pathogenic Gene in Two Chinese Pedigrees with Disseminated Superficial Actinic Porokeratosis. J. Med. Genet..

[103] Kim H., Lee B.H., Do H.-S., Kim G.-H., Kang S., Koh K.-N., Im H.J. (2021). Case Report: Mevalonic Aciduria Complicated by Acute Myeloid Leukemia After Hematopoietic Stem Cell Transplantation. Front. Immunol..

[104] Yıldız Ç., Gezgin Yıldırım D., Inci A., Tümer L., Cengiz Ergin F.B., Sunar Yayla E.N.S., Esmeray Şenol P., Karaçayır N., Eğritaş Gürkan Ö., Okur I. (2023). A Possibly New Autoinflammatory Disease Due to Compound Heterozygous Phosphomevalonate Kinase Gene Mutation. Jt. Bone Spine.

[105] Lang B.M., Peveling-Oberhag A., Zimmer S., Wegner J., Sohn A., Grabbe S., Staubach P. (2020). Effective Treatment of Disseminated Superficial Actinic Porokeratosis with Chemical Peels—Customary Treatment for a Rare Disease. J. Dermatol. Treat..

[106] Li M., Min W., Wang J., Wang L., Li Y., Zhou N., Yang Z., Qian Q. (2020). Effects of Mevalonate Kinase Interference on Cell Differentiation, Apoptosis, Prenylation and Geranylgeranylation of Human Keratinocytes Are Attenuated by Farnesyl Pyrophosphate or Geranylgeranyl Pyrophosphate. Exp. Ther. Med..

[107] Politiek F.A., Waterham H.R. (2021). Compromised Protein Prenylation as Pathogenic Mechanism in Mevalonate Kinase Deficiency. Front. Immunol..

[108] Biswas A. (2015). Cornoid Lamellation Revisited: Apropos of Porokeratosis with Emphasis on Unusual Clinicopathological Variants. Am. J. Dermatopathol..

[109] Huang M., Zhou B., Gong J., Xing L., Ma X., Wang F., Wu W., Shen H., Sun C., Zhu X. (2018). RNA-Splicing Factor SART3 Regulates Translesion DNA Synthesis. Nucleic Acids Res..

[110] Timani K.A., Rezaei S., Whitmill A., Liu Y., He J.J. (2022). Tip110/SART3-Mediated Regulation of NF-κB Activity by Targeting IκBα Stability Through USP15. Front. Oncol..

[111] Kurita S., Gunji E., Ohashi K., Mizuno K. (2007). Actin Filaments-Stabilizing and -Bundling Activities of Cofilin-Phosphatase Slingshot-1. Genes Cells.

[112] Welch M.D., DePace A.H., Verma S., Iwamatsu A., Mitchison T.J. (1997). The Human Arp2/3 Complex Is Composed of Evolutionarily Conserved Subunits and Is Localized to Cellular Regions of Dynamic Actin Filament Assembly. J. Cell Biol..

[113] Schrank B.R., Aparicio T., Li Y., Chang W., Chait B.T., Gundersen G.G., Gottesman M.E., Gautier J. (2018). Nuclear ARP2/3 Drives DNA Break Clustering for Homology-Directed Repair. Nature.

[114] Atzmony L., Choate K.A. (2019). Second-Hit Somatic Mutations in Mevalonate Pathway Genes Underlie Porokeratosis. J. Investig. Dermatol..

[115] Atzmony L., Lim Y.H., Hamilton C., Leventhal J.S., Wagner A., Paller A.S., Choate K.A. (2020). Topical Cholesterol/Lovastatin for the Treatment of Porokeratosis: A Pathogenesis-Directed Therapy. J. Am. Acad. Dermatol..

[116] Ugwu N., Choate K.A., Atzmony L. (2020). Two Percent Lovastatin Ointment as a Pathogenesis-Directed Monotherapy for Porokeratosis. JAAD Case Rep..

[117] Marks S., Varma R., Cantrell W., Chen S.C., Gold M., Muellenhoff M., Elewski B. (2009). Diclofenac Sodium 3% Gel as a Potential Treatment for Disseminated Superficial Actinic Porokeratosis. J. Eur. Acad. Dermatol. Venereol..

[118] Darr-Foit S., Elsner P. (2018). 77-year-old female with persisting erythematous and scaly plaques on the extremities and upper trunk: Preparation for the medical specialist examination: Part 20. Hautarzt.

[119] Nayeemuddin F.A., Wong M., Yell J., Rhodes L.E. (2002). Topical Photodynamic Therapy in Disseminated Superficial Actinic Porokeratosis. Clin. Exp. Dermatol..

[120] Cavicchini S., Tourlaki A. (2006). Successful Treatment of Disseminated Superficial Actinic Porokeratosis with Methyl Aminolevulinate-Photodynamic Therapy. J. Dermatol. Treat..

[121] Aird G.A., Sitenga J.L., Nguyen A.H., Vaudreuil A., Huerter C.J. (2017). Light and Laser Treatment Modalities for Disseminated Superficial Actinic Porokeratosis: A Systematic Review. Lasers Med. Sci..

[122] Calzavara-Pinton P.G., Rossi M.T., Aronson E., Sala R., Italian Group for Photodynamic Therapy (2013). A Retrospective Analysis of Real-Life Practice of off-Label Photodynamic Therapy Using Methyl Aminolevulinate (MAL-PDT) in 20 Italian Dermatology Departments. Part 1: Inflammatory and Aesthetic Indications. Photochem. Photobiol. Sci..

[123] Salas T., Hernandez-Gil J., Lopez A., Dorado M., Ruiz J., García E., Martinez F. (2016). Two Cases of Disseminated Superficial Actinic Porokeratosis Treated with Daylight-Mediated Photodynamic Therapy. Dermatol. Ther..

[124] Ferrer Guillén B., Giácaman M.M., Pérez Ferriols A. (2018). Improved Effect on 2 Cases of Disseminated Superficial Actinic Porokeratosis with Daylight Photodynamic Therapy. Photodiagnosis Photodyn. Ther..

[125] Fernández-Guarino M., Harto A., Pérez-Garcia B., Martin-González M., Urrutia S., Jaén P. (2009). Photodynamic Therapy in Disseminated Superficial Actinic Porokeratosis. J. Eur. Acad. Dermatol. Venereol..

[126] Schmook T., Kraft J., Ulrich C., Stockfleth E. (2005). Disseminated Superficial Actinic Porokeratosis: Report of 7 Patients Successfully Treated with Cryotherapy. J. Am. Acad. Dermatol..

[127] Boiy A., de Witte P.A.M., Roelandts R. (2010). Topical Treatment of Disseminated Superficial Actinic Porokeratosis with Hypericin-Photodynamic Therapy: A Case Report. Photodiagnosis Photodyn. Ther..

[128] Ramelyte E., Bylaite-Bucinskiene M., Dummer R., Imhof L. (2017). Successful Use of Grenz Rays for Disseminated Superficial Actinic Porokeratosis: Report of 8 Cases. Dermatology.

[129] Ting S., Webster M. (2022). Grenz Ray Therapy in Disseminated Superficial Actinic Porokeratosis: A Case Series of 17 Patients. Australas. J. Dermatol..

[130] O’Reilly M., Butt S., Gajebasia S., Dawe R. (2022). PD13: Successful Treatment of Disseminated Superficial Actinic Porokeratosis with Grenz Ray Therapy. Br. J. Dermatol..

[131] Smith J., Narla S., Lyons A.B., Kohli I., Siddiqui F., Rao B.K., Penman L., Hamzavi I.H. (2020). Brachytherapy for Resistant Disseminated Superficial Actinic Porokeratosis. Appl. Rad. Oncol..

[132] Spelman L., Christie D., Kaminski A., Baker C., Supranowicz M., Sinclair R. (2022). Radiotherapy, Utilizing Volumetric Modulated Arc Therapy, for Extensive Skin Field Cancerization: A Retrospective Case Series Assessing Efficacy, Safety, and Cosmetic Outcomes at 12 Months after Treatment. Case Rep. Dermatol..

[133] Harrison P.V., Stollery N. (1994). Disseminated Superficial Actinic Porokeratosis Responding to Calcipotriol. Clin. Exp. Dermatol..

[134] Bakardzhiev I., Kavaklieva S., Pehlivanov G. (2012). Successful Treatment of Disseminated Superficial Actinic Porokeratosis with Calcipotriol. Int. J. Dermatol..

[135] Böhm M., Luger T.A., Bonsmann G. (1999). Disseminated Superficial Actinic Porokeratosis: Treatment with Topical Tacalcitol. J. Am. Acad. Dermatol..

[136] Abe M., Yokoyama Y., Ishikawa O. (2010). Successful Treatment of Disseminated Superficial Actinic Porokeratosis with Tacalcitol Lotion. J. Dermatol..

[137] Nakamura Y., Yamaguchi M., Nakamura A., Muto M. (2014). Calcipotriol and Adapalene Therapy for Disseminated Superficial Actinic Porokeratosis. Indian J. Dermatol. Venereol. Leprol..

[138] Tchernev G., Chokoeva A.A., Ivanova B., Mangarov H., Vidolova N.G. (2017). Disseminated Superficial Actinic Porokeratosis (DSAP): Significant Improvement after Local Administration of Calcipotriol/Betamethasone Gel?. Wien. Med. Wochenschr..

[139] Noborio R., Morita A. (2012). Split-Face Trial of CO_2_ Laser-Induced Ring Abrasion and High-Dose Tacalcitol in the Treatment of Disseminated Superficial Actinic Porokeratosis. J. Dermatol..

[140] Arun B., Pearson J., Chalmers R. (2011). Disseminated Superficial Actinic Porokeratosis Treated Effectively with Topical Imiquimod 5% Cream. Clin. Exp. Dermatol..

[141] Riad H., Mansour K., Sada H.A., Shaika S.A., Ansari H.A., Mohannadi H.A. (2013). Disseminated Superficial Actinic Porokeratosis on the Face Treated with Imiquimod 5% Cream. Case Rep. Dermatol..

[142] Anderson I., Routt E.T., Jim On S.C. (2017). Disseminated Superficial Actinic Porokeratosis Treated with Ingenol Mebutate Gel 0.05. Cutis.

[143] Nahm W.K., Donohue K.G., Danahy J.F., Badiavas E., Falanga V. (2003). Systemic 5-Fluorouracil Producing an Inflammatory Response in Porokeratosis. J. Eur. Acad. Dermatol. Venereol..

[144] Teixeira S.P., de Nascimento M.M., Bagatin E., Hassun K.M., Talarico S., Michalany N. (2005). The Use of Fluor-Hydroxy Pulse Peel in Actinic Porokeratosis. Dermatol. Surg..

[145] Mendonça R.F., Salem L.A.N., Alves R.O., Hong B., Lellis R.F., Crocco E.I. (2019). Tratamento da poroqueratose actínica superficial disseminada com laser 1340-nm Nd:YAP. Surg. Cosmet. Dermatol..

[146] Narbutt J., Słowik-Rylska M., Sysa-Jędrzejowska A., Słowik-Kwiatkowska I., Lesiak A. (2010). Disseminated Superficial Actinic Porokeratosis. Two Case Reports. Adv. Dermatol. Allergol..

[147] Ross N.A., Rosenbaum L.E., Saedi N., Arndt K.A., Dover J.S. (2016). Disseminated Superficial Actinic Porokeratosis Improved with Fractional 1927-Nm Laser Treatments. J. Cosmet. Laser Ther..

[148] Borroni R.G., Poddighe D., Zecca M., Brazzelli V. (2013). Efficacy of Acitretin for Porokeratosis in a Child with Chronic Cutaneous Graft versus Host Disease. Pediatr. Dermatol..

[149] Park B.J., Oh E.H., Kim J.E., Ko J.Y., Ro Y.S. (2017). Treatment of Disseminated Superficial Actinic Porokeratosis with Oral Alitretinoin. J. Eur. Acad. Dermatol. Venereol..

[150] Kariniemi A.L., Stubb S., Lassus A. (1980). Treatment of Disseminated Superficial Actinic Porokeratosis with a New Aromatic Retinoid (Ro 10-9359). Br. J. Dermatol..

[151] Knobler R.M., Neumann R.A. (1990). Exacerbation of Porokeratosis during Etretinate Therapy. Acta Derm. Venereol..

[152] Carmichael A.J., Tan C.Y. (1990). Digitate Keratoses—A Complication of Etretinate Used in the Treatment of Disseminated Superficial Actinic Porokeratosis. Clin. Exp. Dermatol..

[153] Schwarz T., Seiser A., Gschnait F. (1984). Disseminated Superficial “Actinic” Porokeratosis. J. Am. Acad. Dermatol..

[154] Vergara G., Bañuls J., Botella R., Silvestre J.F., Belinchón I., Betlloch I. (2002). Porokeratosis of the Lower Lip. Eur. J. Dermatol..

[155] Tan L.S., Chong W.-S. (2012). Porokeratosis in Singapore: An Asian Perspective. Australas. J. Dermatol..

[156] Lolis M.S., Marmur E.S. (2008). Treatment of Disseminated Superficial Actinic Porokeratosis (DSAP) with the Q-Switched Ruby Laser. J. Cosmet. Laser Ther..

[157] Itoh M., Nakagawa H. (2007). Successful Treatment of Disseminated Superficial Actinic Porokeratosis with Q-Switched Ruby Laser. J. Dermatol..

[158] Rosenblum J., Roenigk H.H. (2013). Erbium Laser for the Treatment of Disseminated Superficial Actinic Porokeratosis: A Case Report. Dermatol. Surg..

[159] Hou P., Miao Y., Hou S., Cheng S., Hu Z. (2017). Application of Q-Switch Alexandrite Laser Combined with Fractional CO_2_ Laser in Treating Disseminated Superficial Actinic Porokeratosis: Report of Two Cases. Int. J. Clin. Exp. Med..

[160] Kim H.S., Baek J.H., Park Y.M., Kim H.O., Lee J.Y. (2011). Photodynamic Therapy Combined with CO_2_ Laser Vaporization on Disseminated Superficial Actinic Porokeratosis: A Report of 2 Cases on the Face. Ann. Dermatol..

[161] Chrastil B., Glaich A.S., Goldberg L.H., Friedman P.M. (2007). Fractional Photothermolysis: A Novel Treatment for Disseminated Superficial Actinic Porokeratosis. Arch. Dermatol..

[162] Marcuzzi A., Zanin V., Piscianz E., Tricarico P.M., Vuch J., Girardelli M., Monasta L., Bianco A.M., Crovella S. (2012). Lovastatin-Induced Apoptosis Is Modulated by Geranylgeraniol in a Neuroblastoma Cell Line. Int. J. Dev. Neurosci..

[163] Marcuzzi A., Tricarico P.M., Piscianz E., Kleiner G., Vecchi Brumatti L., Crovella S. (2013). Lovastatin Induces Apoptosis through the Mitochondrial Pathway in an Undifferentiated SH-SY5Y Neuroblastoma Cell Line. Cell Death Dis..

[164] Byth L.A., Byth J. (2021). Topical Simvastatin-Cholesterol for Disseminated Superficial Actinic Porokeratosis: An Open-Label, Split-Body Clinical Trial. Australas. J. Dermatol..

[165] Tomsitz D., Biedermann T. (2022). Successful Treatment of Disseminated Superficial Actinic Porokeratosis with Topical 2% Cholesterol/ 2% Lovastatin Cream: A Case Series with 7 Patients. J. Eur. Acad. Dermatol. Venereol..

[166] Leow Y.H., Soon Y.H., Tham S.N. (1996). A Report of 31 Cases of Porokeratosis at the National Skin Centre. Ann. Acad. Med. Singap..

[167] Santa Lucia G., Snyder A., Lateef A., Drohan A., Gregoski M.J., Barton V., Elston D.M. (2023). Safety and Efficacy of Topical Lovastatin Plus Cholesterol Cream vs Topical Lovastatin Cream Alone for the Treatment of Disseminated Superficial Actinic Porokeratosis: A Randomized Clinical Trial. JAMA Dermatol..

[168] Andrews G.C. (1937). Porokeratosis (Mibelli) Disseminated and Superficial Type. Arch. Dermatol. Syphilol..

[169] Kim S.W., Min S.U., Won C.H., Cho S. (2008). Disseminated Superficial Porokeratosisin a Patient with Gastric Cancer. Ann. Dermatol..

[170] Wang J., Liu Y., Liu F., Huang C., Han S., Lv Y., Liu C.-J., Zhang S., Qin Y., Ling L. (2016). Loss-of-Function Mutation in PMVK Causes Autosomal Dominant Disseminated Superficial Porokeratosis. Sci. Rep..

[171] Rosón E., García-Doval I., De La Torre C., Losada A., Rodríguez T., Ocampo C., Cruces M. (2001). Disseminated Superficial Porokeratosis with Mucosal Involvement. Acta Derm. Venereol..

[172] Kanak K., Jaiswal A.K., Reddy P. (2011). Disseminated Superficial and Warty Type of Porokeratosis: A Rare Coexistence. Indian J. Dermatol..

[173] Bhatia R., Gupta V., Khanna N. (2017). Oral Involvement in Disseminated Superficial Porokeratosis. Indian J. Dermatol. Venereol. Leprol..

[174] Kanekura T., Yoshii N. (2006). Eruptive Pruritic Papular Porokeratosis: A Pruritic Variant of Porokeratosis. J. Dermatol..

[175] Makino E., Inaoki M., Fujimoto W. (2005). Inflammatory Stage of Disseminated Superficial Porokeratosis. J. Dermatol..

[176] Sakhiya J.J., Sakhiya D.J., Patel M.R., Daruwala F.R. (2020). Case Report on Rare Clinical Variant of Porokeratosis: Disseminated Superficial Porokeratosis. J. Cutan. Aesthet. Surg..

[177] Navarro V., Pinazo I., Martínez E., Monteagudo C., Jordá E. (2000). Facial Superficial Porokeratosis. Dermatology.

[178] Kanzaki T., Miwa N., Kobayashi T., Ogawa S. (1992). Eruptive Pruritic Papular Porokeratosis. J. Dermatol..

[179] Stork J., Kodetová D. (1997). Disseminated Superficial Porokeratosis: An Eruptive Pruritic Papular Variant. Dermatology.

[180] Goulding J.M.R., Teoh J.K., Carr R.A., Humphreys F., Gee B.C. (2009). Eruptive Disseminated Superficial Porokeratosis with Rapid Resolution: A Drug-Induced Phenomenon?. Clin. Exp. Dermatol..

[181] Patrizi A., Virdi A., Misciali C., Bardazzi F. (2017). Eruptive Pruritic Papular Porokeratosis in a Caucasian Woman: A Transient Inflammatory Stage of Porokeratosis. G. Ital. Dermatol. Venereol..

[182] Morgado-Carrasco D., Feola H., Fustà-Novell X. (2020). Eruptive Pruritic Papular Porokeratosis or Inflammatory Form of Disseminated Superficial Porokeratosis: A New Case and Review of the Literature. Dermatol. Online J..

[183] Soni R., Phiske M., Kanade P., John J., Joshi R., Shylaja S. (2021). Eruptive Pruritic Papular Porokeratosis: A Rare Variant of Porokeratosis. Indian J. Dermatol..

[184] Zhang W.-L., Huang D., Zhang W., Wu Y.-D., Feng S.-Y., Jiang Y.-Q., Li C.-R. (2021). Eruptive Pruritic Papular Porokeratosis. Postepy Dermatol. Alergol..

[185] Wakatabi K., Kakurai M., Yamada T., Umemoto N., Demitsu T., Yoneda K. (2012). Inflammatory Disseminated Superficial Porokeratosis with an Unusual Clinical Feature of the Pruritic, Erythematous Papules Preceding Annular Brownish Pigmentation. J. Dermatol..

[186] Shoimer I., Robertson L.H., Storwick G., Haber R.M. (2014). Eruptive Disseminated Porokeratosis: A New Classification System. J. Am. Acad. Dermatol..

[187] Happle R. (1999). Loss of Heterozygosity in Human Skin. J. Am. Acad. Dermatol..

[188] Korviriyakamol T., Kattipathananpong P., Chunhasewee C., Wessagowit V., Kootiratrakarn T. (2014). Co-Existence of Porokeratosis Variants Concurrent with Bowen’s Disease: Two Rare Cases Report. J. Med. Assoc. Thai.

[189] Palleschi G.M., Torchia D. (2008). Porokeratosis of Mibelli and Superficial Disseminated Porokeratosis. J. Cutan. Pathol..

[190] Raychaudhury T., Valsamma D.P.C. (2011). Giant Porokeratosis. IJDVL.

[191] Kluger N., Dereure O., Guilhou J.-J., Guillot B. (2007). Genital Porokeratosis: Treatment with Diclofenac Topical Gel. J. Dermatol. Treat..

[192] Chun S.I., Lee J.S., Kim N.S., Park K.D. (1995). Disseminated Epidermolytic Acanthoma with Disseminated Superficial Porokeratosis and Verruca Vulgaris in an Immunosuppressed Patient. J. Dermatol..

[193] Vasudevan B., Chatterjee M., Grewal R., Rana V., Lodha N. (2014). A Case of Disseminated Superficial Porokeratosis Associated with Giant Porokeratosis in Pregnancy. Indian J. Dermatol..

[194] Ibbotson S.H. (1996). Disseminated Superficial Porokeratosis: What Is the Association with Ultraviolet Radiation?. Clin. Exp. Dermatol..

[195] Kanitakis J., Misery L., Nicolas J.F., Lyonnet S., Chouvet B., Haftek M., Faure M., Claudy A., Thivolet J. (1994). Disseminated Superficial Porokeratosis in a Patient with AIDS. Br. J. Dermatol..

[196] Romaní J., Pujol R.M., Casanova J.M., de Moragas J.M. (1996). Disseminated Superficial Porokeratosis Developing after Electron-Beam Total Skin Irradiation for Mycosis Fungoides. Clin. Exp. Dermatol..

[197] Dumas M., Corre F., Payancé A., Guedj N., Durand F., Descamps V., Le Bozec P. (2019). Eruptive disseminated superficial porokeratosis associated with acute hepatitis E. Ann. Dermatol. Venereol..

[198] Luelmo-Aguilar J., Gonzalez-Castro U., Mieras-Barcelo C., Castells-Rodellas A. (1992). Disseminated Porokeratosis and Myelodysplastic Syndrome. Dermatology.

[199] Levin R.M., Heymann W.R. (1999). Superficial Disseminate Porokeratosis in a Patient with Myelodysplastic Syndrome. Int. J. Dermatol..

[200] Diluvio L., Campione E., Paterno E.J., Hagman J.H., Anemona L., Orlandi A., Chimenti S. (2008). Acute Onset Disseminated Superficial Porokeratosis Heralding Diffuse Large B-Cell Lymphoma. Eur. J. Dermatol..

[201] Benmously Mlika R., Kenani N., Badri T., Ben Romdhane S., Debbiche A., Souissi A., Ben Ayed M., Mokhtar I., Fenniche S. (2009). Localized Genital Porokeratosis in a Female Patient with Multiple Myeloma. J. Eur. Acad. Dermatol. Venereol..

[202] Rossiello L., Lupoli A., Ruggiero F., Boscaino A., Cozzi R. (2017). Acquired Disseminated Superficial Porokeratosis in a Patient Affected by Chronic Lymphocitic Leukemia. G. Ital. Dermatol. Venereol..

[203] Chokoeva A.A., Wollina U., Lotti T., Maximov G.K., Lozev I., Tchernev G. (2018). Disseminated Porokeratosis with Idiopathic Thrombocytopenia—Case Report and Literature Review of Porokeratosis and Related Disorders. Open Access Maced. J. Med. Sci..

[204] Irie K. (2020). A Case of Disseminated Superficial Porokeratosis in a Patient with Chronic Graft-versus-Host Disease. Dermatol. Online J..

[205] Rio B., Magana C., Le Tourneau A., Bachmeyer C., Lévy V., Hamont N., Diebold J., Zittoun R. (1997). Disseminated Superficial Porokeratosis after Autologous Bone Marrow Transplantation. Bone Marrow Transpl..

[206] Knoell K.A., Patterson J.W., Wilson B.B. (1999). Sudden Onset of Disseminated Porokeratosis of Mibelli in a Renal Transplant Patient. J. Am. Acad. Dermatol..

[207] Kanitakis J., Euvrard S., Claudy A. (2001). Porokeratosis in Organ Transplant Recipients. J. Am. Acad. Dermatol..

[208] Feuerman E.J., Sandbank M. (1979). Disseminated Superficial Porokeratosis in Patients with Pemphigus Vulgaris Treated with Steroids. Acta Derm. Venereol. Suppl..

[209] Bednarek R., Ezra N., Toubin Y., Linos K., Mousdicas N. (2015). Eruptive Disseminated Porokeratosis Associated with Corticosteroid-Induced Immunosuppression. Clin. Exp. Dermatol..

[210] Schena D., Papagrigoraki A., Frigo A., Girolomoni G. (2010). Eruptive Disseminated Porokeratosis Associated with Internal Malignancies: A Case Report. Cutis.

[211] Jung J.-Y., Yeon J.-H., Ryu H.-S., Youn S.-W., Park K.-C., Huh C.-H. (2009). Disseminated Superficial Porokeratosis Developed by Immunosuppression Due to Rheumatoid Arthritis Treatment. J. Dermatol..

[212] Stewart L., Howat A., Coulson I. (2010). Disseminated Superficial Porokeratosis Secondary to Immunosuppression Induced by Etanercept for Extensive Psoriasis. Arch. Dermatol..

[213] Mangas C., Espeli V., Blum R. (2018). A Case of Eruptive Disseminated Porokeratosis in a Cancer Patient after Trastuzumab and Exemestane Treatment: Cancer Related or Drug Induced Phenomenon?. Actas Dermosifiliogr..

[214] Cinotti E., Fiorani D., Provvidenziale L., Miracco C., Calamai V., Danielli R., Rubegni P. (2019). Eruptive Porokeratosis under Nivolumab Adjuvant Treatment for Melanoma. Int. J. Dermatol..

[215] Maredia H., Simkin D., Soni A., Loss M.J. (2020). Eruptive Porokeratosis during Pembrolizumab Treatment of Invasive Cutaneous Squamous Cell Carcinoma. Int. J. Dermatol..

[216] Klager S., Khalil M., Shulman K., Sami N. (2021). Abatacept-Induced Disseminated Superficial Porokeratosis. J. Clin. Rheumatol..

[217] Lu J.D., Mufti A., Sachdeva M., Rahat S., Lansang R.P., Yeung J. (2021). Drugs Associated with Development of Porokeratosis: A Systematic Review. Dermatol. Ther..

[218] Kroiss M.M., Stolz W., Hohenleutner U., Landthaler M. (2000). Disseminated Superficial Porokeratosis Induced by Furosemide. Acta Derm. Venereol..

[219] Ferreira O., Duarte A.F., Baudrier T., Mota A., Azevedo F. (2011). Development of Disseminated Superficial Porokeratosis in a Patient with Complicated Acute Pancreatitis. Dermatol. Online J..

[220] Kono T., Kobayashi H., Ishii M., Nishiguchi S., Taniguchi S. (2000). Synchronous Development of Disseminated Superficial Porokeratosis and Hepatitis C Virus-Related Hepatocellular Carcinoma. J. Am. Acad. Dermatol..

[221] Mundi J.P., Cerullo L., Cotliar J. (2010). Porokeratosis in a Patient with Hepatitis of Unclear Etiology. J. Drugs Dermatol..

[222] Park B.S., Moon S.E., Kim J.A. (1997). Disseminated Superficial Porokeratosis in a Patient with Chronic Liver Disease. J. Dermatol..

[223] Rao A.G., Lakshmi T.S.S., Haritha S. (2002). Disseminated Superficial Porokeratosis. Indian J. Dermatol. Venereol. Leprol..

[224] Shimizu S., Takashima Y., Hotta M., Ito E., Moriuchi R. (2018). Inflammatory Disseminated Superficial Porokeratosis Successfully Controlled with a Combination of Topical Diclofenac Gel and Systemic Etretinate. J. Eur. Acad. Dermatol. Venereol..

[225] Lee S.Y., Lee K.H., Ishii N., Hashimoto T., Kim J.H., Oh C.H., Park K. (2021). Rare Case of Bullous Pemphigoid Occurring on Atrophic Centers of Disseminated Superficial Porokeratosis Lesions. J. Dermatol..

[226] Sen B.B., Ekiz Ö., Rifaioglu E.N., Sen T., Atik E., Dogramaci A.Ç. (2013). Localized Bullous Pemphigoid Occurring on Surgical Scars. Indian J. Dermatol. Venereol. Leprol..

[227] Chun S.H., Kim B.Y., Kim C.M., Park J.B., Ryu H.J. (2017). A Case of Wolf’s Isotopic Response Presenting as Bullous Pemphigoid. Ann. Dermatol..

[228] Pietkiewicz P., Gornowicz-Porowska J., Dmochowska M.B., Dmochowski M. (2013). Malignancy in Relation to Autoimmune Blistering Dermatoses: Molecular and Clinical Aspects. Highlights in Skin Cancer.

[229] Murata Y., Kumano K., Takai T. (2001). Type 2 Segmental Manifestation of Disseminated Superficial Porokeratosis Showing a Systematized Pattern of Involvement and Pronounced Cancer Proneness. Eur. J. Dermatol..

[230] Lee W.J., Kim C.H., Park G.H., Won C.H., Chang S.E., Lee M.W., Choi J.H., Moon K.C. (2010). Disseminated Superficial Porokeratosis in a Patient with Esophageal Cancer. J. Dermatol..

[231] Choi K.H., Kim T.Y. (2009). A Case of Inflammatory Disseminated Superficial Porokeratosis in a Colon Cancer Patient. Ann. Dermatol..

[232] Jiang L.-Y., Guo Z., Kong Y.-L., Luan H., Yu J.-X., Wang K.-Y. (2015). Rectal Cancer Concurrent with Disseminated Superficial Porokeratosis in Three Brothers. J. Dermatol..

[233] Lee H.-W., Oh S.-H., Choi J.-C., Chang S.-E., Lee M.-W., Choi J.-H., Moon K.-C., Koh J.-K. (2006). Disseminated Superficial Porokeratosis in a Patient with Cholangiocarcinoma. J. Am. Acad. Dermatol..

[234] Torres T., Velho G.C., Selores M. (2010). Disseminated Superficial Porokeratosis in a Patient with Cholangiocarcinoma: A Paraneoplastic Manifestation?. An. Bras. Dermatol..

[235] Cannavó S.P., Borgia F., Adamo B., Guarneri B. (2008). Simultaneous Development and Parallel Course of Disseminated Superficial Porokeratosis and Ovarian Cancer: Coincidental Association or True Paraneoplastic Syndrome?. J. Am. Acad. Dermatol..

[236] Hui H.-Z., Wang Y.-J., Cheng J.-R., Mao H., Guo H.-X., Shi B.-J. (2023). A Novel Missense Mutation in the MVK Gene Is Associated with Disseminated Superficial Porokeratosis. Int. J. Dermatol..

[237] Wei S., Yang S., Lin D., Li M., Zhang X., Bu L., Zheng G., Hu L., Kong X., Zhang X. (2004). A Novel Locus for Disseminated Superficial Porokeratosis Maps to Chromosome 18p11.3. J. Investig. Dermatol..

[238] Cao H.M., Wang Z.Y., Zhang G.W., Liu C.F., Pan C.M., Zhao S.X., Song Z.Y., Song H.D., Zhang L. (2012). Identification of a Locus (DSP2) for Disseminated Superficial Porokeratosis at Chromosome 12q21.2-24.21. Clin. Exp. Dermatol..

[239] Awatani K., Hashimoto T., Satoh T. (2021). Eruptive Pruritic Papular Porokeratosis Accompanied by Eosinophilic and Basophilic Infiltrate with Upregulation of Epidermal CCL26/Eotaxin-3 and Thymic Stromal Lymphopoietin. J. Dermatol..

[240] Gao Z., Sun Y. (2021). Dermoscopy Assisting the Diagnosis of Eruptive Disseminated Porokeratosis. J. Dermatol..

[241] Tokat F., Sezer E., Erdemoglu Y., Cetin E.D., Durmaz E.O. (2017). Pruriginous Follicular Porokeratosis. J. Dtsch. Dermatol. Ges..

[242] Pietkiewicz P., Navarrete-Dechent C., Salwowska N., Cantisani C., Goldust M., Errichetti E. (2023). Ultraviolet-Induced Fluorescence Dermatoscopy Reveals Fluorescent Clues in Pitted Keratolysis. Dermatol. Pract. Concept..

[243] Pietkiewicz P., Navarrete-Dechent C., Mayisoğlu H., Jolly G., Kutlu Ö., Errichetti E. (2023). Pink-Red Fluorescence Observed in Ultraviolet-Induced Fluorescence Dermoscopy of Psoriatic Plaques. Dermatol. Pract. Concept..

[244] Pietkiewicz P., Navarrete-Dechent C., Goldust M., Korecka K., Todorovska V., Errichetti E. (2023). Differentiating Fordyce Spots from Their Common Simulators Using Ultraviolet-Induced Fluorescence Dermatoscopy—Retrospective Study. Diagnostics.

[245] Al-Nasiri M., Navarrete-Dechent C., Korecka K., Salwowska N., Goldust M., Pietkiewicz P. (2023). Ultraviolet-Induced Fluorescence Dermatoscopy of Trichobacteriosis Axillaris Reveals Peripilar Yellow-Green Luminescent Concretions. Dermatol. Pract. Concept..

[246] Togawa Y., Yamamoto Y., Matsue H. (2023). Clinical study on the comparison of dermoscopic images using two wavelengths of near-ultraviolet-visible light. JEADV Clin. Pract..

[247] Ramakrishnan R., Vignesh T.A., Durai P.C.T., Narasimhan M. (2022). A Rare Case of Disseminated Superficial Porokeratosis-Case Report. J. Fam. Med. Prim. Care.

[248] Das A., Vasudevan B., Talwar A. (2022). Porokeratosis: An Enigma Beginning to Unravel. Indian J. Dermatol. Venereol. Leprol..

[249] Jurecka W., Neumann R.A., Knobler R.M. (1991). Porokeratoses: Immunohistochemical, Light and Electron Microscopic Evaluation. J. Am. Acad. Dermatol..

[250] Akino S., Okano T., Takeuchi S., Ariizumi Y., Kadono T., Miyagaki T. (2021). Complete Pruritus Relief by Oren-Gedoku-to in Eruptive Pruritic Papular Porokeratosis. J. Dermatol..

[251] Tanaka M., Terui T., Kudo K., Tagami H. (1995). Inflammatory Disseminated Superficial Porokeratosis Followed by Regression. Br. J. Dermatol..

[252] Ito M., Fujiwara H., Maruyama T., Oguro K., Ishihara O., Sato Y. (1991). Morphogenesis of the Cornoid Lamella: Histochemical, Immunohistochemical, and Ultrastructural Study of Porokeratosis. J. Cutan. Pathol..

[253] Matsuta M., Kon S., Sasaki K., Matsuta M. (1997). Immunohistochemical Detection of p21WAF1/CIP1 and P53 Proteins in Formalin-Fixed Paraffin-Embedded Tissue Sections of Squamous Cell Carcinoma of the Skin. J. Dermatol. Sci..

[254] Manganoni A.M., Facchetti F., Gavazzoni R. (1989). Involvement of Epidermal Langerhans Cells in Porokeratosis of Immunosuppressed Renal Transplant Recipients. J. Am. Acad. Dermatol..

[255] Stefanato C.M., Youssef E.A., Cerio R., Kobza-Black A., Greaves M.W. (1993). Atypical Nekam’s Disease—Keratosis Lichenoides Chronica Associated with Porokeratotic Histology and Amyloidosis. Clin. Exp. Dermatol..

[256] Piamphongsant T., Sittapairoachana D. (1974). Localized Cutaneous Amyloidosis in Disseminated Superficial Actinic Porokeratosis. J. Cutan. Pathol..

[257] Hill M.P., Balme B., Gho A., Perrot H. (1992). Porokératose Disséminée Superficielle Avec Amylose Dermique. Ann. Dermatol. Venereol..

[258] Yasuda K., Ikeda M., Ikeda M., Kodama H. (1996). Disseminated Superficial Porokeratosis with Amyloid Deposition. J. Dermatol..

[259] Amantea A., Giuliano M.C., Balus L. (1998). Disseminated Superficial Porokeratosis with Dermal Amyloid Deposits: Case Report and Immunohistochemical Study of Amyloid. Am. J. Dermatopathol..

[260] Demitsu T., Okada O. (1999). Disseminated Superficial Porokeratosis with Dermal Amyloid Deposition. J. Dermatol..

[261] Kim J.H., Yim H., Kang W.H. (2000). Secondary Cutaneous Amyloidosis in Disseminated Superficial Porokeratosis: A Case Report. J. Korean Med. Sci..

[262] Garcia-F-Villalta M.J., Daudén E., Ruiz-Genao D., Fraga J., García-Díez A. (2004). Dermal Amyloid Deposits in Disseminated Superficial Porokeratosis. Acta Derm. Venereol..

[263] Ginarte M., León A., Toribio J. (2005). Disseminated Superficial Porokeratosis with Amyloid Deposits. Eur. J. Dermatol..

[264] Carlesimo M., Rossi A., Fidanza L., Narcisi A., La Pietra M., Mari E., Cacchi C., Camplone G. (2009). Disseminated Superficial Porokeratosis with Dermal Amyloid Deposits. Case Rep. Dermatol..

[265] Inazawa M., Satoh T., Yokozeki H. (2014). Hyperkeratotic Variant of Inflammatory Disseminated Superficial Porokeratosis with Lichenoid Reaction and Extensive Amyloid Deposition. Int. J. Dermatol..

[266] Husein H.-E., Inmaculada R.-M., Vicente C.-A., Eduardo S.-G. (2015). Disseminated Superficial Porokeratosis with Dermal Amyloid Deposits in an Elderly Man: A Rare Entity. J. Dtsch. Dermatol. Ges..

[267] Liu H.T. (2000). Treatment of Lichen Amyloidosis (LA) and Disseminated Superficial Porokeratosis (DSP) with Frequency-Doubled Q-Switched Nd:YAG Laser. Dermatol. Surg..

[268] Tee S.-I., Chong W.-S. (2012). Eruptive Pruritic Papular Porokeratosis. Indian J. Dermatol. Venereol. Leprol..

[269] Klein N., Enk A., Hartschuh W. (2009). Inflammatory stage of disseminated superficial porokeratosis in a 71-year old patient. Hautarzt.

[270] Mu X., Li W., Zhang M., Yang C., Yang X., Li D., Ding Y. (2023). Successful Treatment of Eruptive Pruritic Papular Porokeratosis in the Elderly with Tofacitinib: A Case Report. Clin. Cosmet. Investig. Dermatol..

[271] Jourdan M., Bachmeyer C., Duriez P., Francès C. (2014). Disseminated superficial porokeratosis in a black woman. Ann. Dermatol. Venereol..

[272] Pruitt L.G., Hsia L.-L.B., Burke W.A. (2015). Disseminated Superficial Porokeratosis Involving the Groin and Genitalia in a 72-Year-Old Immunocompetent Man. JAAD Case Rep..

[273] Cha S.H., Park H.J., Lee J.Y., Cho B.K. (2010). Atypical Porokeratosis Developing Following Bone Marrow Transplantation in a Patient with Myelodysplastic Syndrome. Ann. Dermatol..

[274] Shelley W.B., Shelley E.D. (1983). Disseminated Superficial Porokeratosis: Rapid Therapeutic Response to 5-Fluorouracil. Cutis.

[275] Elisia I., Nakamura H., Lam V., Hofs E., Cederberg R., Cait J., Hughes M.R., Lee L., Jia W., Adomat H.H. (2016). DMSO Represses Inflammatory Cytokine Production from Human Blood Cells and Reduces Autoimmune Arthritis. PLoS ONE.

[276] Saki N., Ahramiyanpour N., Heiran A., Alipour S., Parvizi M.M. (2020). Efficacy of Topical Dimethyl Sulfoxide (DMSO) 50% Solution vs Tretinoin 0.5% Cream in Treatment of Patients with Primary Macular Amyloidosis: A Split-Side Single-Blinded Randomized Clinical Trial. Dermatol. Ther..

[277] Padda I.S., Bhatt R., Parmar M. (2023). Tofacitinib. StatPearls.

[278] Chen Y., Xian Y.-F., Loo S., Lai Z., Chan W.Y., Liu L., Lin Z.-X. (2020). Huang-Lian-Jie-Du Extract Ameliorates Atopic Dermatitis-like Skin Lesions Induced by 2,4-Dinitrobenzene in Mice via Suppression of MAPKs and NF-κB Pathways. J. Ethnopharmacol..

[279] Takagi R., Kawano M., Nakagome K., Hashimoto K., Higashi T., Ohbuchi K., Kaneko A., Matsushita S. (2014). Wogonin Attenuates Ovalbumin Antigen-Induced Neutrophilic Airway Inflammation by Inhibiting Th17 Differentiation. Int. J. Inflam..

[280] Zhang R., Zhang H., Shao S., Shen Y., Xiao F., Sun J., Piao S., Zhao D., Li G., Yan M. (2022). Compound Traditional Chinese Medicine Dermatitis Ointment Ameliorates Inflammatory Responses and Dysregulation of Itch-Related Molecules in Atopic Dermatitis. Chin. Med..

[281] Schamroth J.M., Zlotogorski A., Gilead L. (1997). Porokeratosis of Mibelli. Overview and Review of the Literature. Acta Derm. Venereol..

[282] Ferreira F.R., Santos L.D.N., Tagliarini F.A.N.M., Lira M.L. (2013). de A. Porokeratosis of Mibelli—Literature Review and a Case Report. An. Bras. Dermatol..

[283] Nabai H., Mehregan A.H. (1979). Porokeratosis of Mibelli. A Report of Two Unusual Cases. Dermatologica.

[284] Schramm P., Bork K. (1982). Naeviform porokeratosis—Only a morphologic variant of porokeratosis of Mibelli (author’s transl). Z. Hautkr.

[285] Guillot P., Taieb A., Fontan I., Bilhou-Nabera C., Viard E., Renaud P., Maleville J. (1991). Linear porokeratosis of Mibelli in monozygotic twin girls. Ann. Dermatol. Venereol..

[286] Götz A., Kopera D., Wach F., Hohenleutner U., Landthaler M. (1999). Porokeratosis Mibelli gigantea: Case report and literature review. Hautarzt.

[287] Schaller M., Korting H.C., Kollmann M., Kind P. (1996). The Hyperkeratotic Variant of Porokeratosis Mibelli Is a Distinct Entity: Clinical and Ultrastructural Evidence. Dermatology.

[288] Wagner G., Meyer V., Sachse M.M. (2016). Verrucous variant of porokeratosis of Mibelli as a differential diagnosis of psoriasis vulgaris. Hautarzt.

[289] de Wet J., Swart M., Jordaan H.F., Schneider J.W., Mulder S., Visser W.I. (2020). An Unusual Case of Generalized Hyperkeratotic and Verrucous Porokeratosis. JAAD Case Rep..

[290] Yu H.-J., Park K.-T., Oh D.-H., Kim J.-S., Park Y.-W. (2006). A Case of the Hyperkeratotic Variant of Porokeratosis Mibelli. J. Dermatol..

[291] Uenishi T., Teramura K., Kitamura M., Fujii N., Nakanishi G., Tanaka T., Uehara M. (2010). Hyperkeratotic Variant of Porokeratosis Mibelli with Dermal Amyloid Deposits. J. Dermatol..

[292] Kuno Y., Sato K., Tsuji T. (1999). Porokeratosis of Mibelli Associated with Dermal Amyloid Deposits. Br. J. Dermatol..

[293] Ghorpade A. (2010). Localized Actinic Nasal Porokeratosis. Clin. Exp. Dermatol..

[294] Perlis C., Robinson-Bostom L., Telang G.H., DiGiovanna J. (2006). A Thick Lichenified Plaque on the Ventral Penile Shaft. Penile Porokeratosis of Mibelli. Arch. Dermatol..

[295] Neri I., Marzaduri S., Passarini B., Patrizi A. (1995). Genital Porokeratosis of Mibelli. Genitourin. Med..

[296] Miranda S.M.B., De Miranda J.N.R., De Souza Filho J.B. (2004). Facial Porokeratosis Characterized by Destructive Lesions. Int. J. Dermatol..

[297] Hernández-Bel P., Sanmartín-Jimenez O., Sorni-Bröker G., Guillén-Barona C. (2010). Labial Porokeratosis. Am. J. Dermatopathol..

[298] Ahmed G., Ganguly S., Khare S. (2022). Atrophic Lingual Plaque in Father-Son Duo. Indian Dermatol. Online J..

[299] Yong A.S.W., Singh M., Goulding J.M.R., Swale V.J. (2009). Follicular Porokeratosis of Mibelli on the Buttocks. Clin. Exp. Dermatol..

[300] Robati R.M., Rahmati-Roodsari M., Ayatollahi A., Hejazi S. (2011). Facial and Bilateral Acral Porokeratosis with Nail Dystrophy: A Case Report. Dermatol. Online J..

[301] Nenoff P., Arnold T., Nenning H., Hindermann W. (2011). Centrifugal plaques with central atrophy and peripheral scale on the scalp. Hautarzt.

[302] Buzina D.S., Rajič S., Radoš J., Marinović B., Lipozenčić J. (2010). Focal Porokeratosis of Nuchae: Case Report. Acta Dermatovenerol. Croat..

[303] Odeyinde S., Belcher H. (2012). Isolated Single Digit Porokeratosis of Mibelli: An Unusual Case. Dermatol. Online J..

[304] Pawar M. (2017). Onychodystrophy Due to Porokeratosis of Mibelli: A Rare Association. Acta Dermatovenerol. Alp. Pannonica Adriat..

[305] Yendo T.M., Gabbi T.V.B., Nico M.M.S. (2021). Porokeratosis of the Nail Unit: Case Series and Review. Skin. Appendage Disord..

[306] Karthikeyan K., Thappa D.M., Udayashankar C. (2003). Porokeratosis of Mibelli with Nail Dystrophy. J. Dermatol..

[307] Kim D.S., Roh M.R., Lee J.H., Lee K.H. (2007). Pterygium Unguis Formation in Porokeratosis of Mibelli. Br. J. Dermatol..

[308] Rajesh G., Devan P., Keerthi S., Karthikeyan K. (2018). Acral Porokeratosis Associated with Anonychia. Indian J. Dermatol. Venereol. Leprol..

[309] Handjani F., Shahbaz S., Aslani F.S., Gheisari F., Mozaffarian K., Kasraee B. (2010). Porokeratosis of Mibelli with Mutilation: A Case Report. Cutis.

[310] Alexis A.F., Busam K., Myskowski P.L. (2006). Porokeratosis of Mibelli Following Bone Marrow Transplantation. Int. J. Dermatol..

[311] Mizukawa Y., Shiohara T. (2001). Onset of Porokeratosis of Mibelli in Organ Transplant Recipients: Lack of a Search for Transmissible Agents in These Patients. J. Am. Acad. Dermatol..

[312] Raychaudhuri S.P., Smoller B.R. (1992). Porokeratosis in Immunosuppressed and Nonimmunosuppressed Patients. Int. J. Dermatol..

[313] Han Y.W., Kim Y.J., Kim H.O., Park Y.M. (2008). Clinical Study of Porokeratosis Associated with Immunosuppressive Therapy in Renal Transplant Recipients. Ann. Dermatol..

[314] Yazkan F., Turk B.G., Dereli T., Kazandi A.C. (2006). Porokeratosis of Mibelli Induced by Topical Corticosteroid. J. Cutan. Pathol..

[315] Protopsaltis J., Katsantonis J.C., Kokkoris S., Agapitos E., Lavranos G., Korantzopoulos P., Giannoulis G. (2008). Isolated Primary Cardiac Amyloidosis Associated with Porokeratosis of Mibelli. Int. J. Cardiol..

[316] Yilmaz M., Erdoğan B., Özaslan M., Varnali E., Kavak A., Sakiz D. (2022). Coexistence of Multiple Porokeratoma and Porokeratosis of Mibelli. Ann. Dermatol. Venereol..

[317] Dippel E., Haas N., Czarnetzki B.M. (1994). Porokeratosis of Mibelli Associated with Active Chronic Hepatitis and Vitiligo. Acta Derm. Venereol..

[318] Ma Y., Li C., Wu J., Cui P., Lin L., Feng S. (2015). Coexistence of Porokeratosis Ptychotropica with Porokeratosis of Mibelli in a Chinese Man. Postepy Dermatol. Alergol..

[319] Eng A.M., Kolton B. (1975). Generalized Eruptive Porokeratosis of Mibelli with Associated Psoriasis. J. Cutan. Pathol..

[320] Beer W.E., Smith N.P. (1984). Hyperkeratotic Porokeratosis (Mibelli) with Psoriasis—Response to an Aromatic Retinoid. Clin. Exp. Dermatol..

[321] De Simone C., Paradisi A., Massi G., Proietti I., Capponi A., Amerio P.L., Capizzi R. (2007). Giant Verrucous Porokeratosis of Mibelli Mimicking Psoriasis in a Patient with Psoriasis. J. Am. Acad. Dermatol..

[322] Ehsani A.H., Shakoei S., Ranjbar M. (2014). Giant Porokeratosis of Mibelli with Squamous Cell Carcinoma. Indian J. Dermatol. Venereol. Leprol..

[323] Zhang F., Bai W., Sun S., Li N., Zhang X. (2020). Squamous Cell Carcinoma Arising from Giant Porokeratosis and Rare Postoperative Recurrence and Metastasis: A Case Report. Medicine.

[324] Hanumanthayya K., Magavi S., Tophakhane R., Rathod R. (2003). Coexistence of Disseminated Superficial and Giant Porokeratosis of Mibelli with Squamous Cell Carcinoma. Indian J. Dermatol. Venereol. Leprol..

[325] Bhunia D., Ghosh S., Rudra O., Biswas S.K., Agarwal M., Ghosh A. (2016). Keratoacanthoma Arising over Margin of Porokeratosis of Mibelli: A New Association?. Indian J. Dermatol..

[326] Saritha M., Kumari R., Thappa D.M., Rajesh N.G., Verma S.K. (2013). Benign Giant Cutaneous Horn Formed by Giant Porokeratosis of Mibelli with Dysplasia. IJDVL.

[327] Breneman D.L., Breneman J.C. (1993). Cutaneous T-Cell Lymphoma Mimicking Porokeratosis of Mibelli. J. Am. Acad. Dermatol..

[328] Elfatoiki F.Z., Soussi W., Chiheb S., Jabri L., Benchikhi H. (2015). Cutaneous Sarcoidosis Simulating Porokeratosis of Mibelli. Pan Afr. Med. J..

[329] Zeng K., Zhang Q.-G., Li L., Duan Y., Liang Y.-H. (2014). Splicing Mutation in MVK Is a Cause of Porokeratosis of Mibelli. Arch. Dermatol. Res..

[330] Song N.J., Luan J., Zhang Z.H. (2017). Updating and Identifying a Novel Mutation in the PMVK Gene in Classic Porokeratosis of Mibelli. Clin. Exp. Dermatol..

[331] Occella C., Bleidl D., Nozza P., Mascelli S., Raso A., Gimelli G., Gimelli S., Tassano E. (2013). Identification of an Interstitial 18p11.32-P11.31 Duplication Including the EMILIN2 Gene in a Family with Porokeratosis of Mibelli. PLoS ONE.

[332] Hivnor C., Williams N., Singh F., VanVoorhees A., Dzubow L., Baldwin D., Seykora J. (2004). Gene Expression Profiling of Porokeratosis Demonstrates Similarities with Psoriasis. J. Cutan. Pathol..

[333] Jha A.K., Sonthalia S., Lallas A. (2017). Dermoscopy of Porokeratosis of Mibelli. Indian Dermatol. Online J..

[334] Pizzichetta M.A., Canzonieri V., Massone C., Soyer H.P. (2009). Clinical and Dermoscopic Features of Porokeratosis of Mibelli. Arch. Dermatol..

[335] Uhara H., Kamijo F., Okuyama R., Saida T. (2011). Open Pores with Plugs in Porokeratosis Clearly Visualized with the Dermoscopic Furrow Ink Test: Report of 3 Cases. Arch. Dermatol..

[336] Ahlgrimm-Siess V., Koller S., El Shabrawi-Caelen L., Hofmann-Wellenhof R., Kerl H. (2008). New Diagnostic Methods in Dermatopathology: In Vivo Reflectance Confocal Microscopy. J. Dtsch. Dermatol. Ges..

[337] Marghescu S., Anton-Lamprecht I., Melz-Rothfuss B. (1987). Disseminated Bilateral Hyperkeratotic Variant of Porokeratosis Mibelli. Arch. Dermatol. Res..

[338] Sato A., Masu S., Seiji M. (1980). Electron Microscopic Studies of Porokeratosis Mibelli—Civatte Bodies and Amyloid Deposits in the Dermis. J. Dermatol..

[339] Sato A., Böhm W., Bersch A. (1977). Hyperkeratotic Form of Porokeratosis Mibelli. Dermatologica.

[340] Giuliodori K., Campanati A., Ganzetti G., Conocchiari L., Cataldi I., Simonetti O., Giangiacomi M., Offidani A. (2011). The Successful Off-Label Use of Photodynamic Therapy for Classic Porokeratosis of Mibelli: Case Report. Dermatol. Ther..

[341] Gutiérrez Paredes E., Bella Navarro R., Montesinos Villaescusa E., Jordá Cuevas E. (2013). Porokeratosis of Mibelli: A New Indication for Photodynamic Therapy?. Actas Dermosifiliogr..

[342] Levitt J., Emer J.J., Emanuel P.O. (2010). Treatment of Porokeratosis of Mibelli with Combined Use of Photodynamic Therapy and Fluorouracil Cream. Arch. Dermatol..

[343] Chowdhury M.M., Inaloz H.S., Holt P.J. (2000). A Scaly Macule on the Bridge of the Nose of a 15-Year-Old Boy. Pediatr. Dermatol..

[344] Dereli T., Ozyurt S., Ozturk G. (2004). Porokeratosis of Mibelli: Successful Treatment with Cryosurgery. J. Dermatol..

[345] Levell N.J., Bewley A.P., Levene G.M. (1994). Porokeratosis of Mibelli on the Penis, Scrotum and Natal Cleft. Clin. Exp. Dermatol..

[346] Harrison S., Sinclair R. (2003). Porokeratosis of Mibelli: Successful Treatment with Topical 5% Imiquimod Cream. Australas. J. Dermatol..

[347] Shahmoradi Z., Sadeghiyan H., Pourazizi M., Saber M., Abtahi-Naeini B. (2015). Recalcitrant Digital Porokeratosis of Mibelli: A Successful Surgical Treatment. N. Am. J. Med. Sci..

[348] McCullough T.L., Lesher J.L. (1994). Porokeratosis of Mibelli: Rapid Recurrence of a Large Lesion after Carbon Dioxide Laser Treatment. Pediatr. Dermatol..

[349] Rabbin P.E., Baldwin H.E. (1993). Treatment of Porokeratosis of Mibelli with CO_2_ Laser Vaporization versus Surgical Excision with Split-Thickness Skin Graft. A Comparison. J. Dermatol. Surg. Oncol..

[350] Ottoni L.d.Q., Kakizaki P., Pinheiro R.R., Sittart J.A.d.S., Valente N.Y.S. (2016). Porokeratosis of Mibelli in an HIV-Positive Patient. An. Bras. Dermatol..

[351] Liu Y., Wang Y., Nie X., Yan L., Liu Y., Zhou J., Tong W., Tao J. (2012). Treatment of Classic Porokeratosis of Mibelli with Q-Switched Ruby Laser. Eur. J. Dermatol..

[352] Spencer J.M., Katz B.E. (1992). Successful Treatment of Porokeratosis of Mibelli with Diamond Fraise Dermabrasion. Arch. Dermatol..

[353] Venkatarajan S., LeLeux T.M., Yang D., Rosen T., Orengo I. (2010). Porokeratosis of Mibelli: Successful Treatment with 5 Percent Topical Imiquimod and Topical 5 Percent 5-Fluorouracil. Dermatol. Online J..

[354] McDonald S.G., Peterka E.S. (1983). Porokeratosis (Mibelli): Treatment with Topical 5-Fluorouracil. J. Am. Acad. Dermatol..

[355] Zasada M., Budzisz E. (2019). Retinoids: Active Molecules Influencing Skin Structure Formation in Cosmetic and Dermatological Treatments. Postepy Dermatol. Alergol..

[356] Ratka P., Słoboda T., Szubstarski F., Dudzik W. (1988). Favorable outcome of the treatment of Mibelli’s porokeratosis linearis using occlusive dressings with retinoic acid. Przegl Dermatol..

[357] Laureano A., Macias V.C., Pacheco A. (2012). Poroqueratose de Mibelli—Um Caso Clínico. Revista SPDV.

[358] Torne Escasany R., Villodres Ramos E., Umbert Millet P., Guix M. (1983). Generalized Mibelli’s porokeratosis in transformation to spinocellular carcinoma. Clinical, histologic and ultrastructural study. Favorable response to RO-109359. Med. Cutan. Ibero Lat. Am..

[359] Bundino S., Zina A.M. (1980). Disseminated Porokeratosis Mibelli Treated with RO 10-9359. A Study of Two Cases with Ultrastructural Remarks. Dermatologica.

[360] Hacham-Zadeh S., Holubar K. (1985). Etretinate in the Treatment of Disseminated Porokeratosis of Mibelli. Int. J. Dermatol..

[361] Campbell J.P., Voorhees J.J. (1985). Etretinate Improves Localized Porokeratosis of Mibelli. Int. J. Dermatol..

[362] Verma K.K., Singh O.P. (1994). Dexamethasone Pulse Treatment in Disseminated Porokeratosis of Mibelli. J. Dermatol. Sci..

[363] Gracia-Cazaña T., Vera-Álvarez J., García-Patos V., Gilaberte Y. (2015). Imiquimod and Photodynamic Therapy Are Useful in the Treatment of Porokeratosis in Children with Bone Marrow Transplantation. Pediatr. Dermatol..

[364] Montes-De-Oca-Sánchez G., Tirado-Sánchez A., García-Ramírez V. (2006). Porokeratosis of Mibelli of the Axillae: Treatment with Topical Imiquimod. J. Dermatolog Treat..

[365] Erbagci Z., Tuncel A.A., Erbagci I. (2006). Successful Treatment of Porokeratosis with Topical Imiquimod in 2 Immunosuppressed Cases. J. Drugs Dermatol..

[366] Jain S. (2006). Successful Treatment of Porokeratosis of Mibelli with Imiquimod 5% Cream. Clin. Exp. Dermatol..

[367] Esser A.C., Pittelkow M.R., Randle H.W. (2006). Human Papillomavirus Isolated from Transplant-Associated Porokeratoses of Mibelli Responsive to Topical 5% Imiquimod Cream. Dermatol. Surg..

[368] Gajic B., Tang K., Whitfeld M. (2011). Porokeratosis of Mibelli: Involution and Resolution with 5% Imiquimod Cream. Australas. J. Dermatol..

[369] Ataseven A., Öztürk P., Dilek N., Küçükosmanoğlu I. (2012). Localized Actinic Nasal Porokeratosis: A Case Report. Acta Dermatovenerol. Alp. Pannonica Adriat..

[370] Kindem S., Serra-Guillén C., Sorní G., Guillén C., Sanmartín O. (2015). Treatment of Porokeratosis of Mibelli with Ingenol Mebutate: A Possible New Therapeutic Option. JAMA Dermatol..

[371] Goldner R. (1971). Zosteriform Porokeratosis of Mibelli. Arch. Dermatol..

[372] Rahbari H., Cordero A.A., Mehregan A.H. (1974). Linear Porokeratosis. A Distinctive Clinical Variant of Porokeratosis of Mibelli. Arch. Dermatol..

[373] Sommerlad M., Lock A., Moir G., McGregor J., Bull R., Cerio R., Harwood C. (2016). Linear Porokeratosis with Multiple Squamous Cell Carcinomas Successfully Treated by Electrochemotherapy. Br. J. Dermatol..

[374] Agrawal S.N., Pawar P.C., Dhillan P.V. (2014). Linear Porokeratosis: An Unusual Presentation. Indian J. Dermatol..

[375] Bogaert M.A., Hogan D.J. (1991). Linear Porokeratosis in a 74-Year-Old Woman. J. Am. Acad. Dermatol..

[376] Fisher C.A., LeBoit P.E., Frieden I.J. (1995). Linear Porokeratosis Presenting as Erosions in the Newborn Period. Pediatr. Dermatol..

[377] Nayak M.K., Dhanta A., Hazarika N., Kumar A. (2020). Linear Systematized Porokeratosis—A Rare Case and Dermoscopic Clues to Diagnosis. Indian Dermatol. Online J..

[378] Yang J., Du Y.-Q., Fang X.-Y., Li B., Xi Z.-Q., Feng W.-L. (2022). Linear Porokeratosis of the Foot with Dermoscopic Manifestations: A Case Report. World J. Clin. Cases.

[379] Hashimoto T., Moriyama Y., Satoh T. (2021). Linear Porokeratosis with Severe Itch Accompanied by Lesional Upregulation of Interleukin 31, Thymic Stromal Lymphopoietin, and Periostin. Eur. J. Dermatol..

[380] Kienast A.K., Hoeger P.H. (2009). Penile Linear Porokeratosis in a Child: A Case Report. Pediatr. Dermatol..

[381] Yadav P., Sanke S., Mendiratta V., Chander R. (2022). Generalized Linear Porokeratosis in a Bilateral Distribution: An Unusual Presentation. Skinmed.

[382] Kudligi C., Bhagwat P.V., Giriyan S.S., Eshwarrao M.S. (2011). Unilateral systematized linear porokeratosis: A report of a rare case. Indian J. Dermatol..

[383] Garg T., Ramchander, Varghese B., Barara M., Nangia A. (2011). Generalized Linear Porokeratosis: A Rare Entity with Excellent Response to Acitretin. Dermatol. Online J..

[384] Escanilla-Figueroa C., Jimeno-Ortega I., Fuenzalida-Wong H., Chávez-Rojas F. (2018). Generalized Linear Porokeratosis. An. Bras. Dermatol..

[385] McMillan G.L., Krull E.A., Mikhail G.R. (1976). Linear Porokeratosis with Giant Cornoid Lamella. Arch. Dermatol..

[386] Kono M., Yokoyama N., Ogawa Y., Takama H., Sugiura K., Akiyama M. (2016). Unilateral Generalized Linear Porokeratosis with Nail Dystrophy. J. Dermatol..

[387] Tseng S.S., Levit E.K., Ilarda I., Garzon M.C., Grossman M.E. (2002). Linear Porokeratosis with Underlying Bony Abnormalities. Cutis.

[388] Chen H.-H., Liao Y.-H. (2002). Onychodystrophy in Congenital Linear Porokeratosis. Br. J. Dermatol..

[389] Dervis E., Demirkesen C. (2006). Generalized Linear Porokeratosis. Int. J. Dermatol..

[390] Kohara Y., Takeo T., Oshima Y., Akita Y., Tamada Y., Watanabe D. (2011). Linear Porokeratosis with Nail Dystrophy. Eur. J. Dermatol..

[391] Blue E., Abbott J., Bowen A., Cipriano S.D. (2021). Linear Porokeratosis with Bone Abnormalities Treated with Compounded Topical 2% Cholesterol/2% Lovastatin Ointment. Pediatr. Dermatol..

[392] Wei B., Liu M., Qu L., Xiao T., Chen H.-D., He C. (2010). Congenital Linear Porokeratosis with Pseudoainhum. Eur. J. Dermatol..

[393] Witkowski J.A., Parish L.C. (1982). Linear Porokeratosis Presenting as Mosaic Plantar Warts. Int. J. Dermatol..

[394] Hunt S.J., Sharra W.G., Abell E. (1991). Linear and Punctate Porokeratosis Associated with End-Stage Liver Disease. J. Am. Acad. Dermatol..

[395] Tappel A.C., Tiwari N., Zlotoff B. (2019). Linear Porokeratosis Associated with Bardet-Biedl Syndrome: A Case Report. Pediatr. Dermatol..

[396] Verma S.B. (2009). A Rare Case of Porokeratosis Ptychotropica and Coexistent Linear Porokeratosis in a 10-Year-Old Boy. Clin. Exp. Dermatol..

[397] Schwarz N., Stadie V., Kreft B., Happle R., Marsch W.C., Fiedler E. (2012). Systematized Linear Porokeratosis: Concept of Type 2 Segmental Manifestation Implies an Increased Cancer Risk. J. Dermatol..

[398] Sasson M., Krain A.D. (1996). Porokeratosis and Cutaneous Malignancy. A Review. Dermatol. Surg..

[399] Happle R. (1997). Cancer Proneness of Linear Porokeratosis May Be Explained by Allelic Loss. Dermatology.

[400] Saleva-Stateva M., Hess M., Technau-Hafsi K., Weibel L., Badea M.-A., Boente M.D.C., Theiler M., Fiandrino M.J., Hoeger P., Zimmer A. (2021). Molecular Characterization and Natural History of Linear Porokeratosis: A Case Series. J. Am. Acad. Dermatol..

[401] Atzmony L., Khan H.M., Lim Y.H., Paller A.S., Levinsohn J.L., Holland K.E., Mirza F.N., Yin E., Ko C.J., Leventhal J.S. (2019). Second-Hit, Postzygotic PMVK and MVD Mutations in Linear Porokeratosis. JAMA Dermatol..

[402] Paller A.S., van Steensel M.A.M., Rodriguez-Martín M., Sorrell J., Heath C., Crumrine D., van Geel M., Cabrera A.N., Elias P.M. (2011). Pathogenesis-Based Therapy Reverses Cutaneous Abnormalities in an Inherited Disorder of Distal Cholesterol Metabolism. J. Investig. Dermatol..

[403] Li D., Technau-Hafsi K., Giehl K., Hoeger P.H., Has C. (2023). Targeted Anti-IL-17 Therapy for Linear Porokeratosis. Br. J. Dermatol..

[404] Errichetti E., Francesco V.D., Pegolo E., Stinco G. (2017). Dermoscopy in Assisting the Diagnosis of Linear Porokeratosis. J. Dermatol..

[405] Turkmen M., Gerceker Turk B., Kilinc Karaarslan I., Kandiloglu G., Ozdemir F. (2019). Dermoscopic Features of Linear Porokeratosis: Different Aspects in Its Development. J. Dermatol. Res. Ther..

[406] Kovacikova Curkova A., Hegyi J., Kozub P., Szep Z., D’Erme A.M., Simaljakova M. (2014). A Case of Linear Porokeratosis Treated with Photodynamic Therapy with Confocal Microscopy Surveillance. Dermatol. Ther..

[407] Vaishnani J.B., Bosamiya S.S., Sapariya B.J., Udhreja P.R. (2011). Linear Porokeratosis with Follicular Involvement. Indian J. Dermatol..

[408] Minami-Hori M., Ishida-Yamamoto A., Iizuka H. (2009). Cornoid Lamellae Associated with Follicular Infundibulum and Acrosyringium in Porokeratosis. J. Dermatol..

[409] Malhotra S.K., Puri K.J.P.S., Goyal T., Chahal K.S. (2007). Linear Porokeratosis. Dermatol. Online J..

[410] Taniguchi Y., Yuasa T., Shimizu M. (1993). Linear Porokeratosis. J. Dermatol..

[411] Barnett J.H. (1986). Linear Porokeratosis: Treatment with the Carbon Dioxide Laser. J. Am. Acad. Dermatol..

[412] Saleva-Stateva M., Weibel L., Theiler M., Balabanova M., Boente M.C., Has C. (2021). Lack of Effect of Topical Statins in Linear Porokeratosis. J. Eur. Acad. Dermatol. Venereol..

[413] Alster T.S., Nanni C.A. (1999). Successful Treatment of Porokeratosis with 585 Nm Pulsed Dye Laser Irradiation. Cutis.

[414] Cohen P.R., Held J.L., Katz B.E. (1990). Linear Porokeratosis: Successful Treatment with Diamond Fraise Dermabrasion. J. Am. Acad. Dermatol..

[415] Bhushan M., Craven N.M., Beck M.H., Chalmers R.J. (1999). Linear Porokeratosis of Mibelli: Successful Treatment with Cryotherapy. Br. J. Dermatol..

[416] Gonçalves S.V.C.B., Valente N.Y.S., de Oliveira Junior J.V., Paiva D.L.M. (2012). Case for Diagnosis. An. Bras. Dermatol..

[417] Casale F., Tchanque-Fossuo C.N., Durkin J.R. (2021). Successful Clearance of Linear Porokeratosis with Aminolevulinic Acid and Pulsed Dye Laser. Dermatol. Surg..

[418] Garrido-Colmenero C., Ruiz-Villaverde R., Martínez-García E., Aneiros-Fernández J., Tercedor-Sánchez J. (2015). Photoletter to the Editor: Response of Linear Porokeratosis to Photodynamic Therapy in an 11-Year-Old Girl. J. Dermatol. Case Rep..

[419] García-Navarro X., Garcés J.R., Baselga E., Alomar A. (2009). Linear Porokeratosis: Excellent Response to Photodynamic Therapy. Arch. Dermatol..

[420] Weidner T., Illing T., Miguel D., Elsner P. (2017). Treatment of Porokeratosis: A Systematic Review. Am. J. Clin. Dermatol..

[421] Nazarian R.S., Hosseinipour M., Amin B., Cohen S.R. (2020). Linear Porokeratosis Presenting in Adulthood: A Diagnostic Challenge: A Case Report. SAGE Open Med. Case Rep..

[422] Alakeel A., Dawari S., Alhumidi A., Alekrish K. (2023). Combining Isotretinoin and Topical Cholesterol/Atorvastatin in the Treatment of Linear Porokeratosis: A Case Report. Cureus.

[423] Hong J.-B., Hsiao C.-H., Chu C.-Y. (2009). Systematized Linear Porokeratosis: A Rare Variant of Diffuse Porokeratosis with Good Response to Systemic Acitretin. J. Am. Acad. Dermatol..

[424] Pehamberger H., Esca S.A., Holubar K. (1980). Treatment of hyperkeratotic dermatoses with an oral aromatic retinoid (Ro 10-9359). Z. Hautkr.

[425] Goldman G.D., Milstone L.M. (1995). Generalized Linear Porokeratosis Treated with Etretinate. Arch. Dermatol..

[426] Parks A.C., Conner K.J., Armstrong C.A. (2014). Long-Term Clearance of Linear Porokeratosis with Tacrolimus, 0.1%, Ointment. JAMA Dermatol..

[427] Kim C. (2005). Linear Porokeratosis. Dermatol. Online J..

[428] Grover C., Goel A., Nanda S., Khurana N., Reddy B.S.N. (2005). A Case of Extensive Linear Porokeratosis with Evaluation of Topical Tretinoin versus 5-Flourouracil as Treatment Modalities. J. Dermatol..

[429] Diep D., Janitz T., Kannan K.S., Crane A., Aluri B., Wright K., Baker W. (2022). Bilateral Linear Porokeratosis Treated with Topical Cholesterol 2%/Lovastatin 2%. Cureus.

[430] Ahn S.-J., Lee H.-J., Chang S.-E., Lee M.-W., Choi J.-H., Moon K.-C., Koh J.-K. (2007). Case of Linear Porokeratosis: Successful Treatment with Topical 5% Imiquimod Cream. J. Dermatol..

[431] Buhle A.C., Fagan K.K., Johnson N.M., Grider D.J. (2022). Treating Linear Porokeratosis with Topical Lovastatin/Cholesterol Cream. Dermatol. Online J..

[432] Guss S.B., Osbourn R.A., Lutzner M.A. (1971). Porokeratosis Plantaris, Palmaris, et Disseminata. A Third Type of Porokeratosis. Arch. Dermatol..

[433] Irisawa R., Yamazaki M., Yamamoto T., Tsuboi R. (2012). A Case of Porokeratosis Plantaris Palmaris et Disseminata and Literature Review. Dermatol. Online J..

[434] Jedlowski P.M., Rainwater G., Paek S.Y. (2020). Punctate Porokeratosis-Pruritic and Hyperkeratotic Papules on the Palms and Feet. Bayl. Univ. Med. Cent. Proc..

[435] Lanka P., Lanka L.R., Manivachagam D. (2015). Punctate Porokeratosis Palmaris et Plantaris. Indian J. Dermatol..

[436] Roberts L.C., DeVillez R.L. (1984). Congenital Unilateral Punctate Porokeratosis. Am. J. Dermatopathol..

[437] Udare S., Hemmady K. (2016). Clinical and Dermatoscopic Features of Porokeratosis Palmaris et Plantaris. Indian Dermatol. Online J..

[438] Kanitakis J. (2014). Porokeratoses: An Update of Clinical, Aetiopathogenic and Therapeutic Features. Eur. J. Dermatol..

[439] Shaw J.C., White C.R. (1984). Porokeratosis Plantaris Palmaris et Disseminata. J. Am. Acad. Dermatol..

[440] Jensen J.-M., Egberts F., Proksch E., Hauschild A. (2005). Disseminated Porokeratosis Palmaris and Plantaris Treated with Imiquimod Cream to Prevent Malignancy. Acta Derm. Venereol..

[441] Patrizi A., Passarini B., Minghetti G., Masina M. (1989). Porokeratosis Palmaris et Plantaris Disseminata: An Unusual Clinical Presentation. J. Am. Acad. Dermatol..

[442] Hartman R., Mandal R., Sanchez M., Stein J.A. (2010). Porokeratosis Plantaris, Palmaris, et Disseminata. Dermatol. Online J..

[443] Hayashi K., Iinuma S., Ishida-Yamamoto A. (2023). Porokeratosis Plantaris Palmaris et Disseminata with Pruritic Inflammatory Changes. JEADV Clin. Pract..

[444] Sengupta S., Das J.K., Gangopadhyay A. (2005). Multicentric Squamous Cell Carcinoma over Lesions of Porokeratosis Palmaris et Plantaris Disseminata and Giant Porokeratosis. Indian J. Dermatol. Venereol. Leprol..

[445] Ruocco V., Satriano R.A., Florio M., Pettinato G., Pisani M. (1990). Porokeratosis palmaris et plantaris disseminata with squamous cell carcinoma. G. Ital. Dermatol. Venereol..

[446] Seishima M., Izumi T., Oyama Z., Maeda M. (2000). Squamous Cell Carcinoma Arising from Lesions of Porokeratosis Palmaris et Plantaris Disseminata. Eur. J. Dermatol..

[447] Marschalkó M., Somlai B. (1986). Porokeratosis Plantaris, Palmaris, et Disseminata. Arch. Dermatol..

[448] Brown F.C. (1971). Punctate Keratoderma. Arch. Dermatol..

[449] Herman P.S. (1973). Punctate Porokeratotic Keratoderma. Dermatologica.

[450] Rahbari H., Cordero A.A., Mehregan A.H. (1977). Punctate Porokeratosis. A Clinical Variant of Porokeratosis of Mibelli. J. Cutan. Pathol..

[451] Himmelstein R., Lynfield Y.L. (1984). Punctate Porokeratosis. Arch. Dermatol..

[452] Ryan C., Bennett R., Davis M., Yan S., Aaron D.M. (2023). A Rare Case of Punctate Porokeratosis Treated with Topical Lovastatin/Cholesterol. JAAD Case Rep..

[453] Teixeira V.B., Reis J.P., Vieira R., Tellechea Ó., Figueiredo A. (2013). Unilateral Punctate Porokeratosis—Case Report. An. Bras. Dermatol..

[454] Touraud J.P., Dalac S., Collet E., Tanter Y., Justrabo E., Dutronc Y., Lambert D. (2003). Punctate Porokeratosis in a Renal Transplant Recipient. Clin. Exp. Dermatol..

[455] Sakas E.L., Gentry R.H. (1985). Porokeratosis Punctata Palmaris et Plantaris (Punctate Porokeratosis). Case Report and Literature Review. J. Am. Acad. Dermatol..

[456] Lucke T.W., Fallowfield M., Kemmett D. (1998). A Sporadic Case of Porokeratosis Plantaris Palmaris et Disseminata. Br. J. Dermatol..

[457] Wei S.C., Yang S., Li M., Song Y.X., Zhang X.Q., Bu L., Zheng G.Y., Kong X.Y., Zhang X.J. (2003). Identification of a Locus for Porokeratosis Palmaris et Plantaris Disseminata to a 6.9-cM Region at Chromosome 12q24.1-24.2. Br. J. Dermatol..

[458] Jägle S., Juratli H.A., Hickman G., Süssmuth K., Boente M.C., Kopp J., Kirchmeier P., Zimmer A., Happle R., Bourrat E. (2021). Porokeratosis Plantaris, Palmaris et Disseminata Caused by Congenital Pathogenic Variants in the MVD Gene and Loss of Heterozygosity in Affected Skin. Acta Derm. Venereol..

[459] Topin-Ruiz S., Debarre J.-M., Blanchard E., Kettani S., Valmier P.-J., Martin L., Le Corre Y. (2017). Circumscribed palmar hypokeratosis (CPM): The diagnostic value of dermoscopy. Ann. Dermatol. Venereol..

[460] Skroza N., Proietti I., Bernardini N., Nicolucci F., Tolino E., La Viola G., Orsini D., Zuber S., Potenza C. (2012). Acitretin for Treatment of Familial Porokeratosis Palmaris et Plantaris Disseminate. Eur. J. Dermatol..

[461] McCallister R.E., Estes S.A., Yarbrough C.L. (1985). Porokeratosis Plantaris, Palmaris, et Disseminata. Report of a Case and Treatment with Isotretinoin. J. Am. Acad. Dermatol..

[462] Helfman R.J., Poulos E.G. (1985). Reticulated Porokeratosis. A Unique Variant of Porokeratosis. Arch. Dermatol..

[463] Lucker G.P., Happle R., Steijlen P.M. (1995). An Unusual Case of Porokeratosis Involving the Natal Cleft: Porokeratosis Ptychotropica?. Br. J. Dermatol..

[464] Stone N., Ratnavel R., Wilkinson J.D. (1999). Bilateral Perianal Inflammatory Verrucous Porokeratosis (Porokeratosis Ptychotropica). Br. J. Dermatol..

[465] Takiguchi R.H., White K.P., White C.R., Simpson E.L. (2010). Verrucous Porokeratosis of the Gluteal Cleft (Porokeratosis Ptychotropica): A Rare Disorder Easily Misdiagnosed. J. Cutan. Pathol..

[466] Rodríguez-Tejero A., Bueno-Rodríguez A., Montero-Vílchez T., Arias-Santiago S., Molina-Leyva A. (2020). Genitogluteal Porokeratosis: An Unusual Presentation Treated Successfully with the Novel Combination of Imiquimod 5% Cream and Photodynamic Therapy. Dermatol. Ther..

[467] Chen T.-J., Chou Y.-C., Chen C.-H., Kuo T.-T., Hong H.-S. (2006). Genital Porokeratosis: A Series of 10 Patients and Review of the Literature. Br. J. Dermatol..

[468] Shrestha S., Aryal R., Homagain S., Tiwari S.B., Rayamajhi B., Parajuli S., Paudel U. (2022). Porokeratosis of Gluteal Region: A Case Report. SAGE Open Med. Case Rep..

[469] Huang S.-L., Liu Y.-H., Chen W. (2006). Genitogluteal Porokeratosis. J. Eur. Acad. Dermatol. Venereol..

[470] Bari O., Vazirnia A., Cohen P.R., Romero L.S. (2018). Genitogluteal Porokeratosis in an HIV-Positive Man: A Case Report and Review of the Literature on Genital Porokeratosis. Dermatol. Online J..

[471] Wanat K.A., Gormley R.H., Bennett D.D., Kovarik C.L. (2012). Genitogluteal Porokeratosis Involving the Scrotum: An Unusual Presentation of an Uncommon Disease. J. Cutan. Pathol..

[472] Foran T.K., Day T., Bradford J., Scurry J. (2017). Genitogluteal Porokeratosis in a Well Woman. J. Low. Genit. Tract. Dis..

[473] Joshi R., Minni K. (2018). Genitogluteal Porokeratosis: A Clinical Review. Clin. Cosmet. Investig. Dermatol..

[474] Ryoo Y.-W., Kim Y., Yun J.-M., Kim S.-A. (2022). Porokeratosis Ptychotropica: A Case Report. J. Yeungnam Med. Sci..

[475] Scheiba N., Enk A., Proske S., Hartschuh W. (2010). Porokeratosis Ptychotropica: Successful Treatment with the Dermatome. Dermatol. Surg..

[476] Malek J., Chedraoui A., Kibbi A.G., Ghosn S. (2009). Genitogluteal Porokeratosis: 10 Years to Make the Diagnosis!. Am. J. Dermatopathol..

[477] Kumar S.S., Lee S. (2012). Genitogluteal Porokeratosis: An Unusual Clinical Presentation. Australas. J. Dermatol..

[478] Hazarika D., Pawar M. (2017). Hyperkeratotic Porokeratosis Ptychotropica with Satellite Lesions: A Rare Presentation of an Unusual Variant of Porokeratosis. Acta Dermatovenerol. Alp. Pannonica Adriat..

[479] Bari O., Calame A., Marietti-Shepherd S., Barrio V.R. (2018). Pediatric Penile Porokeratosis: A Case Report. Pediatr. Dermatol..

[480] Thomas C., Ogboli M.I., Carr R.A., Charles-Holmes R. (2003). Hypertrophic Perianal Porokeratosis in Association with Superficial Actinic Porokeratosis of the Leg. Clin. Exp. Dermatol..

[481] Sengupta S., Das J.K., Gangopadhyay A. (2008). Porokeratosis Confined to the Genital Area: A Report of Three Cases. Indian J. Dermatol. Venereol. Leprol..

[482] Robinson J.B., Im D.D., Jockle G., Rosenshein N.B. (1999). Vulvar Porokeratosis: Case Report and Review of the Literature. Int. J. Gynecol. Pathol..

[483] Kogut M., Schiller M., Hadaschik E., Enk A., Haenssle H.A. (2016). Porokeratosis Ptychotropica Involving the Glans Penis: A Unique Case of This Rare Condition. J. Dtsch. Dermatol. Ges..

[484] Qian Y.-T., Vano-Galvan S., Liu J.-W., Liu W., Ma D.-L. (2019). Porokeratosis Ptychotropica on the Penis and Scrotum: A Case Report. Acta Derm. Venereol..

[485] Yeo J., Winhoven S., Tallon B. (2013). Porokeratosis Ptychotropica: A Rare and Evolving Variant of Porokeratosis. J. Cutan. Pathol..

[486] Lacarrubba F., Musumeci M.L., Verzì A.E., Poma C., Caltabiano R., Micali G. (2021). Porokeratosis Ptychotropica: Dermoscopy, Reflectance Confocal Microscopy, and Histopathological Correlation. Indian J. Dermatol..

[487] Terrell J.R., Urban J.R., Fung M.A., Tartar D.M., Kiuru M. (2019). Pink Verrucous Plaque in a Man with Systemic Mastocytosis. Dermatol. Online J..

[488] Liu W., Liu J.-W., Ma D.-L. (2019). Porokeratosis Ptychotropica. JAMA Dermatol..

[489] Ho T., Schwentker A.R., Barron D.R., Lucky A.W. (2020). Clinical Course of Porokeratosis Ptychotropica over 7 Years in an Otherwise Healthy Child. Pediatr. Dermatol..

[490] Laino L., Pala S., Innocenzi D., Accappaticcio G., Van Steensel M.A.M. (2004). Genital Porokeratosis. Eur. J. Dermatol..

[491] Ahmed A., Hivnor C. (2015). A Case of Genital Porokeratosis and Review of Literature. Indian J. Dermatol..

[492] Tebet A.C.F., de Oliveira T.G., de Oliveira A.R.F.M., Moriya F.S., de Oliveira Filho J., Cucé L.C. (2016). Porokeratosis Ptychotropica. An. Bras. Dermatol..

[493] Xiao Y., Peng S., Mao T., Li X., Ye W., Fang M. (2022). An Unusual Case Report of Porokeratosis Ptychotropica on the Buttocks. Medicine.

[494] Tallon B., Blumental G., Bhawan J. (2009). Porokeratosis Ptychotropica: A Lesser-Known Variant. Clin. Exp. Dermatol..

[495] Pitney L., Weedon D., Pitney M. (2015). Porokeratosis Ptychotropica: A Rare Variant with Discrete Lesions. Australas. J. Dermatol..

[496] Fustà-Novell X., Podlipnik S., Combalia A., Morgado-Carrasco D., Ferrando J., Mascaró J.M., Aguilera P. (2017). Porokeratosis Ptychotropica Responding to Photodynamic Therapy: An Alternative Treatment for a Refractory Disease. Photodermatol. Photoimmunol. Photomed..

[497] Albanell-Fernández M., Luque-Luna M., López-Cabezas C., Alamon-Reig F., Espinosa-Villaseñor N., Barboza-Guadagnini L., Mascaró J.M. (2023). Treatment of Porokeratosis Ptychotropica with a Topical Combination of Cholesterol and Simvastatin. JAMA Dermatol..

[498] Mazori D.R., Shvartsbeyn M., Meehan S.A., Tarsis S.L. (2017). Transformation of Porokeratosis Ptychotropica into Invasive Squamous Cell Carcinoma. Int. J. Dermatol..

[499] Ferreira F.R., Lessa P.P., Alvarenga M.L. (2013). de Genitogluteal Porokeratosis—Case Report. An. Bras. Dermatol..

[500] Hoang N., Harper H.E., Jibbe A., Siscos S.M., Cargnel A.L., Kaplan D.L. (2020). Porokeratosis Ptychotropica: A Rare Variant That Is Commonly Misdiagnosed. Dermatol. Online J..

[501] Kulhari M., Khan H.Q., Amin S.S., Afrose R. (2022). A Rare Case of Genital Porokeratosis Associated with Epididymo-Orchitis. Indian J. Sex. Transm. Dis..

[502] Luo Y., Liu J. (2017). Image Gallery: Verrucous Porokeratosis with Characteristic Histopathological and Dermoscopic Features. Br. J. Dermatol..

[503] Zhang S. (2022). Porokeratosis Ptychotropica: A Rare Manifestation and Dermoscopic Feature. Indian J. Dermatol..

[504] Cabete J., Fidalgo A., Lencastre A., Diamantino F., João A. (2015). Porokeratosis Ptychotropica of the Scrotum: Dermoscopic Evaluation of an Atypical Presentation. An. Bras. Dermatol..

[505] Veasey J.V., Dalapicola M.C., Lellis R.F., Campaner A.B., Manzione T.d.S., Rodrigues M.C.d.F.S. (2016). Porokeratosis Ptychotropica: A Rare Manifestation with Typical Histological Exam. An. Bras. Dermatol..

[506] D’souza P., Dhali T.K., Arora S., Gupta H., Khanna U. (2014). Porokeratosis Ptychotropica: A Rare Variant of Porokeratosis. Dermatol. Online J..

[507] Lee D.K., Oh S.H., Chang S.E., Lee M.W., Choi J.H., Moon K.C., Koh J.K. (2006). A Rare Variant of Porokeratosis: Porokeratosis Ptychotropica. J. Am. Acad. Dermatol..

[508] Jee M.S., Chang S.E., Suh H.S., Choi J.H., Sung K.J., Moon K.C., Koh J.K. (2003). Porokeratosis Ptychotropica Associated with Dermal Amyloid Deposits. Clin. Exp. Dermatol..

[509] Guo H., Liu X.-Y., Li B., Gao X.-H., Chen H.-D., Li J.-H. (2015). Porokeratosis on the Scrotum: Two Cases. Dermatol. Ther..

[510] Valdivielso-Ramos M. (2008). Genital porokeratosis. Actas Dermosifiliogr..

[511] Tangoren I.A., Weinberg J.M., Ioffreda M., Werth V.P., James W.D. (1997). Penile Porokeratosis of Mibelli. J. Am. Acad. Dermatol..

[512] Ghahartars M., Zahraei S.A.H., Sari Aslani F., Hadibarhaghtalab M., Parvizi M.M. (2021). A 58-Year-Old Man with Porokeratosis Ptychotropica: A Successful Treatment with Alone Cryotherapy and Literature Review. Dermatol. Ther..

[513] Fernández Ballesteros M.D., Gómez Moyano E., Ayala Blanca M., Simonsen S. (2018). A Verrucous Plaque on the Intergluteal Cleft. Indian J. Dermatol. Venereol. Leprol..

[514] Merkle T., Hohenleutner U., Braun-Falco O., Landthaler M. (1992). Reticulate Porokeratosis--Successful Treatment with CO_2_-Laser Vaporization. Clin. Exp. Dermatol..

[515] Contreras-Ruiz J., Toussaint-Caire S., Torres-Camacho P., Villa-Castro V.B. (2018). Porokeratosis Ptychotropica: A Diagnostic and Therapeutic Challenge. J. Eur. Acad. Dermatol. Venereol..

[516] Kawakami Y., Mitsui S. (2017). A Case of Porokeratosis Ptychotropica: Successful Treatment with Topical 5% Imiquimod Cream. Clin. Exp. Dermatol..

[517] Burch H.W., Leclerq A.H., Maize J.C. (2010). Pruritic Natal Cleft Plaque. Porokeratosis Ptychotropica. Arch. Dermatol..

[518] Asawanonda P., Noppakun N., Huiprasert P. (2005). Seborrheic Keratosis-like Porokeratosis: A Case Report. Dermatol. Online J..

[519] Markantoni V., Platsidaki E., Kouris A., Aroni K., Kontochristopoulos G. (2018). Disseminated Verrucous Porokeratosis Successfully Treated with 5-FU Followed by Oral Isotretinoin—A Case Report. J. Dermat. Cosmetol..

[520] Yu Y.-F., Wu Y.-H. (2013). Verrucous Porokeratosis (Porokeratosis Ptychotropica) with Dermal Amyloid Deposits. Dermatol. Sin..

[521] Pereira N., Teixeira V., Cordeiro M.R., Tellechea O. (2013). A Rarely Diagnosed Disorder of the Gluteal Cleft. Clin. Exp. Dermatol..

[522] Neumann E. (1980). Some Unusual Findings in Porokeratosis Mibelli. Acta Derm. Venereol..

[523] Tallon B., Emanuel P. (2017). Follicular Porokeratosis, a Porokeratosis Variant. Am. J. Dermatopathol..

[524] de Almeida H.L., Guarenti I.M., de Castro L., Rocha N.M. (2007). Follicular Involvement in Porokeratosis. J. Eur. Acad. Dermatol. Venereol..

[525] Zhao M., Sanusi T., Zhao Y., Huang C., Chen S. (2015). Porokeratosis with Follicular Involvement: Report of Three Cases and Review of Literatures. Int. J. Clin. Exp. Pathol..

[526] Pongpudpunth M., Farber J., Mahalingam M. (2009). Follicular Porokeratosis: Distinct Clinical Entity or Histologic Variant?. J. Cutan. Pathol..

[527] Sud A., Shipman A.R., Odeke M., Varma K., Read-Jones M., Carr R.A. (2017). Follicular Porokeratosis: Four New Cases. Clin. Exp. Dermatol..

[528] Young P.M., Leavens J., Gaspard S., Kim G., Armstrong A.W. (2019). An Unusual Spiculated Presentation of Follicular Porokeratosis. Dermatol. Online J..

[529] Sun R., Chen H., Lian S., Zhu W. (2017). Follicular Porokeratosis: A Case Study and Literature Review. Eur. J. Dermatol..

[530] Bembry R., Louie E., Overholser E., Tirado M. (2023). Exclusive Facial Porokeratosis with Follicular Involvement in an African American Female. J. Cutan. Pathol..

[531] Kim J., Wood B.A., Harvey N.T. (2015). Follicular Porokeratosis of the Nose: Two Further Cases of an Emerging Variant of Porokeratosis. Pathology.

[532] Trikha R., Wile A., King J., Ward K.H.M., Brodell R.T. (2015). Punctate Follicular Porokeratosis: Clinical and Pathologic Features. Am. J. Dermatopathol..

[533] Chua I.S.Y., Lee J.S., Chiam L.Y. (2014). Pruritic Papules on the Nose in a 25-Year-Old Female. Indian J. Dermatol..

[534] Wang N.S., Gruson L.M., Kamino H. (2010). Facial Follicular Porokeratosis: A Case Report. Am. J. Dermatopathol..

[535] Rifaioğlu E.N., Özyalvaçlı G. (2014). Follicular Porokeratosis at Alae Nasi; A Case Report and Short Review of Literature. Indian J. Dermatol..

[536] Rocha-Sousa V.L.L., Costa J.B., de Aquino Paulo-Filho T., Rocha K.B.F., da Trindade-Neto P.B. (2011). Follicular Porokeratosis on the Face. Am. J. Dermatopathol..

[537] Gómez-Zubiaur A., Medina-Expósito I., Fernández-Flores A., Trasobares-Marugán L. (2022). Follicular Porokeratosis of the Scalp: First Description of Clinical and Trichoscopic Features. Int. J. Trichology.

[538] Kaushik A., Handa S., Chatterjee D., Vinay K., Mahajan R. (2018). Disseminated Filiform Hyperkeratosis—A Variant of Porokeratosis?. J. Eur. Acad. Dermatol. Venereol..

[539] Lee Y., Choi E.H. (2011). Exclusive Facial Porokeratosis: Histopathologically Showing Follicular Cornoid Lamellae. J. Dermatol..

[540] Walsh S.N., Hurt M.A., Santa Cruz D.J. (2007). Porokeratoma. Am. J. Surg. Pathol..

[541] Batalla A., Rosón E., De la Torre C. (2013). Porokeratoma: A Different Entity or a Variant of Verrucous (Hyperkeratotic) Porokeratosis?. Indian J. Dermatol..

[542] Sherban A.K., Fuller C.G., Griffin T., Lee J.B. (2022). Porokeratoma: A Distinct Variant of Porokeratosis. J. Cutan. Pathol..

[543] Kanitakis J., Fournier N., Jullien D. (2022). Follicular Porokeratoma: Report of a New Variant of Porokeratoma (Porokeratotic Acanthoma) and Literature Review. Am. J. Dermatopathol..

[544] Caseiro Silverio P., Pham X.-C., Kaya G. (2015). Porokeratoma: A Possible Association with Human Papillomavirus Infection. Dermatopathology.

[545] Kanitakis J., Rival-Tringali A.L., Chouvet B., Vignot E., Claudy A., Faure M. (2009). Porokeratoma (Porokeratotic Acanthoma): Immunohistological Study of a New Case. J. Cutan. Pathol..

[546] Li S.S., Compton L.A., Nemer K.M. (2021). Porokeratoma Treated with Topical 5% 5-Fluorouracil Cream. Int. J. Womens Dermatol..

[547] Tan Q., Tan C. (2015). Porokeratotic Acanthoma. J. Dtsch. Dermatol. Ges..

[548] Qian W., Xiong S., Tian X. (2021). Porokeratoma in the Nipple: A Case Report. Australas. J. Dermatol..

[549] Vigne P. (1942). Porokératose de Mibelli. Trois Cas Familiaux. Transformation Neoplasique Chez l’un d’eux. Ann. Derm. Syph..

[550] Friedman B., Golubets K., Ho J., Patton T. (2017). Linear Porokeratosis Associated with Multiple Squamous Cell Carcinomas. Cutis.

[551] Lopes L.N., Gouveia A.I., Soares-Almeida L., Sacramento-Marques M., Filipe P. (2015). Porokeratosis and Malignant Melanoma: A Causal or Incidental Association?. Indian Dermatol. Online J..

[552] Novice T., Nakamura M., Helfrich Y. (2021). The Malignancy Potential of Porokeratosis: A Single-Center Retrospective Study. Cureus.

[553] Maubec E., Duvillard P., Margulis A., Bachollet B., Degois G., Avril M.-F. (2005). Common Skin Cancers in Porokeratosis. Br. J. Dermatol..

[554] Schierbeck J., Vestergaard T., Bygum A. (2019). Skin Cancer Associated Genodermatoses: A Literature Review. Acta Derm. Venereol..

[555] Goerttler E.A., Jung E.G. (1975). Porokeratosis [Correction of Parakeratosis] Mibelli and Skin Carcinoma: A Critical Review. Humangenetik.

[556] Otsuka F., Someya T., Ishibashi Y. (1991). Porokeratosis and Malignant Skin Tumors. J. Cancer Res. Clin. Oncol..

[557] Riyaz N. (2015). Porokeratosis and Malignancy: Incidental or Causal Association?. Indian Dermatol. Online J..

[558] Kanitakis J., Euvrard S., Faure M., Claudy A. (1998). Porokeratosis and Immunosuppression. Eur. J. Dermatol..

[559] Böhm N., Sandritter W. (1975). DNA in Human Tumors: A Cytophotometric Study. Curr. Top. Pathol..

[560] Otsuka F., Shima A., Ishibashi Y. (1989). Porokeratosis as a Premalignant Condition of the Skin. Cytologic Demonstration of Abnormal DNA Ploidy in Cells of the Epidermis. Cancer.

[561] Imakado S., Otsuka F., Ishibashi Y., Ohara K. (1988). Abnormal DNA Ploidy in Cells of the Epidermis in a Case of Porokeratosis. Arch. Dermatol..

[562] Otsuka F., Chi H.I., Shima A., Ishibashi Y. (1988). Cytological Demonstration of Abnormal DNA Ploidy in the Epidermis of Porokeratosis. Arch. Dermatol. Res..

[563] Beers B., Jaszcz W., Sheetz K., Hogan D.J., Lynch P.J. (1992). Porokeratosis Palmaris et Plantaris Disseminata. Report of a Case with Abnormal DNA Ploidy in Lesional Epidermis. Arch. Dermatol..

[564] Leão R., Apolónio J.D., Lee D., Figueiredo A., Tabori U., Castelo-Branco P. (2018). Mechanisms of Human Telomerase Reverse Transcriptase (hTERT) Regulation: Clinical Impacts in Cancer. J. Biomed. Sci..

[565] Park H.-R., Min S.K., Cho H.D., Kim K.H., Shin H.S., Park Y.E. (2004). Expression Profiles of P63, P53, Survivin, and hTERT in Skin Tumors. J. Cutan. Pathol..

[566] Sharpless N.E., Sherr C.J. (2015). Forging a Signature of in Vivo Senescence. Nat. Rev. Cancer.

[567] Sakiz D., Turkmenoglu T.T., Kabukcuoglu F. (2009). The Expression of P63 and P53 in Keratoacanthoma and Intraepidermal and Invasive Neoplasms of the Skin. Pathol. Res. Pract..

[568] Arranz-Salas I., Sanz-Trelles A., Ojeda D.B. (2003). P53 Alterations in Porokeratosis. J. Cutan. Pathol..

[569] Urano Y., Sasaki S., Ninomiya Y., Oura H., Arase S. (1996). Immunohistochemical Detection of P53 Tumor Suppressor Protein in Porokeratosis. J. Dermatol..

[570] Kim K.H., Park E.J., Seo Y.J., Cho H.S., Kim C.W., Kim K.J., Park H.R. (2006). Immunohistochemical Study of Cyclooxygenase-2 and P53 Expression in Skin Tumors. J. Dermatol..

[571] Ninomiya Y., Urano Y., Yoshimoto K., Iwahana H., Sasaki S., Arase S., Itakura M. (1997). P53 Gene Mutation Analysis in Porokeratosis and Porokeratosis-Associated Squamous Cell Carcinoma. J. Dermatol. Sci..

[572] Pietkiewicz P., Gornowicz-Porowska J., Bowszyc-Dmochowska M., Jagielska J., Helak-Łapaj C., Kaczmarek E., Dmochowski M. (2014). Discordant Expression of Desmoglein 2 and 3 at the mRNA and Protein Levels in Nodular and Superficial Basal Cell Carcinoma Revealed by Immunohistochemistry and Fluorescent in Situ Hybridization. Clin. Exp. Dermatol..

[573] Mowla S.N., Lam E.W.-F., Jat P.S. (2014). Cellular Senescence and Aging: The Role of B-MYB. Aging Cell.

[574] Scola N., Skrygan M., Wieland U., Kreuter A., Gambichler T. (2012). Altered Gene Expression in Squamous Cell Carcinoma Arising from Congenital Unilateral Linear Porokeratosis. Clin. Exp. Dermatol..

[575] Uryu M., Furue M. (2017). p16INK4a Expression in Porokeratosis. Ann. Dermatol..

[576] Jaiswal P.K., Goel A., Mittal R.D. (2015). Survivin: A Molecular Biomarker in Cancer. Indian J. Med. Res..

